# Virus‐Mediated Self‐Assembly of Functional Cyclodextrins for Antiviral Inhibition

**DOI:** 10.1002/anie.3935543

**Published:** 2026-07-15

**Authors:** Pedro J. Hernando, Laora Boulo, Léonid Lavnevich, Elisa Teyssou, Stéphane Marot, Adélie Gothland, Jean‐Michel Guigner, Anne‐Geneviève Marcelin, Mickaël Ménand, Vincent Calvez, Matthieu Sollogoub

**Affiliations:** ^1^ Sorbonne Université, CNRS Institut Parisien de Chimie Moléculaire, IPCM Paris France; ^2^ Sorbonne Université, Pitié‐Salpêtrière Hospital Department of Virology Institut Pierre Louis D'epidémiologie Et De Santé Publique, AP‐HP INSERM Paris France; ^3^ Sorbonne Université, CNRS, MNHN Institut de Minéralogie de Physique des Matériaux et de cosmochimie, IMPMC Paris France; ^4^ Institut Universitaire de France Paris France

## Abstract

We report a virus‐mediated supramolecular assembly as an adaptive inhibitor of viral infection. Building on cyclodextrin (CD) host–guest self‐assembly, we designed a monomer bearing an adamantyl unit to promote inclusion‐mediated polymerization, a bridge across the CD cavity to prevent self‐inclusion, and a sialic acid (SA) ligand to engage spike glycan‐recognition sites. A series of SA‐functionalized self‐assembling CDs was synthesized, together with nonassembling and nonbinding controls. In cellular infection assays, the SA‐functionalized self‐assembling CD inhibited SARS‐CoV‐2‐induced cytopathic effect (CPE) without detectable cytotoxicity, whereas controls were inactive, indicating that both self‐assembly and ligand recognition are required for antiviral activity. NMR and cryo‐electron microscopy studies of the active SA‐functionalized self‐assembling CD show that the viral surface mediates supramolecular polymer growth by nucleating cooperative supramolecular polymerization through ligand–receptor recognition. Inhibition was maintained across multiple SARS‐CoV‐2 variants, consistent with adaptive assembly. Coassembly with an unfunctionalized self‐assembling CD preserved strong inhibition at low ligand fractions, supporting heteropolymer formation with optimized ligand spacing. These findings establish virus‐mediated supramolecular polymerization of functional CDs as a modular antiviral platform.

## Graphical abstract

A sialic acid (SA)‐functionalized cyclodextrin (CD) self‐assembles on SARS‐CoV‐2 through ligand–receptor recognition, nucleating cooperative supramolecular polymer growth that blocks infection. The adaptive assembly requires both ligand binding and host–guest polymerization, remains active across variants, and can be tuned by coassembly, establishing functional CDs as modular antiviral materials.

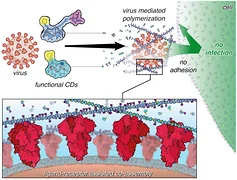

## 1 Introduction

Multivalency is a powerful strategy to enhance molecular recognition toward biological targets such as proteins, viruses, and bacteria [1, 2, 3, 4]. By displaying multiple ligands on a single scaffold, whether oligomeric [5, 6, 7], dendritic [8, 9, 10], or polymeric [11, 12], strong and specific binding can be achieved through cooperative effects. However, this approach relies on precise scaffold design to match the target geometry, requiring fine control over size, valency, and spatial organization [13, 14]. Each new target, therefore, demands a new molecular design, limiting general applicability. Despite significant efforts to rationalize scaffold design, predicting optimal architectures remains a major challenge [15]. Consequently, the development of adaptive multivalent systems, capable of reorganizing themselves in response to their target, represents an attractive alternative. Supramolecular systems, and in particular supramolecular polymers [16, 17, 18, 19, 20], inherently possess the dynamic and reversible characteristics required for adaptivity. Ideally, polymerization, composition, and architecture of the supramolecular polymer would be directly induced by the biological target itself, such as a virus or a bacterium, allowing spontaneous self‐adjustment for optimal binding.

**FIGURE 1:**
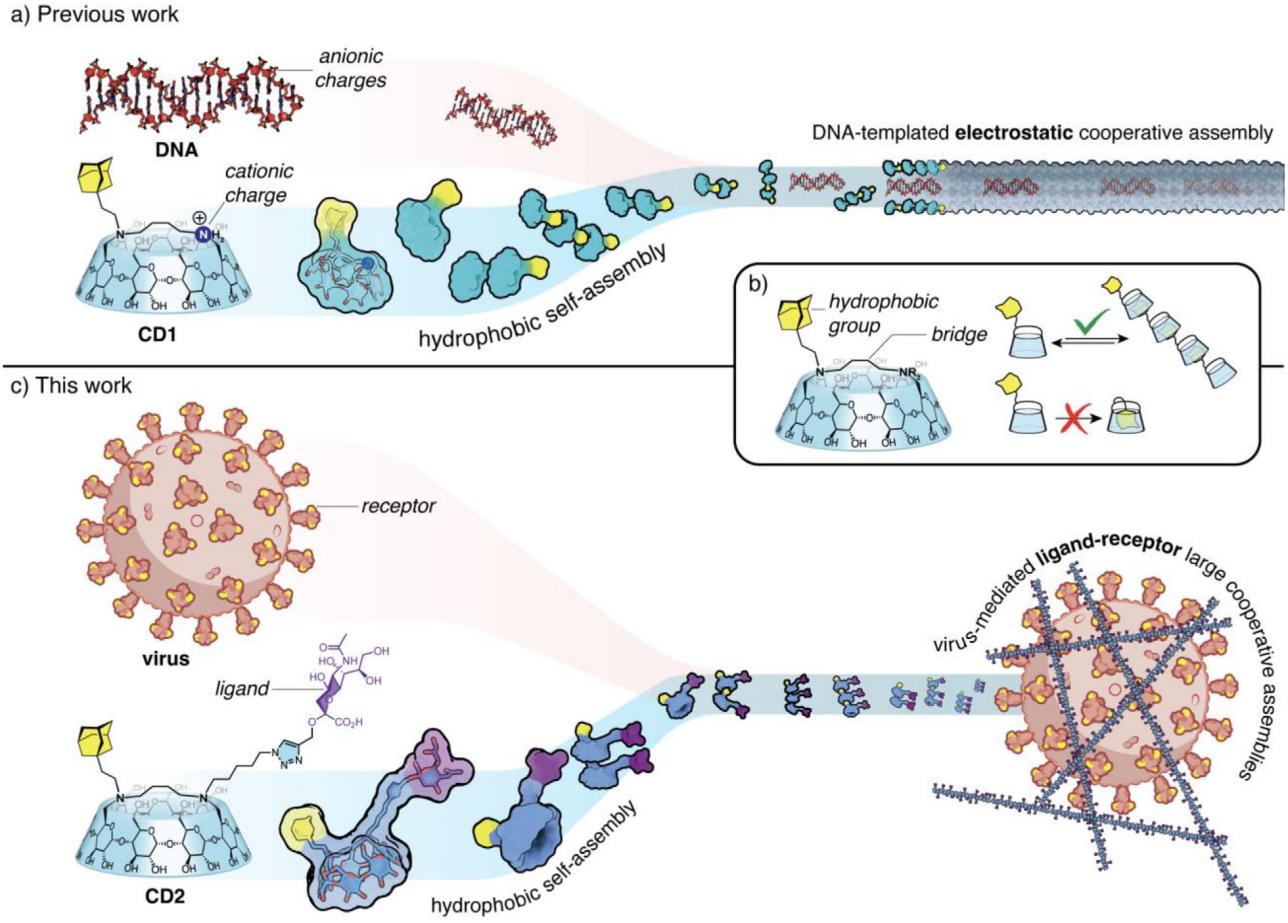
(a) *Previous work*. **CD1** self‐assembles through the inclusion of its hydrophobic group in the cavity of the next **CD1**, forming dimers at 500 µM. In contrast, at the same concentration, electrostatic interactions between the ammonium groups of **CD1** and DNA drive the formation of much larger assemblies by cooperative interaction. (b) Bridging across the CD cavity prevents self‐inclusion of the hydrophobic group appended on the CD torus. (c) *Design of the present work*. **CD2** retains the functionalities required for self‐assembly but replaces the electrostatic interaction with a ligand–receptor interaction. The virus, which displays multiple copies of the receptor, acts as a promoter for the formation of large self‐assembled species, thereby preventing infection by inhibiting viral attachment.

We previously reported a cyclodextrin (CD)‐based building block capable of self‐assembling into small supramolecular oligomers. We demonstrated that these assemblies can grow into much larger structures through cooperative effects when a secondary, lateral interaction is introduced [21]. Specifically, we showed that **CD1** can form very long supramolecular polymers through the combination of the hydrophobic effect and electrostatic interactions. Indeed, the grafting of an adamantane unit attached to **CD1** promotes the initial assembly through iterative inclusion in the cavity of the next CD, and the ammonium group borne by **CD1** allows for coulombic interactions with a negative charge of a double‐stranded DNA (Figure 1a) [22]. Here, the DNA therefore acts as a molecular template. This cooperative self‐coassembly is a promising design for adaptive multivalent systems, where the presence of a biological target could trigger supramolecular polymerization and organization of a functionalized monomer. Replacing the electrostatic interaction with a ligand–receptor binding could, in principle, induce a similar cooperative polymerization, provided that multiple receptors are present and close enough to trigger and support assembly.

Viruses represent ideal templates for such processes. Their surfaces display dense arrays of recognition sites enabling multivalent attachment to host cells. For instance, SARS‐CoV‐2 engages cell‐surface sialic acids (**SA**s) and ACE2 receptors via multiple copies of its trimeric spike (S) protein [23, 24]. Several multivalent scaffolds have exploited this feature, including sialic acid‐decorated calix[4]arenes [25] and heptavalent β‐CDs [26], which inhibit viral adhesion more effectively than monovalent analogues. In a seminal work, Bovin showed that smaller assemblies of tetravalent functional peptides could grow in the presence of a virus [27]. Recently, Haag described 2D nanosheets based on polyanionic self‐assembled dendritic polymers capable of wrapping and shielding viruses [28, 29, 30]. These examples underscore the potential of multivalent and supramolecular approaches to disrupt viral attachment.

Building upon these insights, we aim to design self‐assembling, adaptive supramolecular systems whose formation and architecture are mediated by the virus itself, thus combining the specificity of multivalent recognition with the dynamic responsiveness of supramolecular chemistry and resulting in the inhibition of the viral attachment. More specifically, drawing from our previous work, we designed a self‐assembling **CD2** derivative that incorporates an adamantyl group to promote assembly, a bridging unit across the CD cavity to prevent self‐inclusion (Figure 1b), and an appended arm bearing a ligand capable of recognizing receptors on the viral surface. Here, we show that this design effectively inhibits SARS‐CoV‐2 cell infection through the synergistic action of host‐guest and ligand‐receptor interactions. (Figure 1c).

## 2 Results and Discussion

**SCHEME 1:**
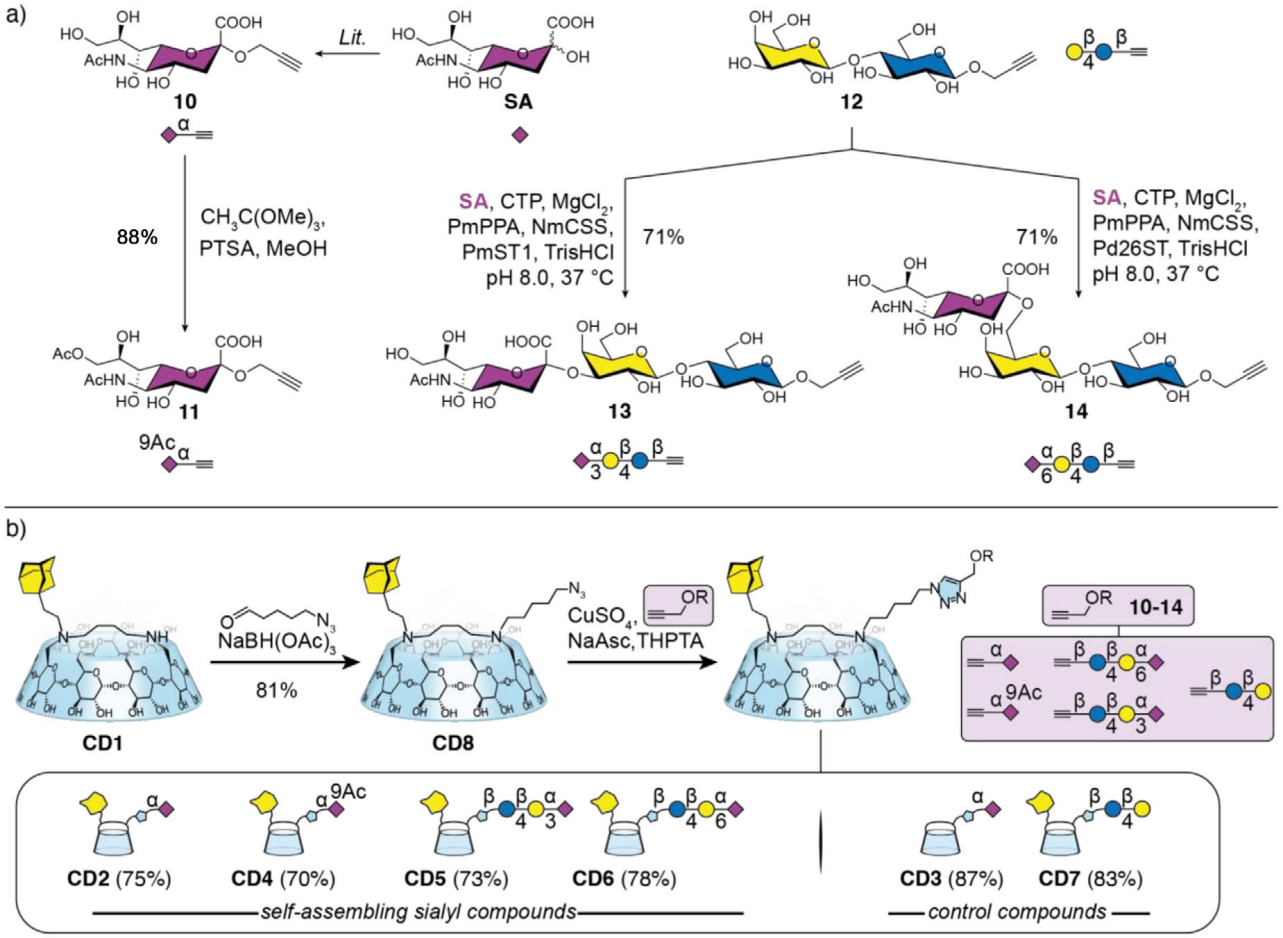
(a) Synthesis of propargylated sugar derivatives **10–14** for conjugation with self‐assembling CDs. (b) Synthesis of a small library of CDs by CuAAC.

A series of self‐assembling CDs bearing a ligand was synthesized from the previously published **CD1** [21] on which an additional reductive amination was performed between the secondary amine of **CD1** and 5‐azidopentanal to afford the clickable azido‐functionalized **CD8** with a 81% yield (Scheme 1b). Next, we designed a small collection of propargylated derivatives of SA, with the aim of mimicking naturally occurring epitopes of the glycan‐binding domain (GBD, also referred to as N‐terminal domain‐NTD) of the S protein of SARS‐CoV‐2 [31]. For this, we envisioned four different structures: *N‐*acetylneuraminic acid (SA), its 9‐*O‐*acetyl derivative (9AcSA), as well as 3′‐ and 6′‐sialyllactose (3′ and 6′SL) (Scheme 1a).

A classical route was followed to obtain propargyl SA (**10**) [32, 33], and its reaction with trimethyl orthoacetate and p‐toluenesulfonic acid (PTSA) afforded 9AcSA (**11**) [25] in 88% yield [34]. Two sialyl lactosides were synthesized using a chemo‐enzymatic approach starting from a known propargyl lactoside (**12**) [35]. Selective 3′‐ and 6′‐ sialylation of **12** used a one‐pot three‐enzyme method adapted from literature [36, 37], employing *Pasteurella multocida* α‐2,3‐sialyltransferase (PmST1) for the selective synthesis of propargyl 3’SL (**13**), and *Photobacterium damsel* α‐2,6‐sialyltransferase (Pd26ST) for the selective synthesis of propargyl 6’SL (**14**). Essentially, the pathway involves the formation of a cytidine monophosphate (CMP)‐SA donor by CMP‐SA synthetase from *Neisseria meningitidis* (NmCSS), which is then employed by either of the sialyltransferases, together with acceptor **12**, for the selective synthesis of **13** or **14**. The purification of **13** and **14** involved flash chromatography on silica gel to afford both derivatives with a 71% yield. In agreement with literature [38], comparison of both ^1^H‐NMR spectra allows to confirm the selectivity of the reaction by looking at the H‐3 proton shifts of SA, being slightly downfield shifted for the α‐2,3′ product [*δ*(H‐3eq) = 2.77 ppm and *δ*(H‐3ax) = 1.81 ppm] in comparison with the α‐2,6′ product [*δ*(H‐3eq) = 2.74 ppm and *δ*(H‐3ax) = 1.76 ppm] (Scheme 1a).

With all partners in hand, we conjugated **CD8** with the sialylated derivatives **10**, **11**, **13**, and **14** using a standard copper‐catalyzed azide‐alkyne cycloaddition (CuAAC) with copper(II) sulphate, tris(3‐hydroxypropyltriazolylmethyl)amine (THPTA), and sodium ascorbate in water (Scheme 1b). Initial purification attempts were complicated by strong chelation of copper by the SA moiety in the products, which hindered the isolation of copper‐free compounds; this bidentate chelation via the SA carboxylate has been previously described [39]. To enable efficient copper removal, we adapted the method of Hartmann and coworkers [40], employing sodium diethyldithiocarbamate (DDC) to form a poorly water‐soluble 1:2 Cu:DDC complex. After completion of the CuAAC reaction, treatment with DDC precipitated CuDDC_2_, which was largely removed by centrifugation, while the residual, intensely yellow‐to‐brown complex was eliminated by reverse‐phase chromatography. This protocol provided the final functionalized CDs bearing SA (**CD2**, 75%), 9AcSA (**CD4**, 70%), 3′SL (**CD5**, 73%), and 6′SL (**CD6**, 78%). Residual copper was quantified by reacting aliquots of **CD2** and **CD4‐6** with DDC and measuring the absorbance of the CuDDC_2_ complex at 450 nm against a calibration curve, giving 2–7 µM in biological assays (see Section S3), an acceptable, noncytotoxic concentration.

For control purposes, we also synthesized three additional CDs: **CD3**, which presents the SA motif but lacks the adamantyl group and is therefore unable to self‐assemble; **CD7**, which bears the adamantyl group enabling self‐assembly, but contains lactose that is not recognized by protein S (Scheme 1b).

**FIGURE 2:**
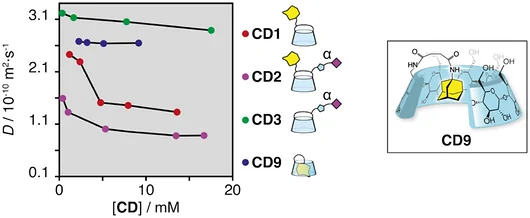
Concentration dependence of the diffusion coefficient (DOSY, 600 MHz, D_2_O, 300 K) for **CD1‐3** and **CD9**.

We fully characterized these products both by mass spectrometry and NMR spectroscopy. In particular, we assessed the ability of **CD2** to self‐assemble using DOSY‐NMR experiments to verify that the functionalization of the second amine on the bridge does not prevent self‐assembly. We plotted the diffusion coefficient (*D*) against concentration for **CD1**, **CD3**, and **CD9**, a CD known to undergo self‐inclusion preventing supramolecular polymerization (Figure 2) [41]. As anticipated, the diffusion coefficient of the adamantyl‐free **CD3** shows minimal sensitivity to changes in concentration, remaining within the same magnitude reported for other monomers that do not self‐assemble, such as **CD9**, across a comparable concentration range (D ≈ 2.6 × 10^−10^ m^2^ s^−1^) [42]. In contrast, increasing the concentration of **CD2** produces a nonlinear reduction in its diffusion coefficient, consistent with the formation and growth of an isodesmic supramolecular polymer [43]. Using the Tirado‐García de la Torre approach [43], the degree of polymerization (DP) was estimated to reach approximately 14 units at 17 mM, with DP = 1 at *c* < 1 mM (see Section S4).

**FIGURE 3:**
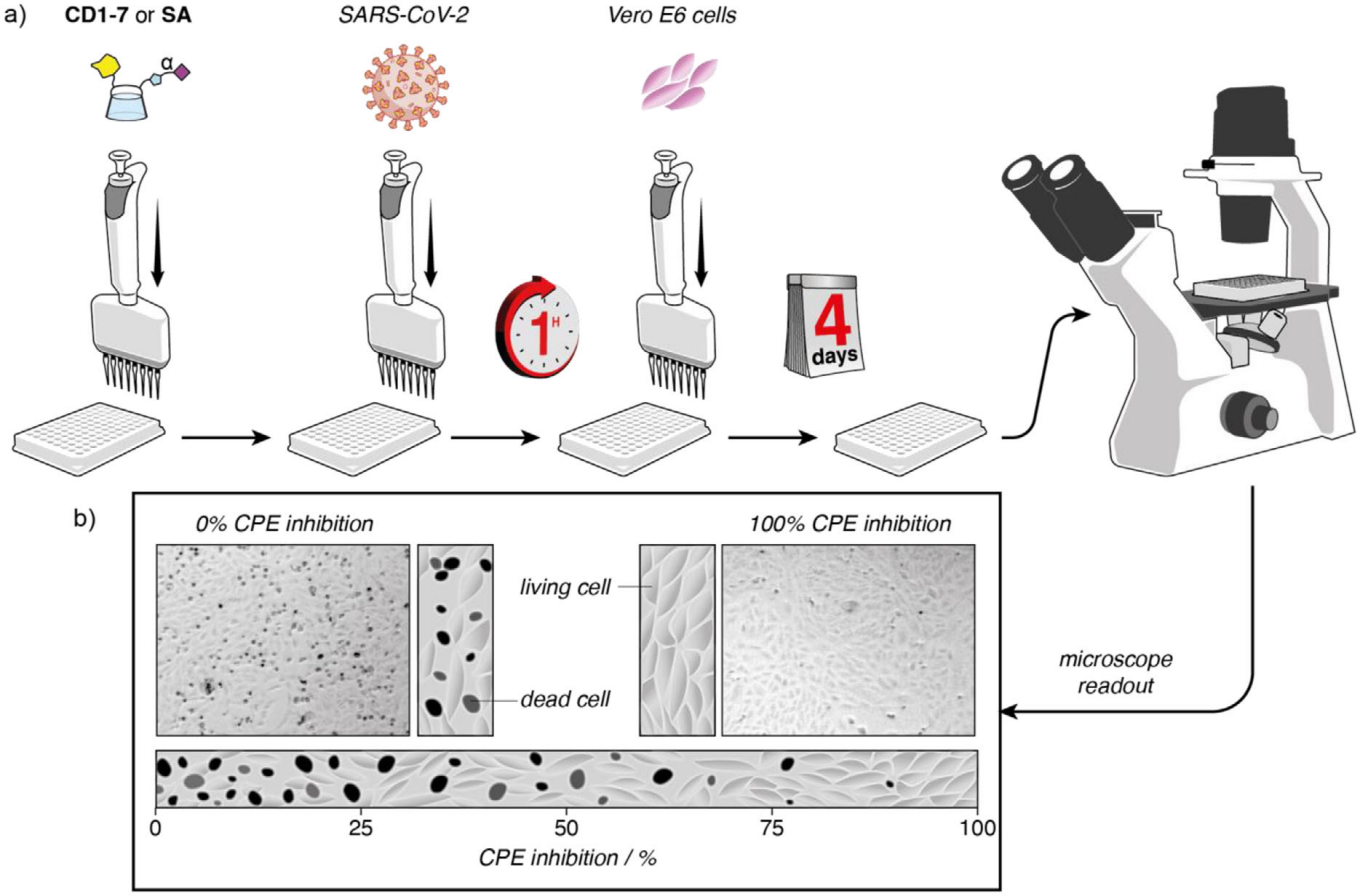
CPE inhibition assay protocol to assess antiviral activity of CD Compounds (**CD1‐7**) or **SA** derivatives against SARS‐CoV‐2. (a) Preparation procedure for the 96‐well plates before optical microscopic assessment. (b) Two representative micrographs of Vero E6/TMPRSS2 cells with or without inoculation of SARS‐CoV‐2. The left image shows a representative positive‐control well containing SARS‐CoV‐2 only, corresponding to the minimum CPE inhibition score, whereas the right image shows a representative negative‐control well without SARS‐CoV‐2. Each condition (CD or SA derivative) was tested in eight replicates. Wells were scored for CPE by comparison with the controls, using an infectivity score ranging from 0 (no CPE) to 4 (75% to 100% of the cell monolayers were displaying CPE). The mean CPE score (%CPE) was then used to calculate inhibition according to the following equation: Inhibition (%) = 100 – %CPE.

To evaluate the effect of these self‐assembling sialylated CD derivatives on SARS‐CoV‐2, we assessed their ability to inhibit viral infection in Vero E6 cells expressing TMPRSS2 (Vero E6/TMPRSS2) (Figure 3, and Section S5). Our working hypothesis was that, if these derivatives interact with the GBD, the resulting complexes would sterically hinder viral entry. To this end, we performed a cytopathic effect (CPE) inhibition assay in the presence of the CDs, where CPE is defined as the morphological alterations in cell cultures associated with virus‐induced cell death. CPE was scored by visual inspection of adherent Vero E6/TMPRSS2 cells monolayers under brightfield microscopy, where disruption of the monolayer appears as dark spots among cells and refringent floating cells (Figure 3b) [44, 45]. Vero E6/TMPRSS2 cells were chosen because of their high susceptibility to viral infection and their widespread use in SARS‐CoV‐2 research and in virology more broadly [46].

**FIGURE 4:**
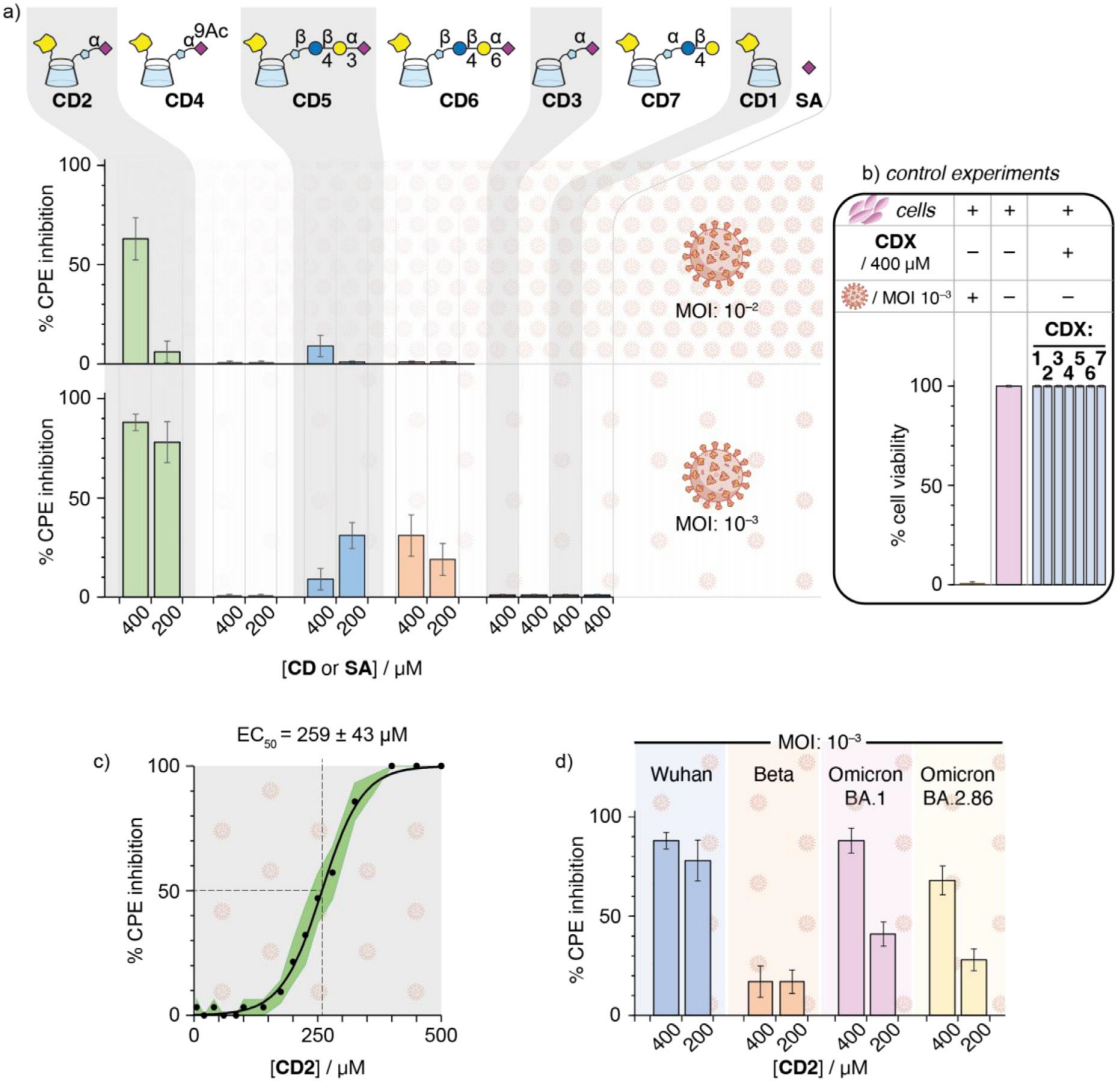
(a) CPE inhibition activity of the CD compounds against SARS‐CoV‐2 (Wuhan variant, MOI 10^−2^ and 10^−3^) after 4 days of incubation in the presence of **CD1–CD7** or **SA**, either at 200 or 400 µM. (b) CDs compounds cytotoxicity assessment in Vero E6/TMPRSS2 cells—the signs “−” and “+” indicate the presence or absence of the corresponding item. Negative control (left) carried out with SARS‐CoV‐2 and positive control (right) carried out without SARS‐CoV‐2. Representative cytotoxicity assay (center) for **CD1–CD7** performed separately for each CD compound—no CD compound exhibited cytotoxicity. (c) **CD2** EC_50_ determination (Wuhan variant MOI 10^−3^). (d) CPE inhibition activity of the CD compounds against four different SARS‐CoV‐2 variants at a MOI of 10^−3^ (Wuhan, Beta, Omicron BA.1 and Omicron BA.2.86) after 4 days of incubation in the presence of **CD2** either at 200 or 400 µM. Error bars represent the standard error obtained from eight replicates.

For the biological evaluation, we assessed the antiviral activity of the synthesized sialylated derivatives **CD2** and **CD4‐6** at two concentrations (200 and 400 µM; Figure 3a). Each compound was pre‐incubated with SARS‐CoV‐2 for 1 h at 37°C in 5% CO_2_ prior to infection. SARS‐CoV‐2 variants (Wuhan [B.1, D614G], Beta, Omicron BA.1, and BA.2.86) were isolated from molecularly confirmed hospitalized COVID‐19 patients by inoculating positive respiratory samples onto Vero or Vero E6/TMPRSS2 cells. Infections were performed at two multiplicities of infection (MOIs), 10^−2^ and 10^−3^, corresponding to approximately 1 virion per 100 or 1000 cells, respectively, corresponding to the estimated infectious inoculum observed in humans [47, 48, 49]. Following pre‐incubation, the virus‐compound mixtures were added to Vero E6/TMPRSS2 cells in a 96‐well plate at a cell density of 10^4^ cells per well, and incubated at 37°C in 5% CO_2_ for 4 days. Percent CPE inhibition was deduced from an infectivity score that was assigned to each well (Figure 3). In parallel, the seven CDs (**CD1‐CD7**) showed no detectable cytotoxicity in this cell line at concentrations up to 400 µM (Figure 4b).

Notably, SA‐functionalized **CD2** showed strong inhibition of SARS‐CoV‐2‐induced CPE (Figure 4a). At an MOI of 10^−3^, CPE inhibition reached 88 ± 4% and 78 ± 10% at 400 and 200 µM, respectively. When the MOI was increased to 10^−2^, inhibition remained substantial at 63 ± 11% for 400 µM, but dropped to 6 ± 5% at 200 µM. The half‐maximal effective concentration (EC_50_) of **CD2** was determined to be 259 ± 43 µM (Figure 4c).

To dissect the origin of this activity, we performed a series of control experiments to probe the cooperative contribution of hydrophobic interactions between adamantyl groups and β‐CD cavities, which drive self‐assembly, and the SA ligand, which targets the S protein. Free SA at the same concentrations did not inhibit CPE. Likewise, nonassembling **CD3**, which carries the SA ligand but lacks the adamantyl unit, showed no inhibition at 400 µM and at the lowest MOI (10^−3^). To confirm the requirement for a targeting ligand, we tested **CD1**, which also displayed no inhibitory effect. As an additional control, lactose‐functionalized **CD7** was evaluated and similarly showed no activity. None of the CD derivatives tested exhibited cytotoxicity at any of the concentrations employed (Figure 4b). All this data demonstrates that both hydrophobic assembly and ligand–receptor recognition are required for CPE inhibition.

**FIGURE 5:**
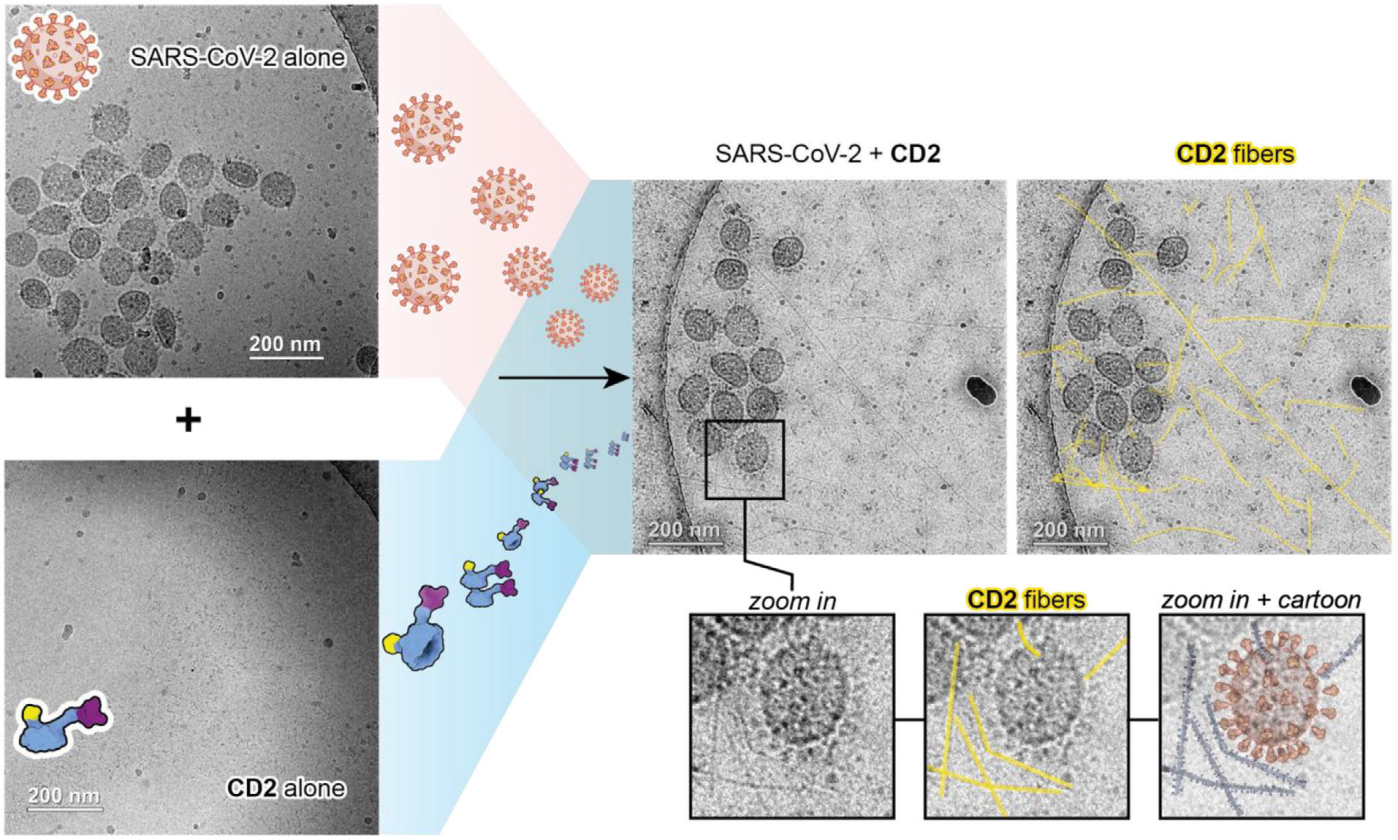
cryo‐EM micrographs of UV‐inactivated SARS‐CoV‐2 virions, of **CD2** alone (1 h maturation), and of **CD2** in the presence of UV‐inactivated SARS‐CoV‐2 virions (1 h incubation). Zoom and cartoon representation of the virus and self‐assembly of **CD2**.

To investigate the effect of this combination of interactions on the self‐assembly of **CD2** in the presence of the virus, we performed cryo‐electron microscopy (cryo‐EM) experiments. After 1 h of incubation of **CD2** with UV‐inactivated SARS‐CoV‐2 virions, long fibers were detected (Figure 5). These fibers appear relatively stiff, and polymerization occurs around the virions but also extends beyond the viral particles. Since neither free SA nor the monovalent SA‐presenting **CD3** inhibits CPE, we infer that the viral surface itself somehow promotes supramolecular polymer growth. However, from these images, it is difficult to conclude that the virus actually serves as a template for the polymerization, as in classical template‐mediated assembly, the template supports and directs polymer growth. To better understand this phenomenon, we performed additional cryo‐EM experiments with **CD2** in the absence of virus, following its self‐assembly over time under conditions relevant to the biological assays: 10 min, corresponding to the preparation time of the **CD2** solution before mixing with the virus; 1 h, corresponding to the incubation time with the virus; and 4 days, corresponding to the duration of the **CD2**‐virus incubation with cells. At 10 min and 1 h, no fibers were observed. In contrast, after 4 days, long **CD2** fibers were detected (see Section S6). These results show that **CD2** can self‐assemble into long fibers even in the absence of a virus, but only after a significant lag time. The formation of fibers much longer than expected for the isodesmic process characterized by NMR implies a transition toward a cooperative assembly mechanism involving nucleation followed by elongation [50]. In this type of mechanism, nucleation is typically associated with a lag time [51], consistent with our observation that no assemblies are detected within the 1st hour, whereas long fibers are observed at later times. We therefore propose that the virus accelerates fiber formation by promoting nucleation of the cooperative supramolecular polymerization. In our hypothesis, binding sites on the S protein, despite the modest affinity of monomeric SA for this protein (Kd ca. 10 mM) [52, 53], recruit **CD2** monomers and/or short oligomers, locally increasing their concentration and thereby favoring the formation of nuclei, which subsequently elongate into long fibers according to a cooperative polymerization mechanism [54]. CPE inhibition, together with cryo‐EM observations showing **CD2** fiber formation in the presence of virions, supports a mechanism in which **CD2** interferes with early virus‐cell interactions, most likely by reducing viral attachment through interaction with SA‐recognition sites on the viral surface [25].

In order to enhance the antiviral effect of **CD2**, we explored two different variants of the ligand using lactose‐bound SA (α‐2,6'‐SA and α‐2,3'‐SA, Scheme 1) [55]. However, none of the modified **CD5‐6** outperformed **CD2** (Figure 4a). For the Wuhan variant, **CD2** bearing only the SA motif was the most effective ligand in this series at preventing in vitro infection, whereas carbohydrate‐core modifications reduced activity, likely due to presentation or steric effects rather than affinity differences, as also reported by others [56].

We next turned our attention towards variants of SARS‐CoV‐2 to evaluate the robustness of the interaction of **CD2** with different spike structural variations. We thus performed tests on the Beta, BA.1 (Omicron), and BA.2.86 (Omicron) variants (Figure 4e). For the Beta variant, the CPE inhibition by **CD2** decreases to 17 ± 8% and 17 ± 6 (MOI of 10^−3^, at both 200 and 400 µM), suggesting mutagenic changes in the affinity of SARS‐CoV‐2 for SA. However, the inhibitory effect of our supramolecular scaffold is conserved for the Omicron variants, achieving up to 88 ± 6% and 68 ± 7% for BA.1 and BA.2.86, respectively (Figure 4d). This encouraging result shows that **CD2** can be highly efficient against different SARS‐CoV‐2 variants, especially those currently circulating in the human population (Omicron sub‐lineages). It also shows that the self‐assembling **CD2** can adapt to different contexts, certainly due to its dynamic nature.

**FIGURE 6:**
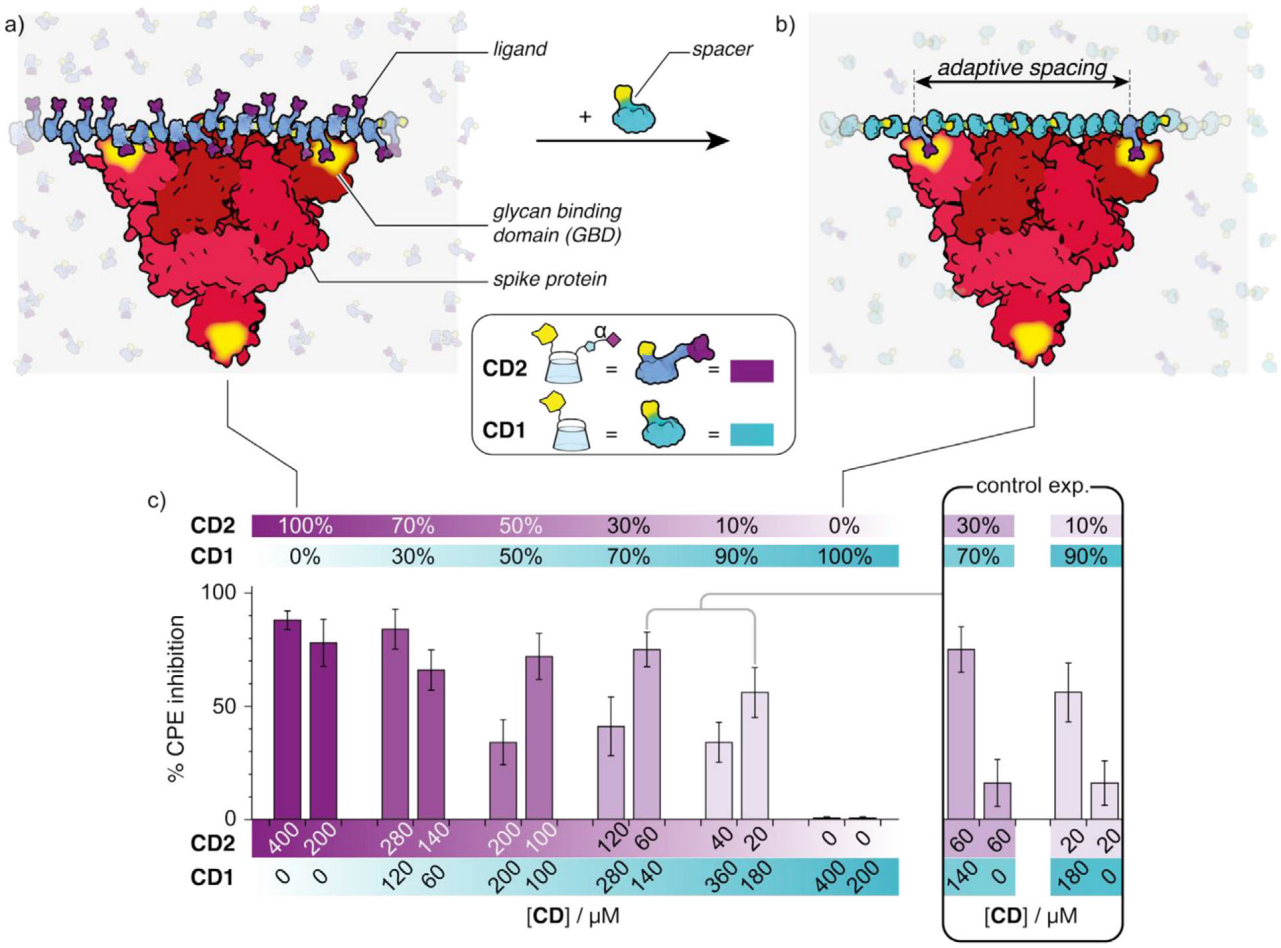
Evaluation of the CPE inhibition activity of coassemblies combining sialylated **CD2** and unfunctionalized **CD1** at various ratios as inhibitors of SARS‐CoV‐2‐induced CPE in Vero E6/TMPRSS2 cells. (a) Schematic representation of potential assemblies on the surface of the S protein with (a) 100% sialylated **CD2** and (b) a mixture of **CD2** with a high proportion of **CD1** spacer. (c) CPE inhibition assessment of Vero E6/TMPRSS2 cells in the presence of SARS‐CoV‐2 (Wuhan variant, MOI 10^−3^) and different ratios of **CD2** and **CD1** at 400 or 200 µM total CD concentrations. Inset: Comparison of CPE inhibition by **CD2** with or without **CD1** spacer performed for ratios of 30% and 10% **CD2** and 200 µM total CD concentration. Error bars represent the standard error obtained from three independent runs with eight replicates each.

Considering an interaction of a supramolecular homopolymer of **CD2** with the S protein, we estimated that not all of the **CD2** units were necessary for ligand–receptor interaction. Indeed, since the GBD sites are separated by several nanometers on the S protein, some of the **CD2** units probably act solely as spacers. Moreover, high ligand densities have been shown to hinder multivalent interactions, with optimal binding achieved only when the geometry and degree of substitution are well matched. We therefore mixed the SA‐functionalized **CD2** with the unfunctionalized **CD1** and evaluated the ability of the resulting heteropolymer to inhibit CPE, anticipating that the most thermodynamically stable assembly, that is, the one best suited for efficient interaction, would emerge. (Figure 6a,b).

We assessed CPE inhibition at an MOI of 10^−^^3^ (SARS‐CoV‐2 Wuhan variant) using mixtures containing 100%, 70%, 50%, 30%, 10%, or 0% **CD2**, complemented with the **CD1** spacer to reach a total CD concentration of either 400 or 200 µM (Figure 6c–e). Notably, full SA functionalization is not required to sustain strong CPE inhibition: 50% inhibition is maintained with as little as 10% active ligand, corresponding to 20 µM **CD2**. The most striking result was observed for 60 µM **CD2** combined with 140 µM **CD1**, which yielded 75% CPE inhibition. Importantly, **CD2** alone is inactive at 20 and 60 µM (Figure 6e). These data indicate that effective inhibition does not require full functionalization of the supramolecular polymer with the SA motif. Instead, the antiviral activity under these conditions arises from the supramolecular heteropolymer rather than from **CD2** alone, enabling a lower fraction of the more complex monomer **CD2** while preserving robust CPE inhibition.

These findings highlight the potential of virus‐mediated supramolecular polymerization as a strategy for antiviral inhibition. In this approach, the viral surface is not merely a target but also promotes a local increase in the concentration of functional CDs, inducing nucleation of the cooperative supramolecular polymerization into extended structures capable of blocking viral attachment. Beyond SA, several other ligands of the SARS‐CoV‐2 S protein have been reported, including peptides [57], heparin [58], and heparan sulfate analogues [59], as well as small molecules [60] that interfere with protein–protein interactions. Because these ligands can target distinct regions of the S protein, one can envision the development of a library of functional CDs that self‐assemble synergistically on the viral surface to enhance binding avidity and antiviral efficacy. In such designs, however, the affinity of the monomeric species must be carefully tuned: interactions that are too weak may not efficiently nucleate assembly, whereas excessively strong interactions may reduce the dynamicity required for adaptive supramolecular growth. Thus, balancing ligand affinity, multivalency, and reversibility will be essential for the rational design of next‐generation self‐assembling antiviral systems.

## 3 Conclusion

In conclusion, we show that a supramolecular polymer is capable of inhibiting SARS‐CoV‐2 viral infection in vitro. We prepared a set of self‐assembling *β*‐CD derivatives carrying SAs and evaluated their ability to prevent SARS‐CoV‐2 infection in Vero E6/TMPRSS2 cells, achieving up to 88% CPE inhibition. The results show that the hydrophobic effect driving the self‐assembly of **CD2**, together with the SA‐receptor interaction, is both essential for the antiviral effect and enables an original virus‐mediated self‐assembly. We also show that the system inhibits infection by several current SARS‐CoV‐2 variants, highlighting the role of its dynamic nature in providing adaptability to viral changes. This adaptability is further supported by the performance of supramolecular heteropolymers, which retain antiviral activity even when the proportion of **CD2** is set at a concentration that would otherwise be inactive if used alone. Overall, these findings pave the way for developing a variety of monomers that can be combined at will to target multiple sites within a single pathogen for synergistic effects or to address other pathogens altogether.

## Author Contributions

**Pedro J. Hernando**: investigation, writing – original draft. **Laora Boulo**: investigation. **Léonid Lavnevich**: investigation. **Elisa Teyssou**: investigation. **Stéphane Marot**: investigation, methodology. **Adélie Gothland**: investigation. **Jean‐Michel Guigner**: visualization. **Anne‐Geneviève Marcelin**: supervision. **Mickaël Ménand**: conceptualization, writing – original draft, writing – review and editing, supervision, validation. **Vincent Calvez**: conceptualization, supervision, validation. **Matthieu Sollogoub**: conceptualization, funding acquisition, writing – original draft, writing – review and editing, validation, supervision, project administration.

## Conflicts of Interest

The authors declare no conflicts of interest.

## Supporting information




**Supporting File**: anie73621‐sup‐0001‐SuppMat.pdf.

## Data Availability

The data that support the findings of this study are available on request from the corresponding author. The data are not publicly available due to privacy or ethical restrictions.
