# Supplementary material for: Virus‐Mediated Self‐Assembly of Functional Cyclodextrins for Antiviral Inhibition
Source: Angew Chem Int Ed Engl. 2026 Jul 15;65(38):e3935543. doi: 10.1002/anie.3935543 (PMC13573067; doi:10.1002/anie.3935543)
Supplement: Supplementary file 1 [file ANIE-65-e3935543-s001.pdf]

Virus-Mediated Self-Assembly of Functional Cyclodextrins for Antiviral Inhibition

Pedro J. Hernando,[a†] Laora Boulo,[a†] Léonid Lavnevich,[a] Elisa Teyssou,[b] Adélie Gothland,[b] Jean-Michel Guigner,[c] Anne-Geneviève Marcelin,[b] Mickaël Ménand,[a*] Vincent Calvez,[b*] Matthieu Sollogoub[a,d*]

[a] *Sorbonne Université, CNRS, Institut Parisien de Chimie Moléculaire, IPCM, F-75005 Paris, France*
[b] *Sorbonne Université, Department of Virology, INSERM, AP-HP, Pitié-Salpêtrière Hospital, F-75013 Paris, France*
[c] *Sorbonne Université, CNRS, MNHN, Institut de minéralogie, de physique des matériaux et de cosmochimie, IMPMC, F-75005 Paris, France*
[d] *Institut Universitaire de France*

Table of contents

SI.1 Materials and instrumental techniques 2
SI.2 Synthesis and descriptions 3
SI.2.1 General methods 3
SI.2.2 Cyclodextrin synthesis 4
SI.2.3 Carbohydrate synthesis 20
SI.3 Copper testing data 26
SI.4 DOSY NMR 27
SI.5 Cytopathic effect and cytotoxicity assay 29
SI.6 Cryo-EM analysis 31

[†] These authors contributed equally to the work.

SI.1 Materials and instrumental techniques

Reactants were purchased from commercial sources and used without further purification. The **solvents** were freshly distilled by standard methods: Dichloromethane from P_2O_5; THF from sodium/benzophenone; toluene from; DMF, DMSO and triethylamine were dried over molecular sieves 3 Å. Materials for enzymatic chemistry (PmST1, PdST6, NmCSS, PmPPA, CTP) were purchased from Chemily Glycoscience.

Thin Layer Chromatography (TLC) was performed on Merck silica gel 60 F 254 and revealed with a UV lamp (λ = 254 nm) and $KMnO_4$, cerium molybdate or sulphuric acid staining.

Flash Column Chromatography was conducted on silica Geduran® Si 60 Å (40 – 63 μm) or silica Geduran® Si 60 Å (63 – 200 μm).

Size Exclusion Chromatography (SEC) was performed on a 80 cm-long, 3 cm-wide glass column packed with BioRad P2 gel, working at a 0.6 mL/min flow (by gravity). Reaction crudes were dissolved in 1 mL of distilled water, loaded in the column, and further rinsed with 1 mL of distilled water. Fractions of *ca*. 1 mL were collected with a Pharmacia Biotech FRAC-100 Fraction collector, checked by TLC [^{i}PrOH/NH_3 (aq. 30%)/H_2O, 6:3:1] and accordingly combined and freeze dried to afford the desired compounds.

High-Resolution Mass Spectrometry (HRMS) were recorded on a Bruker micrOTOF spectrometer, using Agilent ESI-L Low Concentration Tuning-Mix as reference.

Nuclear Magnetic Resonance (NMR) spectra were recorded on a Bruker Avance II 600 MHz or Bruker AM-400 MHz using residual solvent signal as internal reference. Assignments of the signals were done using Heteronuclear Single Quantum Coherence spectroscopy (HSQC), Heteronuclear Multiple Bound Correlation (HMBC), COrrelation SpectroscopY (COSY), TOtal Correlation SpectroscopY (TOCSY), Nuclear Overhauser Effect SpectroscopY (NOESY), Transverse Rotating-frame Overhauser Enhancement SpectroscopY (T-ROESY). All sugar units were renamed from unit A to unit G in the counterclockwise (first rim). Chemical shifts (δ) were reported in ppm and coupling constants (J) were given in Hertz (Hz). Abbreviations used for peak multiplicity were: app. (apparent) br. (broad signal); s (singlet); d (doublet); t (triplet); q (quartet); quint (quintet); sept (septet); m (multiplet).

Diffusion-Ordered Spectroscopy (DOSY) **NMR** diffusion experiment was performed by using the using bipolar longitudinal eddy current delay with gradients (LEDBPGP) sequence, spectra were acquired with gradient pulses (δ) of 2 ms ranging in strength from 0.28 to 5.26 g/mm for the BBFO 5mm NMR probe. A diffusion delay from 50 to 200 ms was set and diffusion coefficients (D) were calculated from mono-exponential decays using Bruker Topspin 3.0 software.

UV-Vis spectrometry was performed using a plate reader (Thermo Scientific Varioskan Lux) with a 96-well plate, exporting the data for analysis to Microsoft Excel.

Widefield inverted microscopy was performed using a EVOS XL AMEX1000 digital microscope, in brightfield mode, with a 4x lens.

SI.2 Synthesis and descriptions

SI.2.1 General methods

General method A: copper-catalysed azide-alkyne cycloaddition (CuAAC)

Adapted from Hernando *et al.*[1] Nitrogen was bubbled through distillated water prior to use for 30 minutes. The solvent was then employed to prepare solutions of the azide-containing molecule (1 eq., 20 mM), the alkyne counterpart (2.5 eq., 50 mM), $CuSO_4{\cdot}(H_2O)_5$ (2 eq., 800 mM), THPTA (5 eq., 800 mM) and NaAsc (5 eq., 800 mM). Separately, the azide and alkyne were mixed in a reaction flask, as well as $CuSO_4{\cdot}(H_2O)_5$ and THPTA, observing a change in color to dark blue for the latter. Then, both solutions were mixed and the NaAsc solution was added, observing a change in color to pale yellow. The flask was flushed with Argon and left stirring at room temperature overnight under Argon atmosphere. The reaction was monitored by ESI-TOF until the peak corresponding to the starting material (CD) was no longer detectable, at which point dry loading was prepared directly from the crude mixture and loaded into a short silica plug, which was washed with 5 CV of $^{i}PrOH/NH_3$ (aq. 32%), 8:2 and eluted with 5 CV of $^{i}PrOH/NH_3$ (aq. 32%)/H_2O, 6:3:1. The elution fractions were combined, concentrated under reduced pressure and finally freeze-dried to obtain the desired functionalised cyclodextrin as a white fluffy solid.

General method B: quantification of copper traces

Adapted from Ebbesen *et al.*[2] In a 96-well plate, a calibration curve for $CuSO_4{\cdot}(H_2O)_5$ was prepared in triplicate (3 × 8 points, 75 µL, 0 to 4 µM from stock solutions of 4 and 8 µM). Next, diethyldithiocarbamate (DDC, 75 µL, 20 µM) was added to each point to achieve a final Cu concentration between 0 and 2 µM and a final DDC concentration of 10 µM. Stock solutions of the samples to be tested were prepared (ca. 25 mg/mL in water) and each solution was tested in triplicate (1 µL of stock solution diluted in 74 µL of water and 75 µL of 20 µM DDC). The plate was placed in a plate reader and the absorbance was measured at 450 nm.

General method C: enzymatic *trans*-sialylation

Adapted from the method reported by Yu *et al.*[3] Stock solutions of acceptor (500 mM in distilled water), *N*-acetylneuraminic acid (500 mM in distilled water), cytidine triphosphate (CTP, 1 M in distilled water), $MgCl_2$ (1 M in distilled water) and Tris-HCl buffer (1 M, pH 8.6 at room temperature) were prepared. Pre-aliquoted stocks of transialidase (PmST1 or Pd26ST, 0.2 U at 1 U/mL in distilled water), cytidine monophosphate sialic acid synthetase (NmCSS, 0.2 U at 1 U/mL in distilled water) and alkaline phosphatase (PmPPA, 50 U at 850 U/mL in distilled water) were defrosted. In an Eppendorf, 100 µL of distilled water, 150 µL of Tris-HCl buffer (final concentration of 100 mM), 7.5 µL of $MgCl_2$ (final concentration of 5 mM), 185 µL of acceptor (93 µmol, 1 eq), 370 µL of *N*-acetylneuraminic acid (186 µmol, 2 eq) and 231 µL of CTP (231 µmol, 2.5 eq) were mixed. At that point, pH was adjusted as needed (*ca.* 100 µL) with NaOH 2 M. With a pH around 8.6, 58 µL of PMPPA (50 U), 200 µL of CMP-sialic acid synthetase (0.2 U) and 200 µL of transialidase (0.2 U) were added. The mixture was incubated at 37 ^{o}C with shacking (900 rpm, 10 min), after which the pH was checked and corrected as needed with NaOH 1 M (*ca.* 10 µL). After completion (monitored by TLC – EtOAc/MeOH/H_2O/HOAc, 4:2:1:0.1), the mixture was transferred to a falcon tube, precipitated (EtOH, 10 mL) and centrifuged (4000 rpm, 10 minutes). The supernatant was removed, and the solid was dried under vacuum and purified by SEC to obtain the desired sialoglycoside as a white fluffy solid.

SI.2.2 Cyclodextrin synthesis

6A, 6D-Dideoxy-6A,6D-diamino-*N,N'*-butyl-*N*-(ethyladamantyl)-*N'*-(5-(((5-acetamido-3,5-dideoxy-D-glycero-α-D-galacto-2-nonulopyranosyl)-methyl)-1H-1,2,3-triazoly-4-yl)-pentyl)-β-cyclodextrin chlorohydrate (**CD2**)

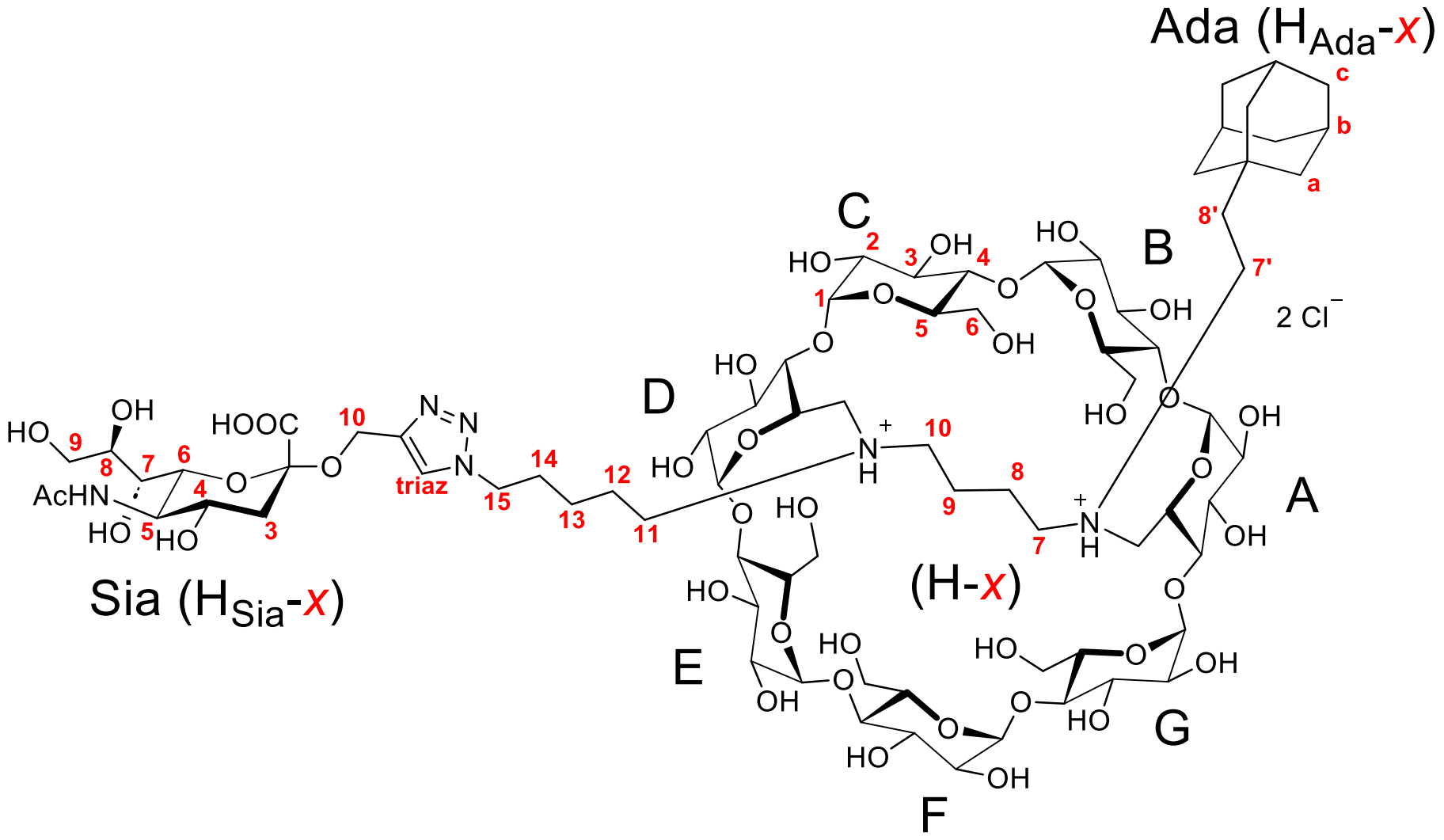

CD2 was obtained by following the *general method A* using **CD8** (76 mg, 0.05 mmol) and compound **10** (43 mg, 0.12 mmol) prepared according to Mony *et al*.[4] and Gan *et al*.[5], yielding **CD2** as a white fluffy solid (70 mg, 0.04 mmol, 75%). The residual trace of copper was quantified as 0.11%mol following the *general method B.*

$\mathbf{R_f}$ = 0.3 [^{i}PrOH/NH_3 (aq. 30%)/H_2O, 6:3:1].

^{1}H-NMR (600 MHz, D_2O, 300K): 8.05 (s, 1 H, H-triaz), 5.20-5.00 (m, 7 H, 7 x H-1), 4.90 (d, $J_{Sia10a,Sia10b}$ = 11.9 Hz, 1 H, H_{Sia}-10a), 4.63 (d, $J_{Sia10a,Sia10b}$ = 11.9 Hz, 1 H, H_{Sia}-10b), 4.55-4.45 (m, 2 H, H-15), 4.10-3.80 (m, 22 H, 7 x H-3, 14 x H-6, H_{Sia}-9a), 3.80-3.40 (m, 27 H, 7 x H-2, 7 x H-4, 7 x H-5, H_{Sia}-4, H_{Sia}-5, H_{Sia}-6, H_{Sia}-7, H_{Sia}-8, H_{Sia}-9b), 3.40-2.80 (br., 8 H, H-7, H-7', H-10, H-11), 2.78 (dd, $J_{Sia3ax,Sia3eq}$ = 10.6 Hz, $J_{Sia3eq,Sia4}$ = 4.6 Hz, 1 H, H_{Sia}-3$_{eq}$), 2.10 (br. s, 3 H, H_{Ada}-b), 2.06 (s, 3 H, H_{Sia}-*N*Ac), 2.00-1.93 (m, 2 H, H-14), 1.90-1.40 (m, H-8, H-8', H-9, H-12, 6 x H_{Ada}-a, 6 x H_{Ada}-c, H_{Sia}-3$_{ax}$), 1.35-1.24 (m, 2 H, H-13).

^{13}C-NMR (150 MHz, D_2O, 300K) was assigned by correlation with ^{1}H,^{13}C-HSQC: 175.1 (Sia-COOH), 173.2 (C_{Sia}-NH*C*(O)CH$_3$), 125.0 (C-triaz), 103.0-101.5 (m, 7 x C-1), 100.6 (C_{Sia}-2), 74.0-70.6 (m, 7 x C-2, 7 x C-3, 7 x C-4, 7 x C-5, C_{Sia}-6,C_{Sia}-7, C_{Sia}-8), 68.1 (C_{Sia}-4), 62.6 (C_{Sia}-9), 62.6-62.4 (m, 2 x C-7, C-7', C-11), 60.9-59.9 (m, 7 x C-6), 57.4 (s, C_{Sia}-10), 51.9 (C_{Sia}-5), 50.1 (C-15), 42.2-41.4 (m, C-8, C-8', C-9, C-12, C-13), 40.3 (C_{Sia}-3), 38.0-37.0 (3 x C_{Ada}-a, 3 x C_{Ada}-c, C-15), 29.0 (C-14), 28.5 (3 x C_{Ada}-b), 22.0 (C_{Sia}-NHC(O)*CH$_3$*).

HR-MS (ESI-TOF) m/z: $[M + H]^+$; calcd for $C_{77}H_{127}N_6O_{42}$ 1807.7981; found: 1807.8096 (err: 3.2 ppm).

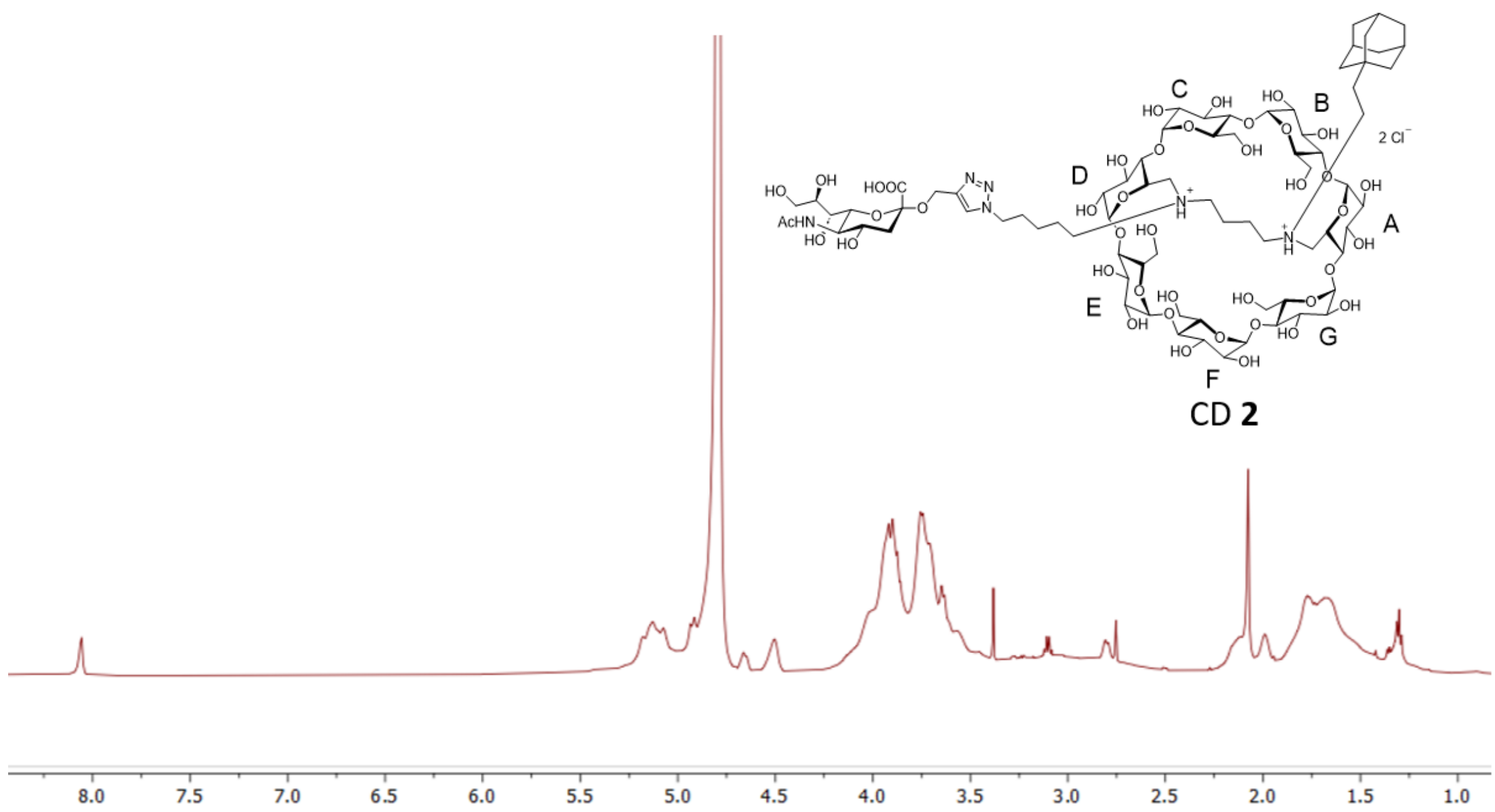

^{1}H-NMR (600 MHz, D2O, 300K) of **CD2**

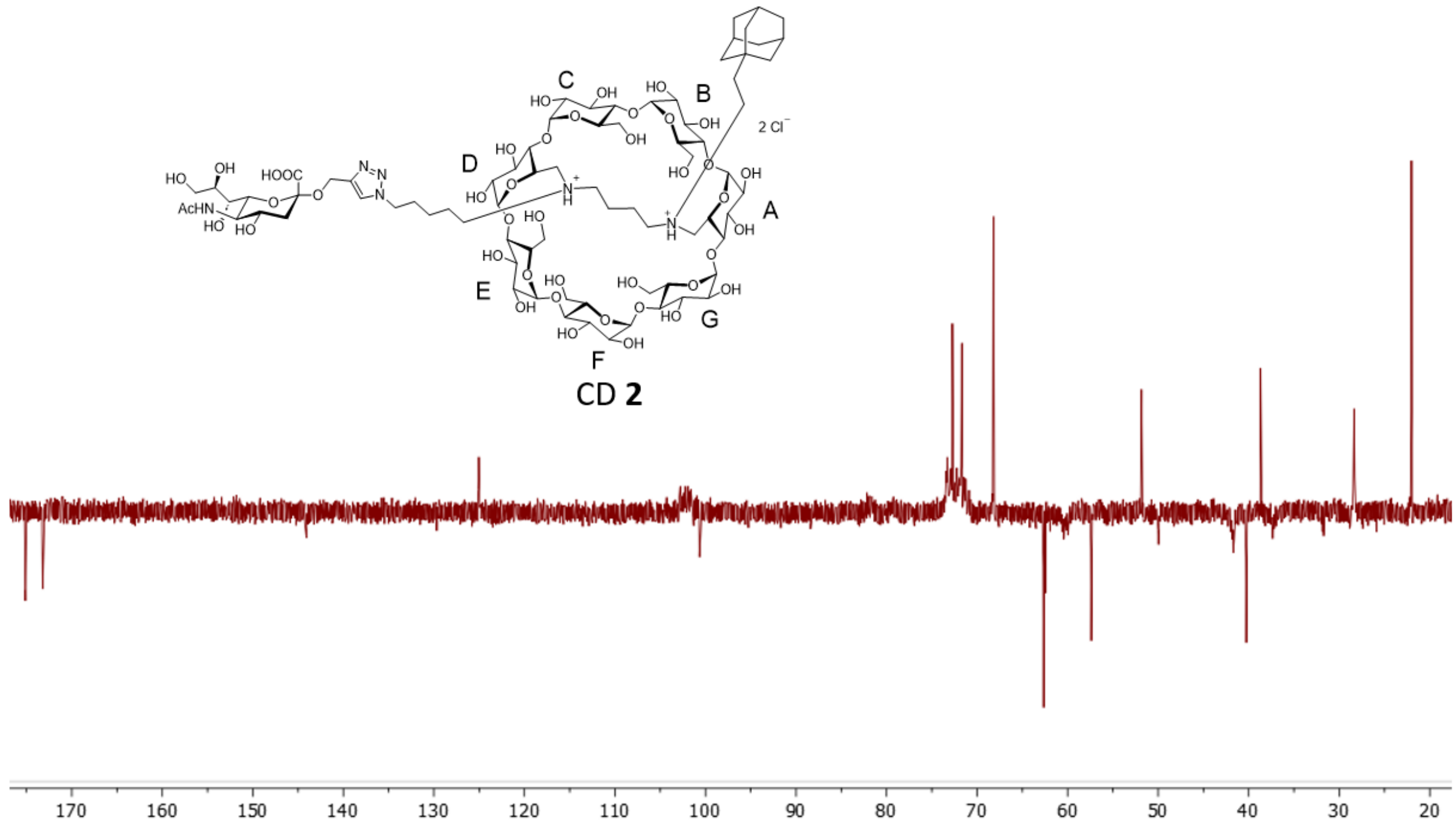

^{13}C-NMR (150 MHz, D_2O, 300K) of **CD2**

6^A, $6^{D/E}$-Dideoxy-6^A,$6^{D/E}$-diamino-*N,N'*-butyl-*N'*-(5-(((5-acetamido-3,5-dideoxy-D-glycero-α-D-galacto-2-nonulopyranosyl)-methyl)-1H-1,2,3-triazoly-4-yl)-pentyl)-β-cyclodextrin chlorohydrate (**CD3**)

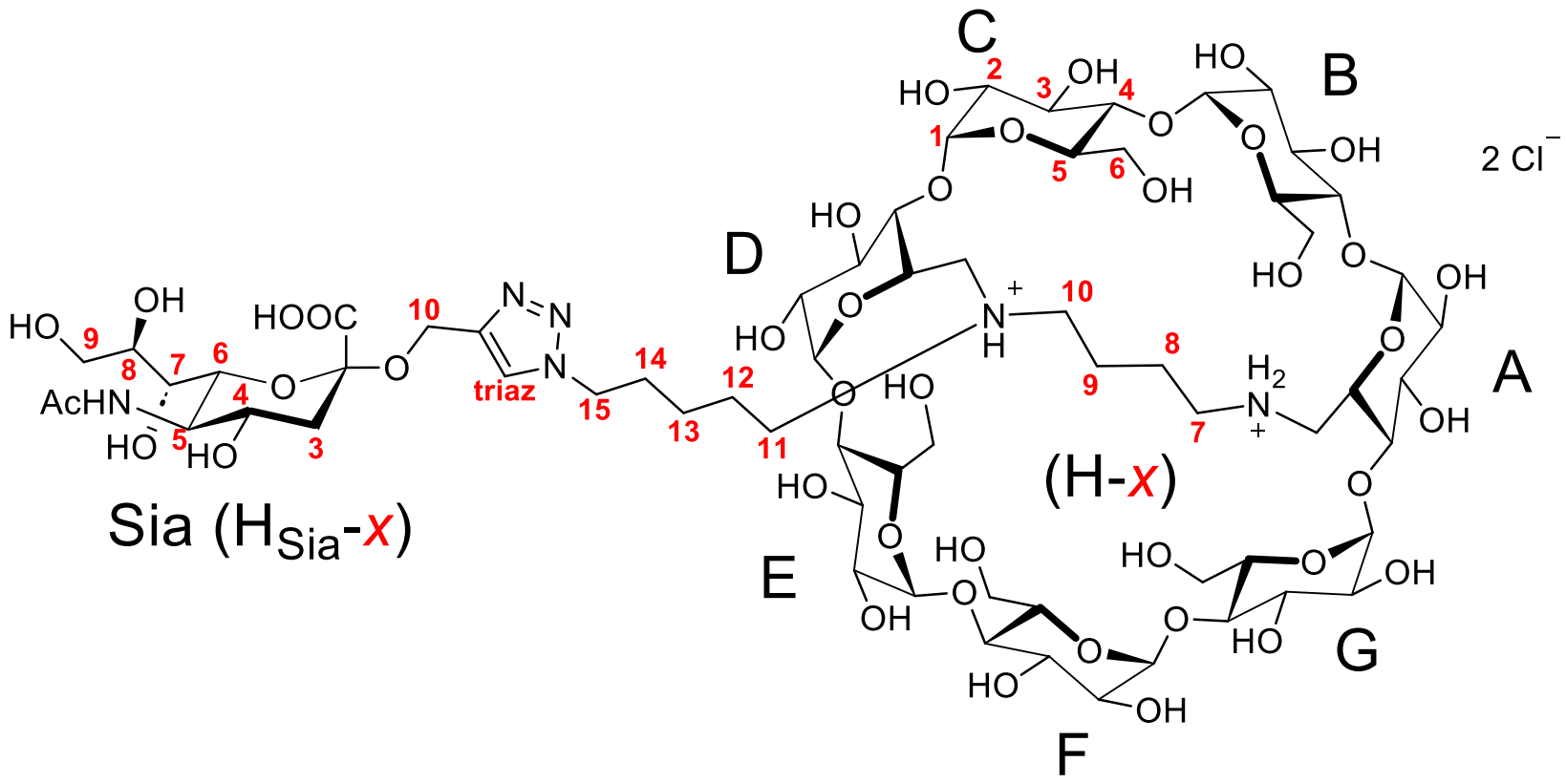

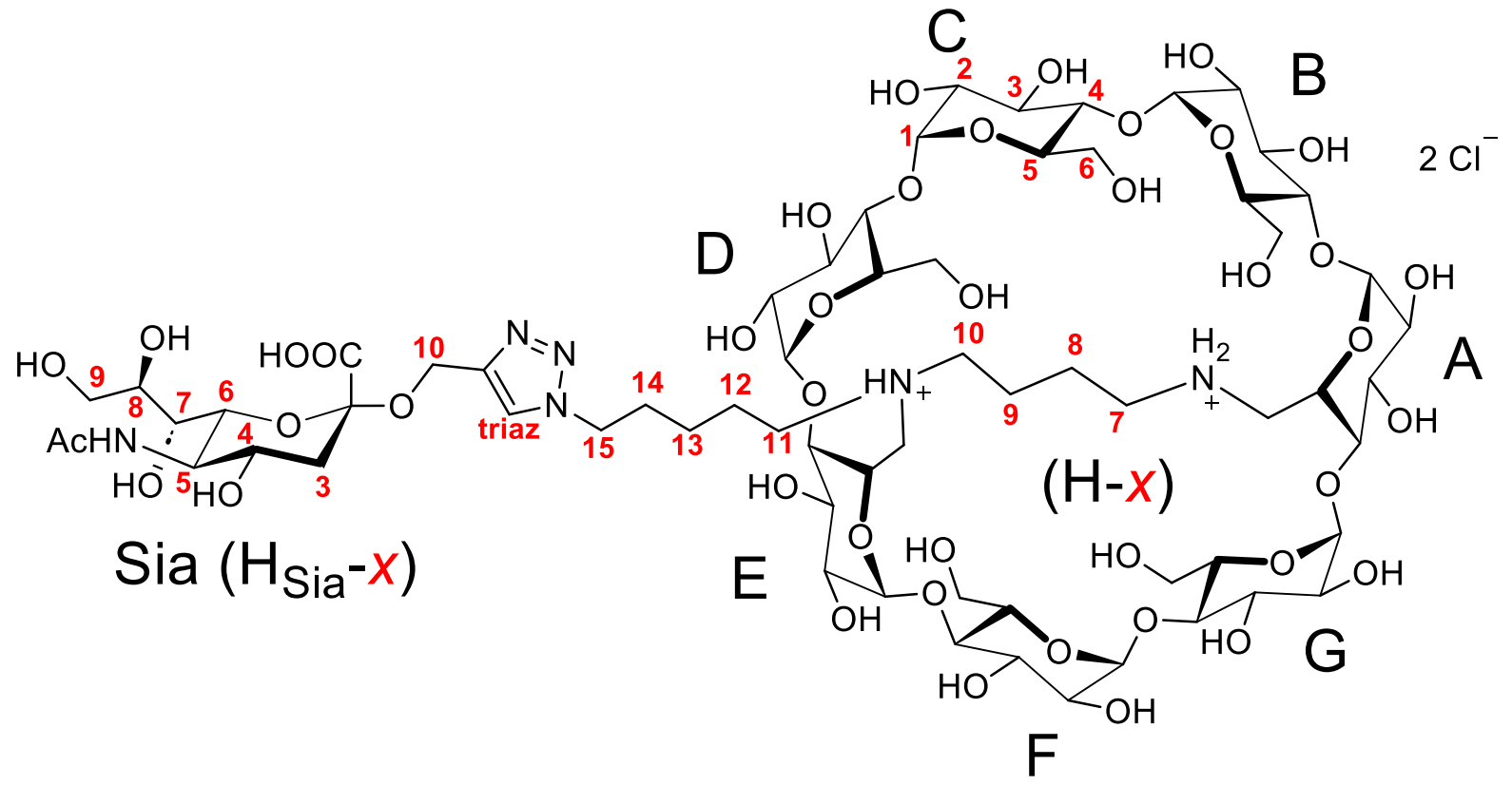

CD3 was obtained by following the *general method A* using **CD8b** (26 mg, 0.02 mmol) and compound **10** (16 mg, 0.05 mmol), yielding **CD3** as a white fluffy solid mixture of the AE and AD regioisomers (29 mg, 0.02 mmol, 87%).

R_f = 0.25 [^{i}PrOH/NH_3 (aq. 30%)/H_2O, 6:3:1].

^{1}H-NMR (400 MHz, D_2O, 300K): 8.05 (s, 1 H, H-triaz), 5.27 (d, $J_{1,2}$ = 3.8 Hz, 1H, $H_{D/E}$-1), 5.21-5.06 (m, 6 H, 6 x H-1), 4.93 (d, $J_{Sia10a,Sia10b}$ = 11.7 Hz, 1 H, H_{Sia}-10a), 4.68 (d, $J_{Sia10a,Sia10b}$ = 11.7 Hz, 1 H, *CH₂*-triaz), 4.50 (t, $J_{14,15}$ = 6.6 Hz, 2 H, H-15), 4.41-4.26 (m, 2 H, H_A-5, $H_{D/E}$-5), 4.10-3.82 (m, 22 H, 7 x H-3, 14 x H-6, H_{Sia}-9a), 3.82-3.60 (m, 27 H, 7 x H-2, 7 x H-4, 7 x H-5, H_{Sia}-4, H_{Sia}-5, H_{Sia}-6, H_{Sia}-7, H_{Sia}-8, H_{Sia}-9b), 3.40-2.80 (br., 6 H, H-7, H-10, H-11), 2.78 (dd, $J_{Sia3ax,Sia3eq}$ = 12.5 Hz, $J_{Sia3eq,Sia4}$ = 4.7 Hz, 1 H, H_{Sia}-3_{eq}), 2.07 (s, 3 H, H_{Sia}-*N*Ac), 1.99 (m, 2 H, H-14), 1.91-1.67 (m, H-8, H-9, H-12, H_{Sia}-3_{ax}), 1.38-1.27 (m, 2 H, H-13).

^{13}C-NMR (150 MHz, D_2O, 300K) was assigned by correlation with ^{1}H,^{13}C-HSQC: 175.1 (Sia-COOH), 173.2 (C_{Sia}-NH*C*(O)CH_3), 125.1 (C-triaz), 102.7-98.9 (m, 7 x C-1, C_{Sia}-2), 84-78 (m, H_{Sia}-6, H_{Sia}-7, H_{Sia}-8), 74.1-70.2 (m, 7 x C-2, 7 x C-3, 7 x C-4, 7 x C-5, C_{Sia}-4, C_{Sia}-6, C_{Sia}-7), 63.0-62.1 (C_{Sia}-9), 61.6-59.4 (m, 7 x C-6), 57.4 (s, C-10), 56.5-53 (m, C-7, C-10, C-11), 51.9 (C_{Sia}-5), 49.9 (C-15), 40.2 (C_{Sia}-3), 28.6 (C-14), 24-22 (m, C-8, C-9, C-12, C-13), 22.0 (C_{Sia}-NHC(O)*CH_3*).

HR-MS (ESI-TOF) m/z: [M + 2 H]$^+$; calcd for $C_{65}H_{110}N_6O_{42}$ 823.3323; found: 823.3331 (err: 1.0 ppm).

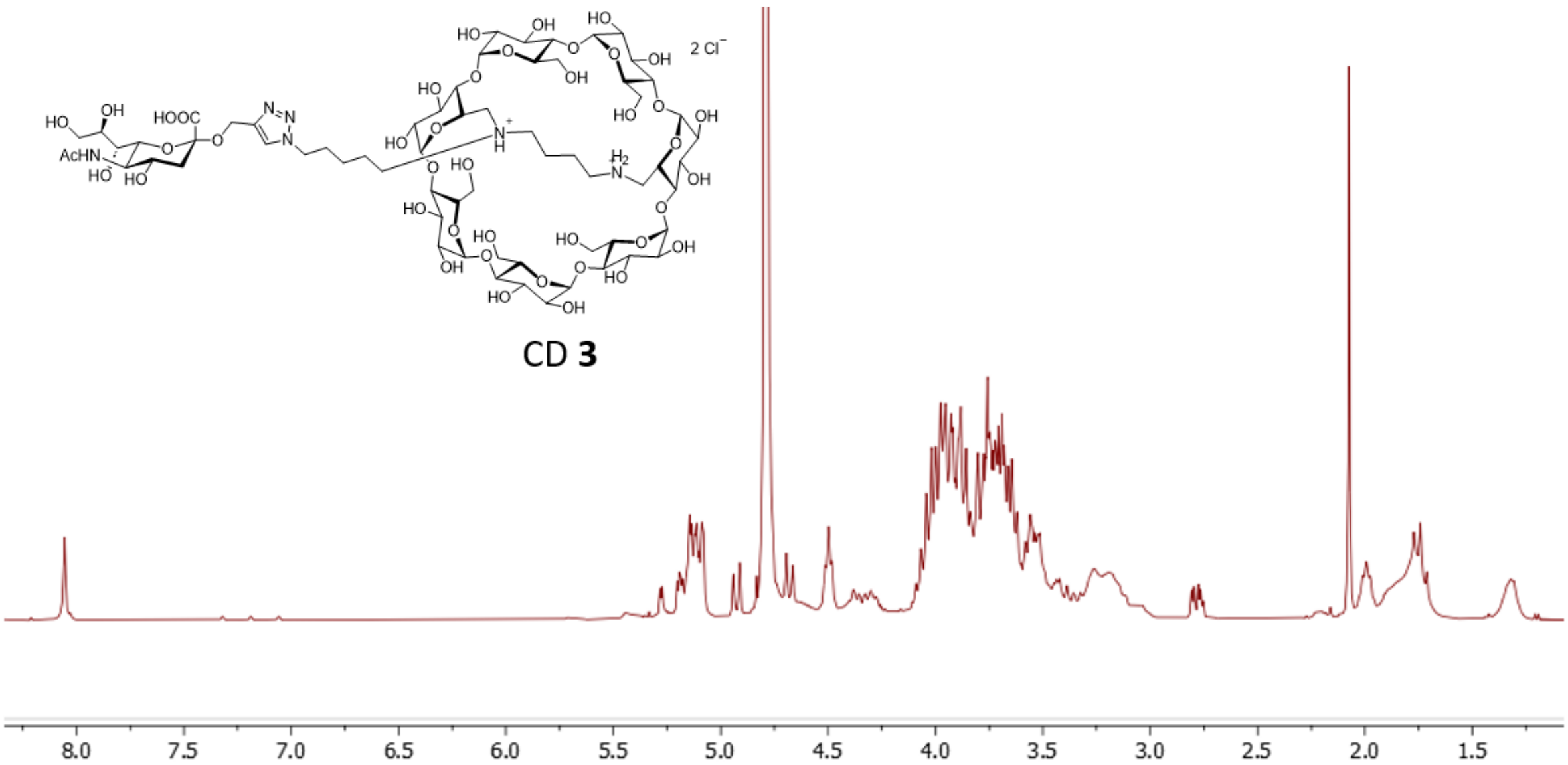

^{1}H-NMR (600 MHz, D_2O, 300K) of **CD3**

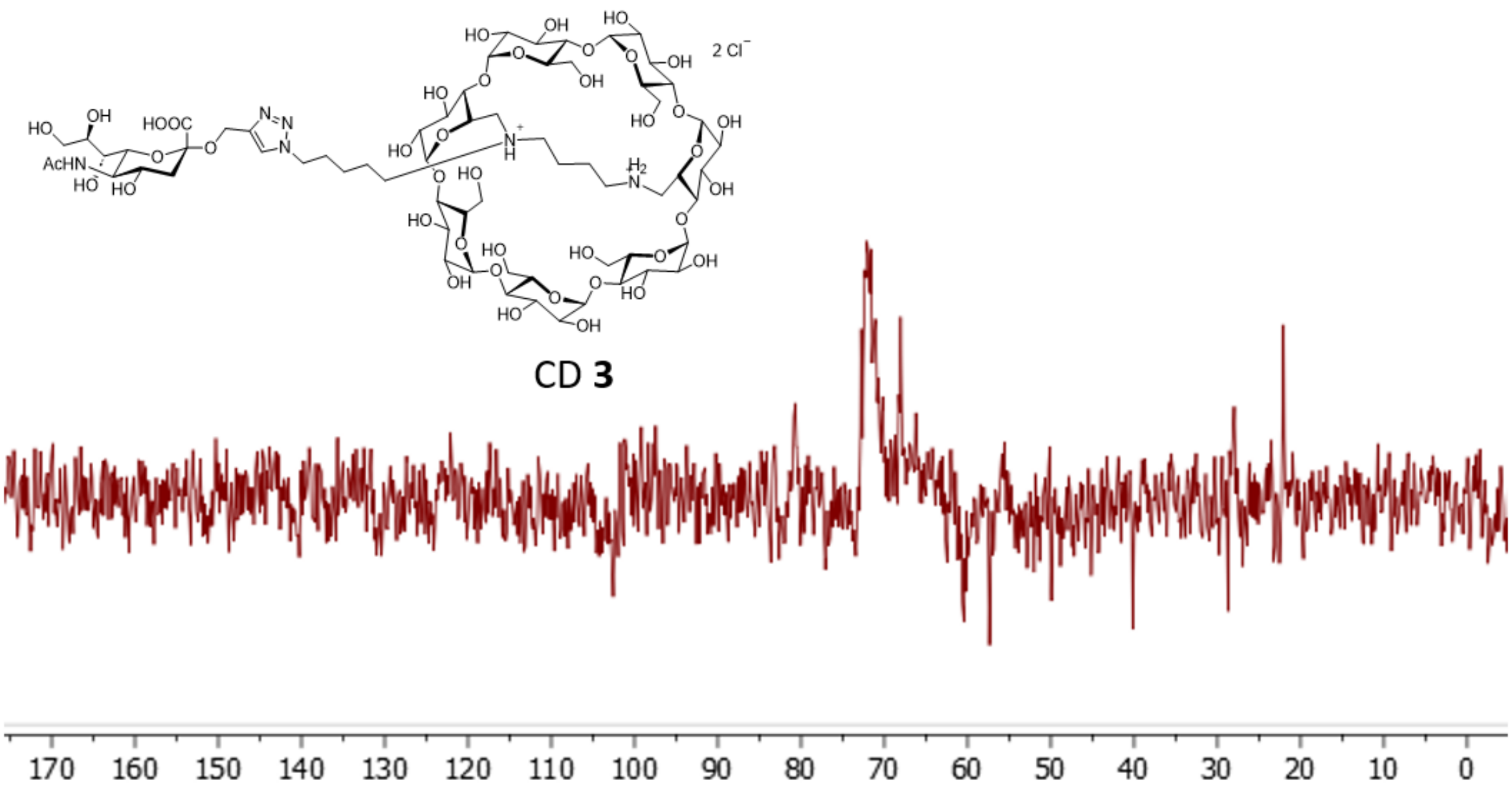

^{13}C-NMR (150 MHz, D_2O, 300K) of **CD3**

6^A, 6^D-Dideoxy-6^A,6^D-diamino-*N,N'*-butyl-*N*-(ethyladamantyl)-*N'*-(5-(((5-acetamido-9-*O*-acetyl-3,5-dideoxy-D-glycero-α-D-galacto-2-nonulopyranosyl)-methyl)-1H-1,2,3-triazoly-4-yl)-pentyl)-β-cyclodextrin chlorohydrate (**CD4**)

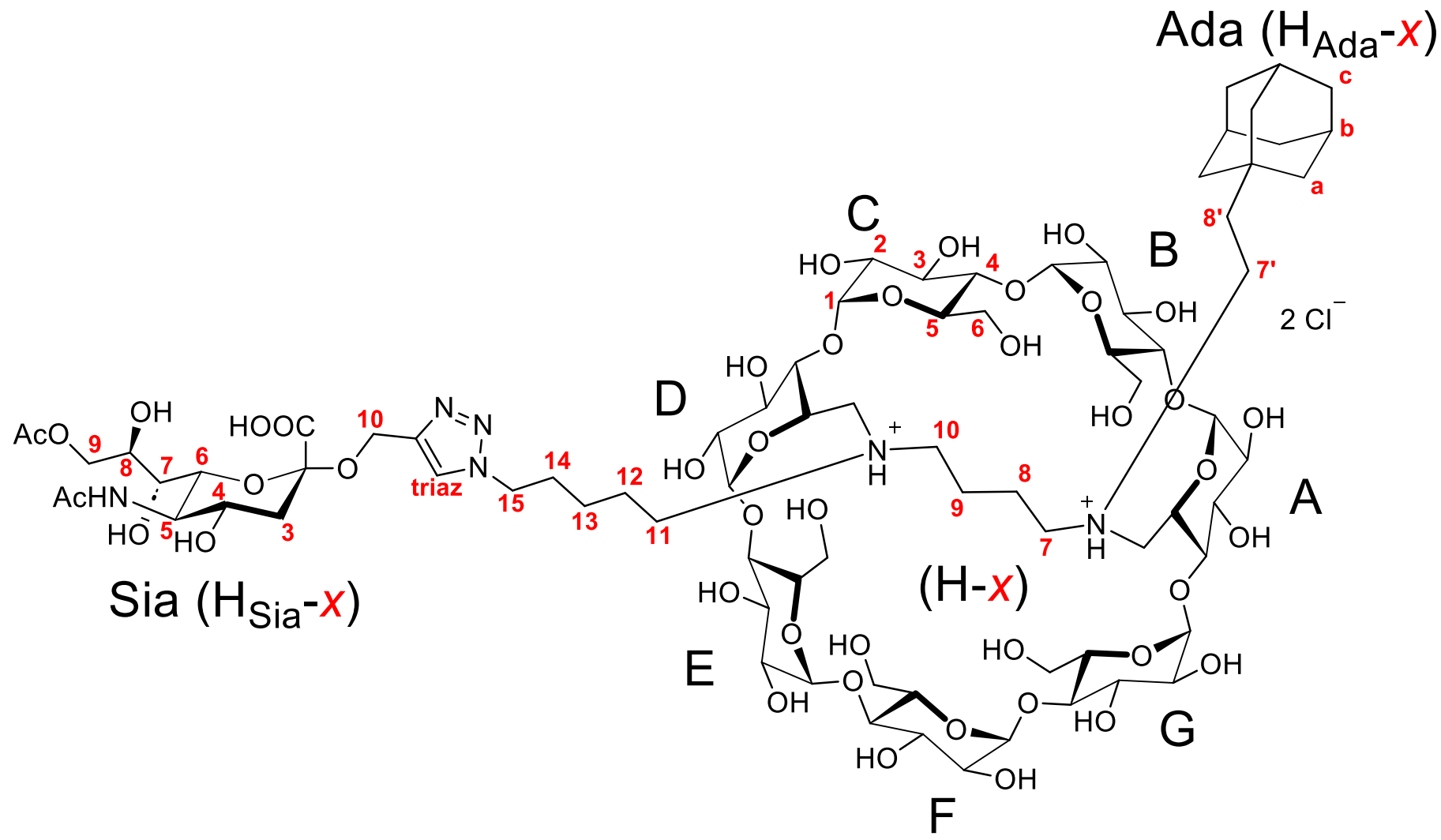

CD4 was obtained by following the *general method A* using CD **8** (30 mg, 0.02 mmol) and compound **11** (16 mg, 0.04 mmol), yielding **CD4** as a white fluffy solid (25 mg, 0.01 mmol, 70%). The remaining trace of copper was quantified as 0.56%mol following the *general method B.*

$\mathbf{R_f}$ = 0.30 [^{i}PrOH/NH_3 (aq. 30%)/H_2O, 6:3:1).

^{1}H-NMR (600 MHz, D_2O, 300K): 8.03 (s, 1 H, H-triaz), 5.17-4.96 (m, 7 H, 7 x H-1), 4.90 (d, $J_{Sia10a,Sia10b}$ = 11.6 Hz, 1 H, H_{Sia}-10a), 4.64 (d, $J_{Sia10a,Sia10b}$ = 11.6 Hz, 1 H, H_{Sia}-10b), 4.42 (d, $J_{SiaH9a,SiaH9b}$ = 11.5 Hz, 1 H, H-9a), 4.25-4.18 (m, 3 H, H-15, H_{Sia}-9b), 4.10-3.79 (m, 22 H, 7 x H-3, 14 x H-6), 3.79-3.62 (m, 27 H, 7 x H-2, 7 x H-4, 7 x H-5, H_{Sia}-4, H_{Sia}-5, H_{Sia}-6, H_{Sia}-7, H_{Sia}-8), 3.61-3.33 (br., 8 H, H-7, H-7', H-10, H-11), 2.77 (app. d, $J_{Sia3ax,Sia3eq}$ = 9.6 Hz, 1 H, H_{Sia}-3_{eq}), 2.15 (s, 3 H, *O*Ac), 2.10 (br. s, 3 H, H_{Ada}-b), 2.06 (s, 3 H, *N*Ac), 2.00-1.93 (m, 2 H, H-14), 1.84-1.42 (m, H-8, H-8', H-9, H-12, 6 x H_{Ada}-a, 6 x H_{Ada}-c, H_{Sia}-3_{ax}), 1.34-1.24 (m, 2 H, H-13).

^{13}C-NMR (150 MHz, D_2O, 300K) was assigned by correlation with ^{1}H,^{13}C-HSQC: 175.1 (Sia-COOH), 174.3 (C_{Sia}-*C*(O)OCH_3), 173.2 (C_{Sia}-NH*C*(O)CH_3), 125.0 (C-triaz), 103.3-99.4 (m, 7 x C-1, C_{Sia}-2), 73.9-67.6 (m, 7 x C-2, 7 x C-3, 7 x C-4, 7 x C-5, C_{Sia}-4, C_{Sia}-6,C_{Sia}-7, C_{Sia}-8), 66-65.4 (C_{Sia}-9), 61.9-58.1 (m, , 7 x C-6, C-7, C-7', C-10, C-11), 57.6 (s, C_{Sia}-10), 51.8 (C_{Sia}-5), 50.2 (s, C-15), 42.5-41.7 (m, C-8, C-8', C-9, C-12), 40.4 (C_{Sia}-3), 37.9-37.1 (m, C-15, 3 x C_{Ada}-a, 3 x C_{Ada}-c), 29.3 (C-14), 28.4 (3 x C_{Ada}-b), 23.5 (C-13), 22.0 (C_{Sia}-NHC(O)*CH_3*), 22.3 (C_{Sia}-C(O)O*CH_3*).

HR-MS (ESI-TOF) m/z: $[M + H]^+$; calcd for $C_{79}H_{130}N_6O_{43}$ 1850.8087; found: 1807.7988 (err: 4.1 ppm).

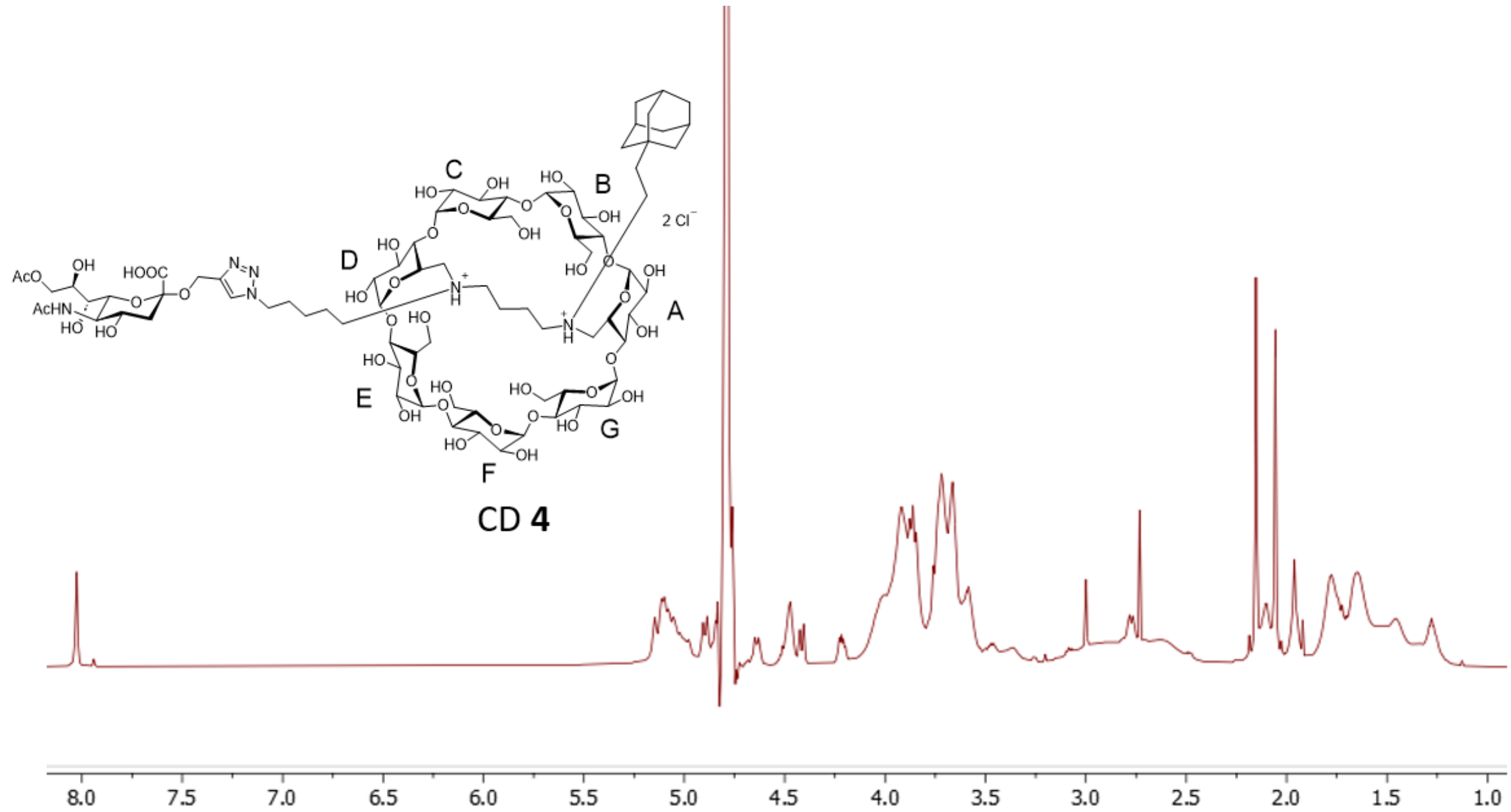

^{1}H-NMR (600 MHz, D_2O, 300K) of **CD4**

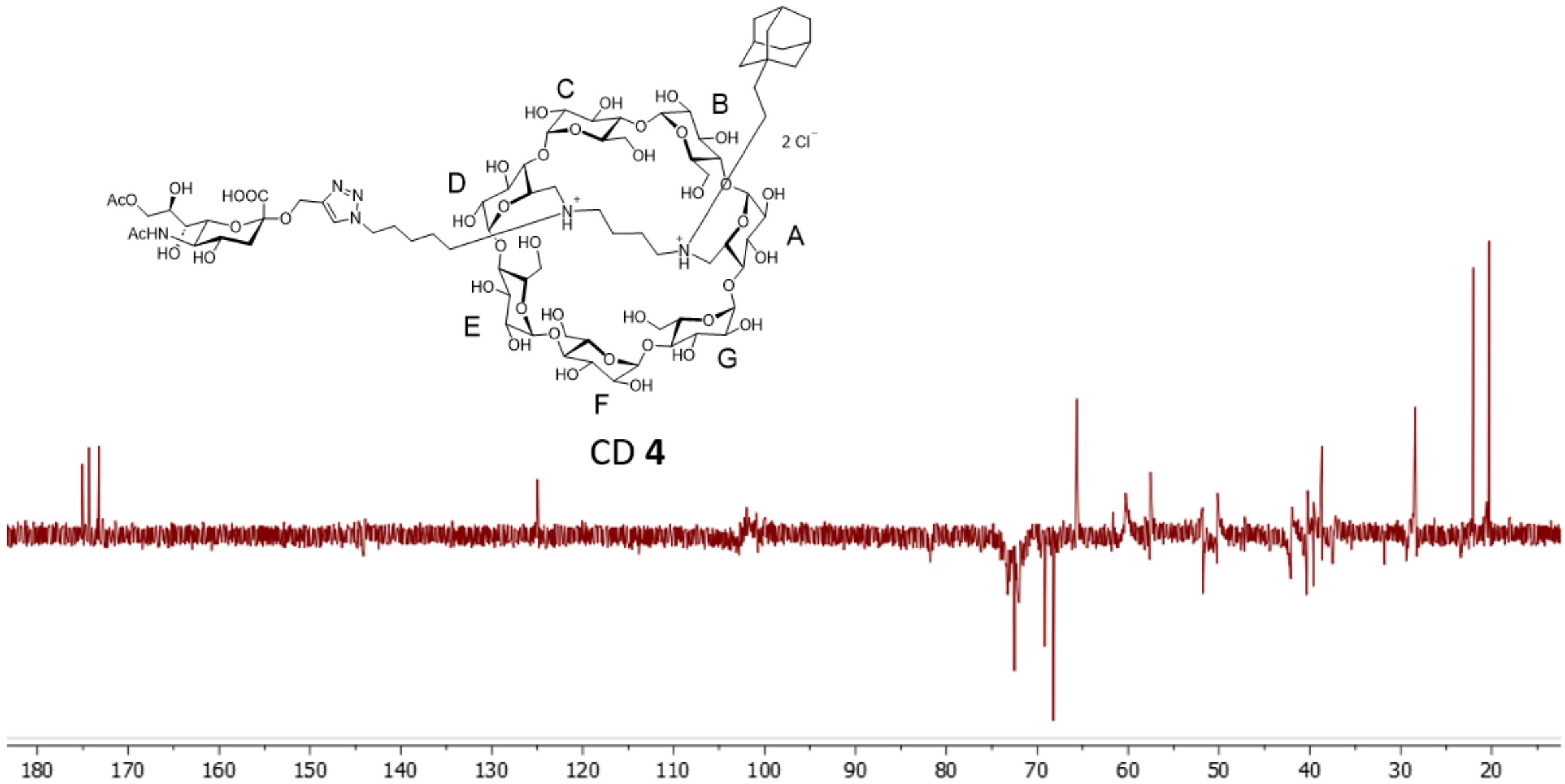

^{13}C-NMR (150 MHz, D_2O, 300K) of **CD4**

6^A, 6^D-Dideoxy-6^A,6^D-diamino-*N,N'*-butyl-*N*-(ethyladamantyl)-*N'*-(5-((4-*O*-(3-*O*-(5-acetamido-3,5-dideoxy-D-glycero-α-D-galacto-2-nonulopyranosyl)-β-D-galactopyronaoyl)-β-D-glucopyranoside)-methyl-1H-1,2,3-triazoly-4-yl)-pentyl)-β-cyclodextrin chlorohydrate (**CD5**)

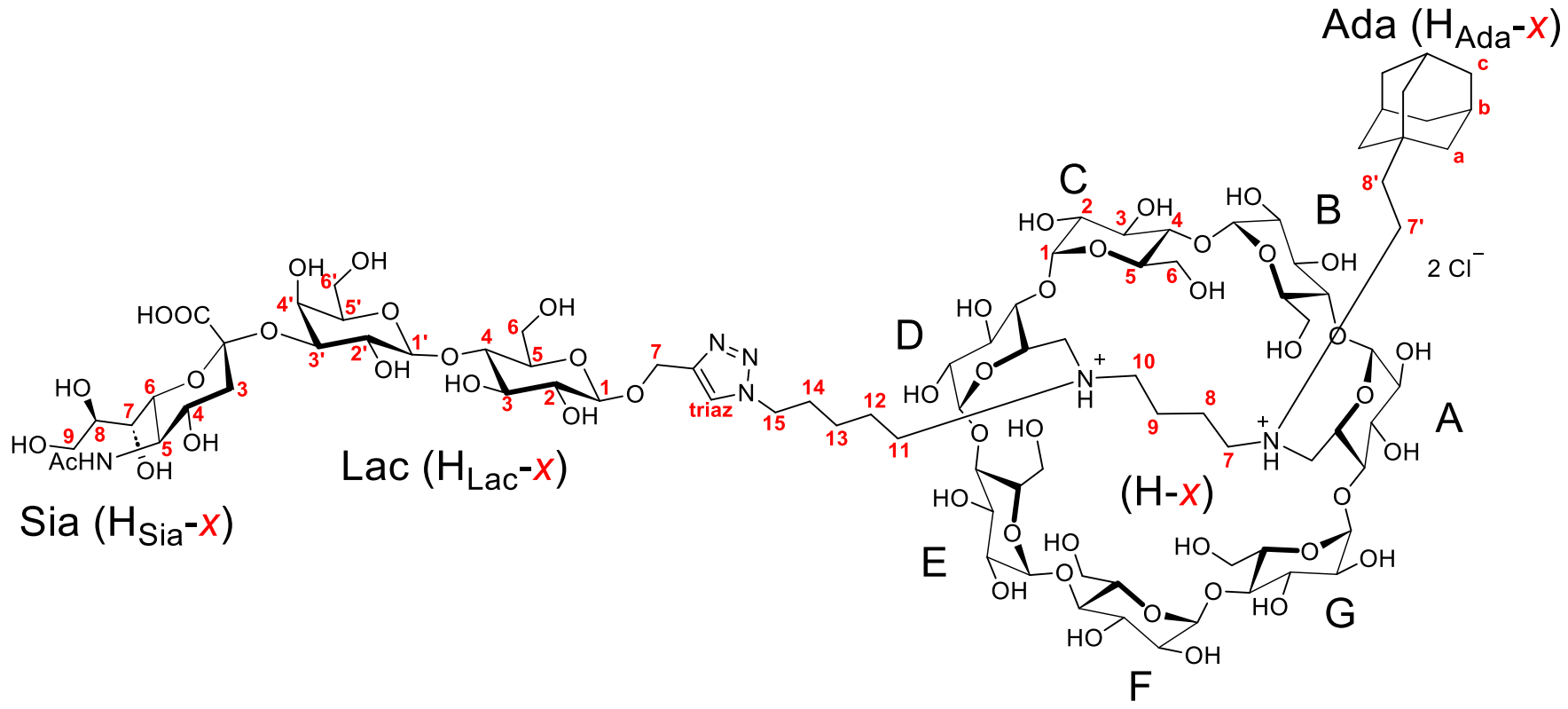

CD5 was obtained by following the *general method A* using **CD8** (30 mg, 0.02 mmol) and compound **13** (24 mg, 0.04 mmol), yielding **CD5** as a white fluffy solid (31 mg, 0.01 mmol, 73%). The remaining trace of copper was quantified as 0.56%mol following the *general method B.*

R$_f$ = 0.35 [^{i}PrOH/NH$_3$ (aq. 30%)/H$_2$O, 6:3:1].

^{1}H-NMR (600 MHz, D$_2$O, 300K): 8.10 (s, 1 H, H-triaz), 5.17-4.97 (m, 8 H, 7 x H-1, H$_{Lac}$-7a), 4.90 (d, $J_{Lac7a,Lac7b}$ = 12.2 Hz, 1 H, H$_{Lac}$-7b), 4.58 (d, $J_{Lac1',Lac2'}$ = 7.2 Hz, 1H, H$_{Lac}$-1'), 4.54 (d, $J_{Lac1, Lac2}$ = 7.7 Hz, 1H, H$_{Lac}$-1), 4.52-4.46 (m, 2 H, H-15), 4.12 (app. d, $J_{Lac2',Lac3'}$ = 9.1 Hz, 1H, H$_{Lac}$-3'), 4.04-3.81 (m, 28 H, 7 x H-3, 14 x H-6, H$_{Sia}$-9a, H$_{Lac}$-4, H$_{Lac}$-4', H$_{Lac}$-5, H$_{Lac}$-5', H$_{Lac}$-6a, H$_{Lac}$-6a'), 3.80-3.40 (m, 27 H, 7 x H-2, 7 x H-4, 7 x H-5, H$_{Sia}$-4, H$_{Sia}$-5, H$_{Sia}$-6, H$_{Sia}$-7, H$_{Sia}$-8, H$_{Sia}$-9b, H$_{Lac}$-2, H$_{Lac}$-6b, H$_{Lac}$-6,'), 3.36 (t, $J_{2',3'}$ = 9.1 Hz, 1H, H$_{Lac}$-2'), 3.60-3.43 (br., 8 H, H-7, H-7', H-10, H-11), 2.77 (app. dd, $J_{Sia3ax,Sia3eq}$ = 10.4 Hz, 1 H, H$_{Sia}$-3$_{eq}$), 2.08 (br. s, 3 H, H$_{Ada}$-b), 2.04 (s, 3 H, H$_{Sia}$-*N*Ac), 2.00-1.97 (br. s, 2 H, H-14), 1.83-1.57 (m, H-8, H-8', H-9, H-12, 6 x H$_{Ada}$-a, 6 x H$_{Ada}$-c, H$_{Sia}$-3$_{ax}$), 1.50-1.24 (m, 2 H, H-13).

^{13}C-NMR (150 MHz, D$_2$O, 300K) was assigned by correlation with ^{1}H,^{13}C-HSQC: 175.0 (Sia-COOH), 173.9 (C$_{Sia}$-NH*C*(O)CH$_3$), 125.2 (C-triaz), 103.0-101.6 (m, 7 x C-1), 102.7 (C$_{Lac}$-1), 101.3 (C$_{Lac}$-1'), 99.8 (C$_{Sia}$-2), 81.8, (C$_{Lac}$-2), 78.8-78.0 (m, 7 x H-4), 75.5 (C$_{Lac}$-3'), 74.5-70.5 (m, 7 x C-2, 7 x C-3, 7 x C-4, 7 x C-5, C$_{Sia}$-6,C$_{Sia}$-7, C$_{Sia}$-8), 75.1, 74.9, 74.3, 72.9, 72.7, (C$_{Lac}$-2'), 71.7, 69.3, 68.1 (C$_{Sia}$-4), 68, 67.5, 62.7 (C$_{Sia}$-9), 62.1 (s, C-7), 61.0 (C$_{Lac}$-6, C$_{Lac}$-6'), 60.7-59.6 (m, 7 x C-6), 51.9 (C$_{Sia}$-5), 50.1 (C-15), 62.6-62.4 (m, C-7, C-7', C-10, C-11), 42.5-41.6 (m, C-8, C-8', C-9, C-12, C-13), 39.6 (C$_{Sia}$-3), 38.0-37.0 (C-15, 3 x C$_{Ada}$-a, 3 x C$_{Ada}$-c), 29.2 (C-14), 28.5 (3 x C$_{Ada}$-b), 22.0 (C$_{Sia}$-NHC(O)*CH$_3$*).

HR-MS (ESI-TOF) m/z: [M + H]$^+$; calcd for $C_{89}H_{147}N_6O_{52}$ 2131.9037; found: 2131.9118 (err: 3.8 ppm).

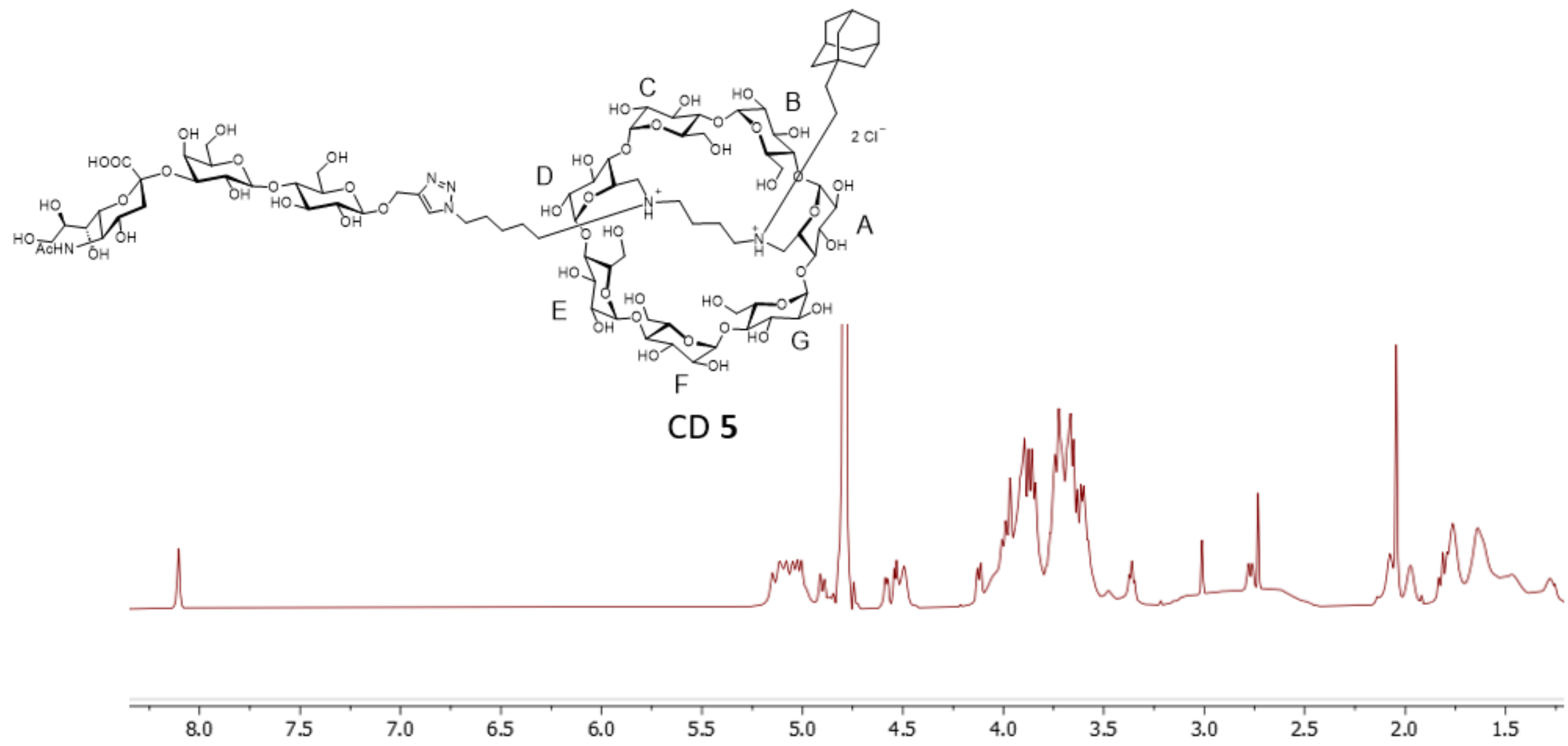

^{1}H-NMR (600 MHz, D_2O, 300K) of **CD5**

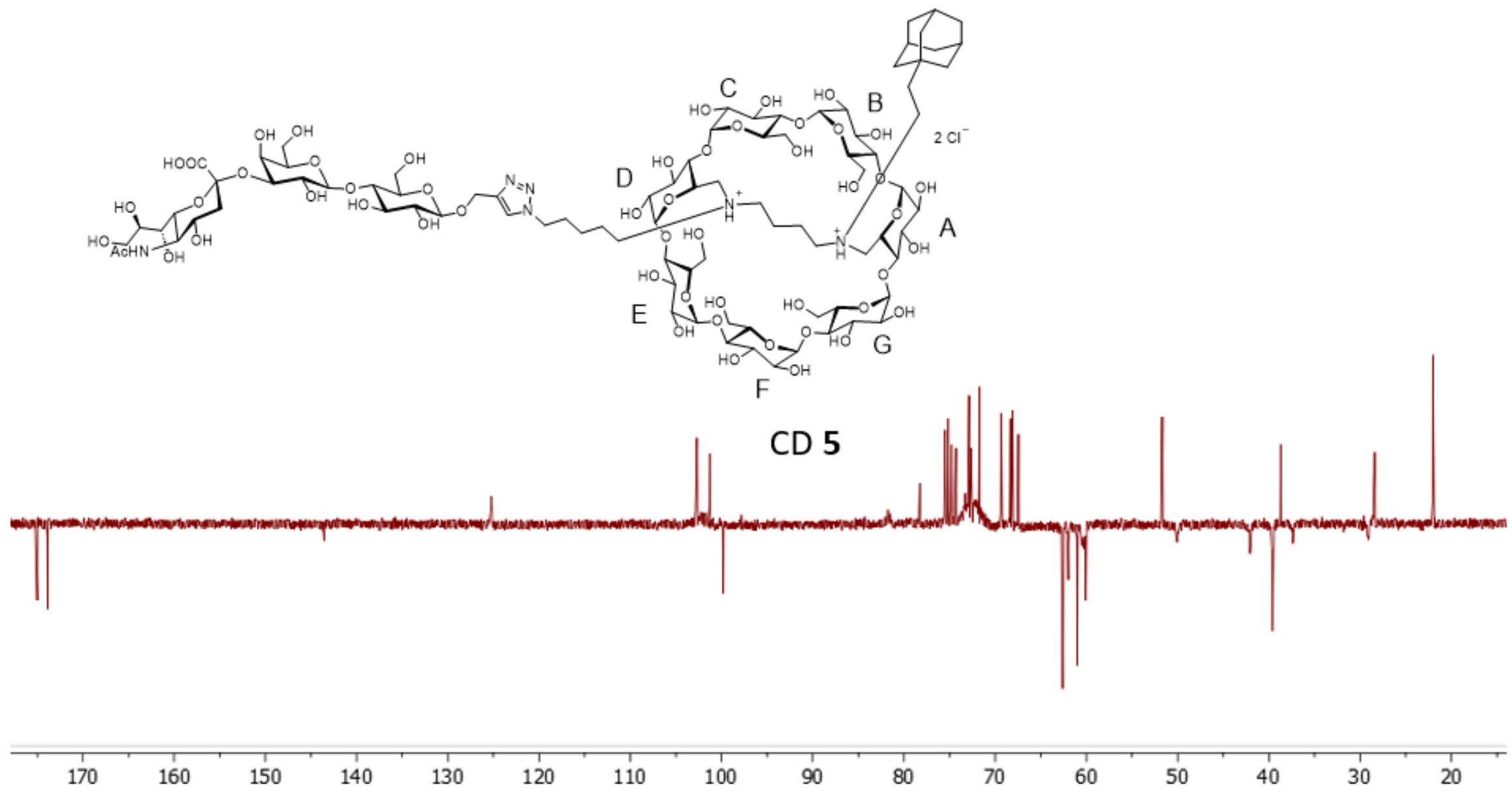

^{13}C-NMR (150 MHz, D_2O, 300K) of **CD5**

6^A, 6^D-Dideoxy-6^A,6^D-diamino-*N,N'*-butyl-*N*-(ethyladamantyl)-*N'*-(5-((4-*O*-(6-*O*-(5-acetamido-3,5-dideoxy-D-glycero-α-D-galacto-2-nonulopyranosyl)-β-D-galactopyronaoyl)-β-D-glucopyranoside)-methyl-1H-1,2,3-triazoly-4-yl)-pentyl)-β-cyclodextrin chlorohydrate (**CD6**)

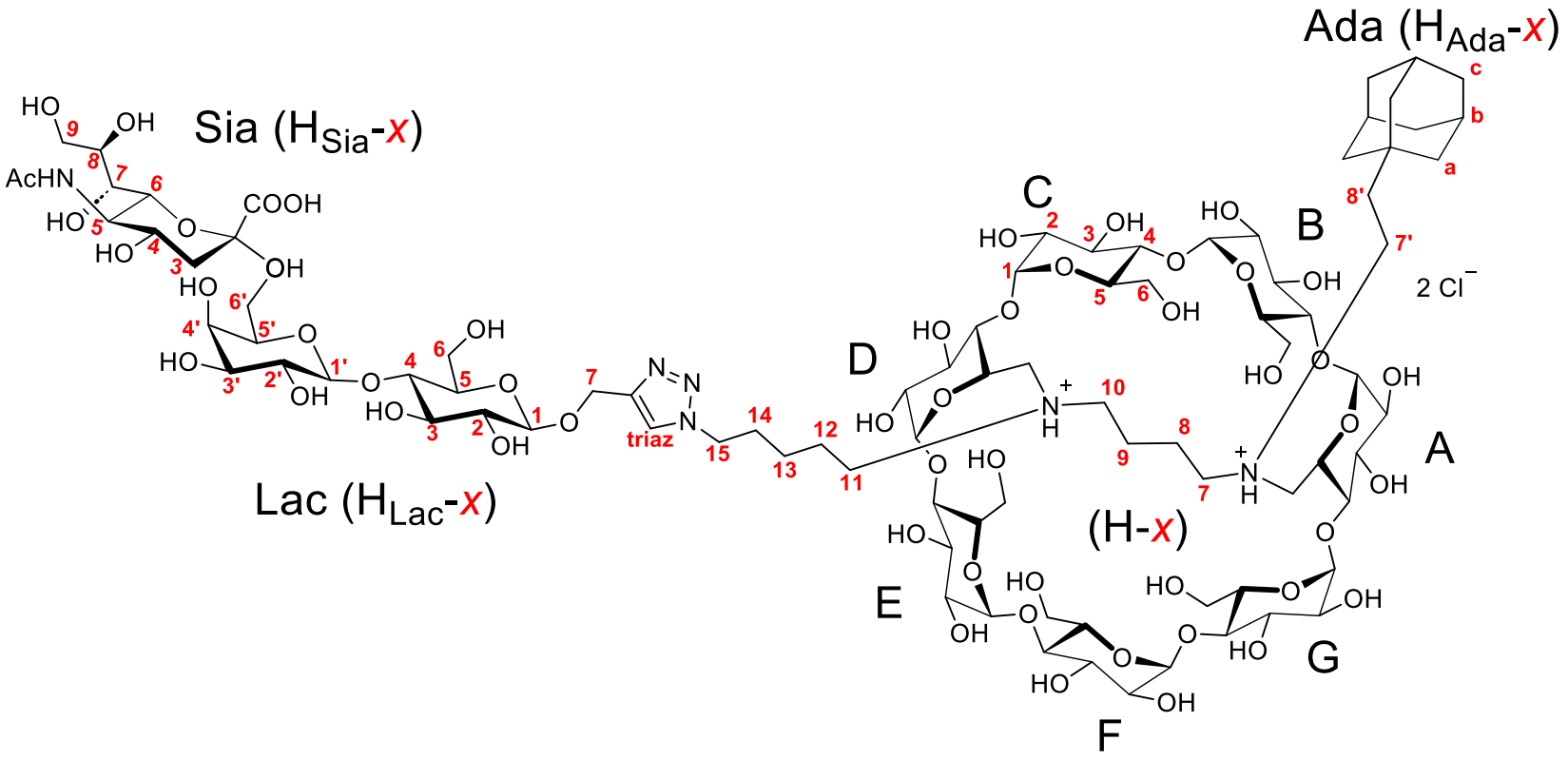

CD6 was obtained by following the *general method A* using CD **8** (30 mg, 0.02 mmol) and compound **14** (28 mg, 0.04 mmol), yielding **CD6** as a white fluffy solid (32 mg, 0.01 mmol, 78%). The remaining trace of copper was quantified as 0.78%mol following the *general method B.*

R$_f$ = 0.30 [^{i}PrOH/NH_3 (aq. 30%)/H_2O, 6:3:1].

^{1}H-NMR (600 MHz, D_2O, 300K): 8.10 (s, 1 H, H-triaz), 5.19-5.00 (m, 8 H, 7 x H-1, H$_{Lac}$-7a), 4.90 (d, $J_{Lac7a,Lac7b}$ = 12.4 Hz, 1 H, H$_{Lac}$-7b), 4.61 (d, $J_{1',2'}$ = 7.9 Hz, 1H, H$_{Lac}$-1'), 4.54-4.48 (m, 2 H, H-15), 4.44 (d, $J_{1,2}$ = 7.7 Hz, 1H, H$_{Lac}$-1), 4.04-3.79 (m, 28 H, 7 x H-3, 14 x H-6, H$_{Sia}$-9a, H$_{Lac}$-4, H$_{Lac}$-4', H$_{Lac}$-5, H$_{Lac}$-5', H$_{Lac}$-6a, H$_{Lac}$-6a'), 3.80-3.50 (m, 27 H, 7 x H-2, 7 x H-4, 7 x H-5, H$_{Sia}$-4, H$_{Sia}$-5, H$_{Sia}$-6, H$_{Sia}$-7, H$_{Sia}$-8, H$_{Sia}$-9b, H$_{Lac}$-2, H$_{Lac}$-6b, H$_{Lac}$-3', H$_{Lac}$-6,'), 3.38 (t, $J_{2',3'}$ = 8.2 Hz, 1H, H$_{Lac}$-2'), 3.60-3.43 (br., 8 H, H-7, H-7', H-10, H-11), 2.73-2.70 (m, 1 H, H$_{Sia}$-3$_{eq}$), 2.08 (br. s, 3 H, H$_{Ada}$-b), 2.05 (s, 3 H, H$_{Sia}$-*N*Ac), 1.98 (br. s, 2 H, H-14), 1.80-1.50 (m, H-8, H-8', H-9, H-12, 6 x H$_{Ada}$-a, 6 x H$_{Ada}$-c, H$_{Sia}$-3$_{ax}$), 1.50-1.24 (m, 2 H, H-13).

^{13}C-NMR (150 MHz, D_2O, 300K) was assigned by correlation with ^{1}H,^{13}C-HSQC: 174.9 (Sia-COOH), 173.5 (C$_{Sia}$-NH*C*(O)CH$_3$), 125.3 (C-triaz), 103.2 (C$_{Lac}$-1), 103.0-101.6 (m, 7 x C-1), 101.3 (C$_{Lac}$-1'), 100.3 (C$_{Sia}$-2), 79.8, (C$_{Lac}$-2), 75-74.4 (m, 7 x H-4), 73.8 (C$_{Lac}$-3'), 73.6-70.8 (m, 7 x C-2, 7 x C-3, 7 x C-4, 7 x C-5, C$_{Sia}$-4, C$_{Sia}$-6,C$_{Sia}$-7, C$_{Sia}$-8), 72.7(C$_{Lac}$-2'), 72.6, 72.4, 71.8, 70.8, 68.5, 67.5, 63.5 (C$_{Sia}$-9), 62.1 (s, C*H*$_2$-triaz), 60.3 (C$_{Lac}$-6, C$_{Lac}$-6'), 61.4-59.6 (m, 7 x C-6), 51.8 (C$_{Sia}$-5), 50.0 (C-15), 62.6-62.4 (m, C-7, C-7', C-10, C-11), 42.8-41.5 (m, C-8, C-8', C-9, C-12, C-13), 40.0 (C$_{Sia}$-3), 38.0-37.0 (C-15, 3 x C$_{Ada}$-a, 3 x C$_{Ada}$-c), 29.0 (C-14), 28.4-28.1 (m, 3 x C$_{Ada}$-b), 22.1 (C$_{Sia}$-NHC(O)*CH$_3$*).

HR-MS (ESI-TOF) m/z: [M + H]$^+$; calcd for $C_{89}H_{147}N_6O_{52}$ 2131.9037; found: 2131.9118 (err: 3.8 ppm).

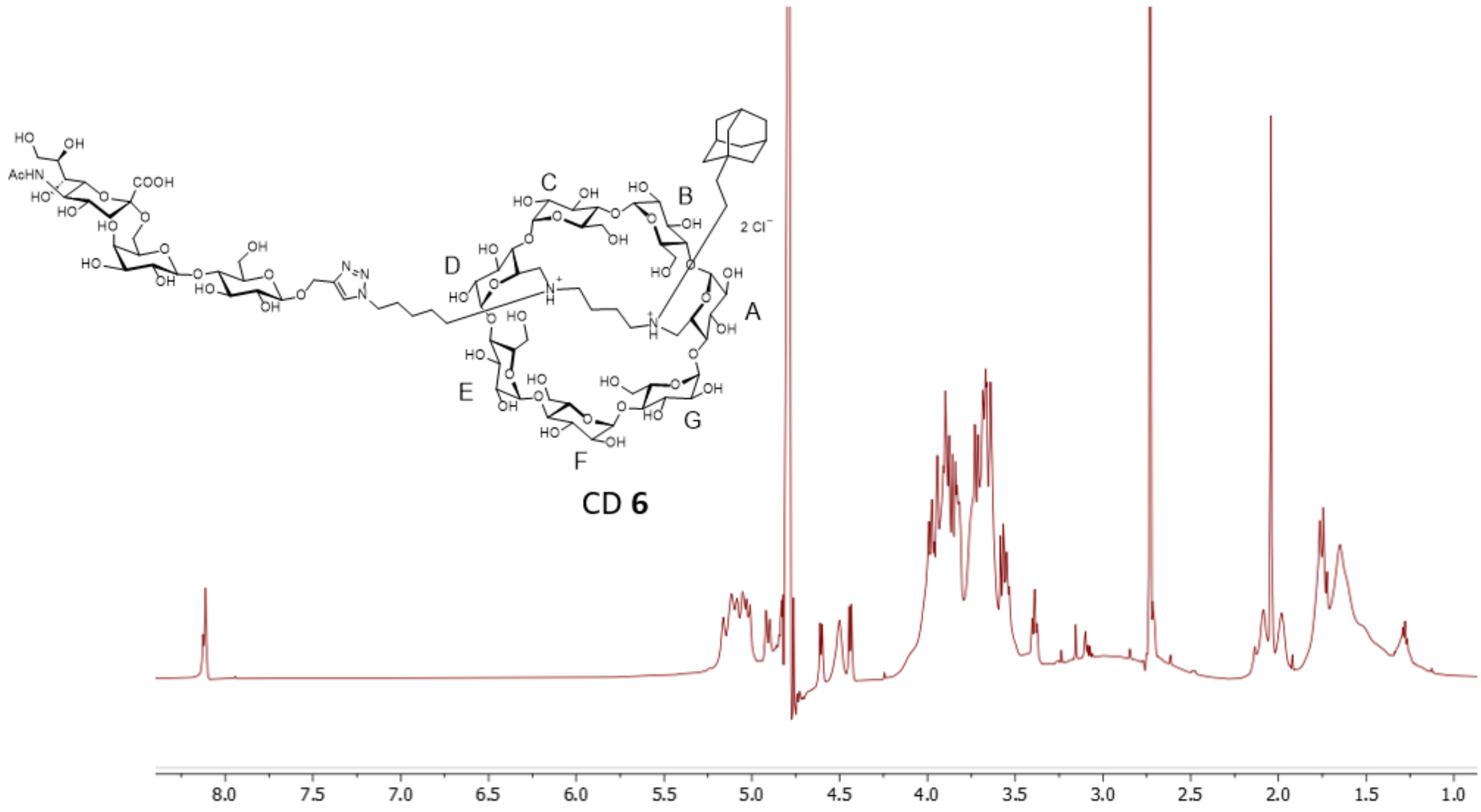

^{1}H-NMR (600 MHz, D_2O, 300K) of **CD6**

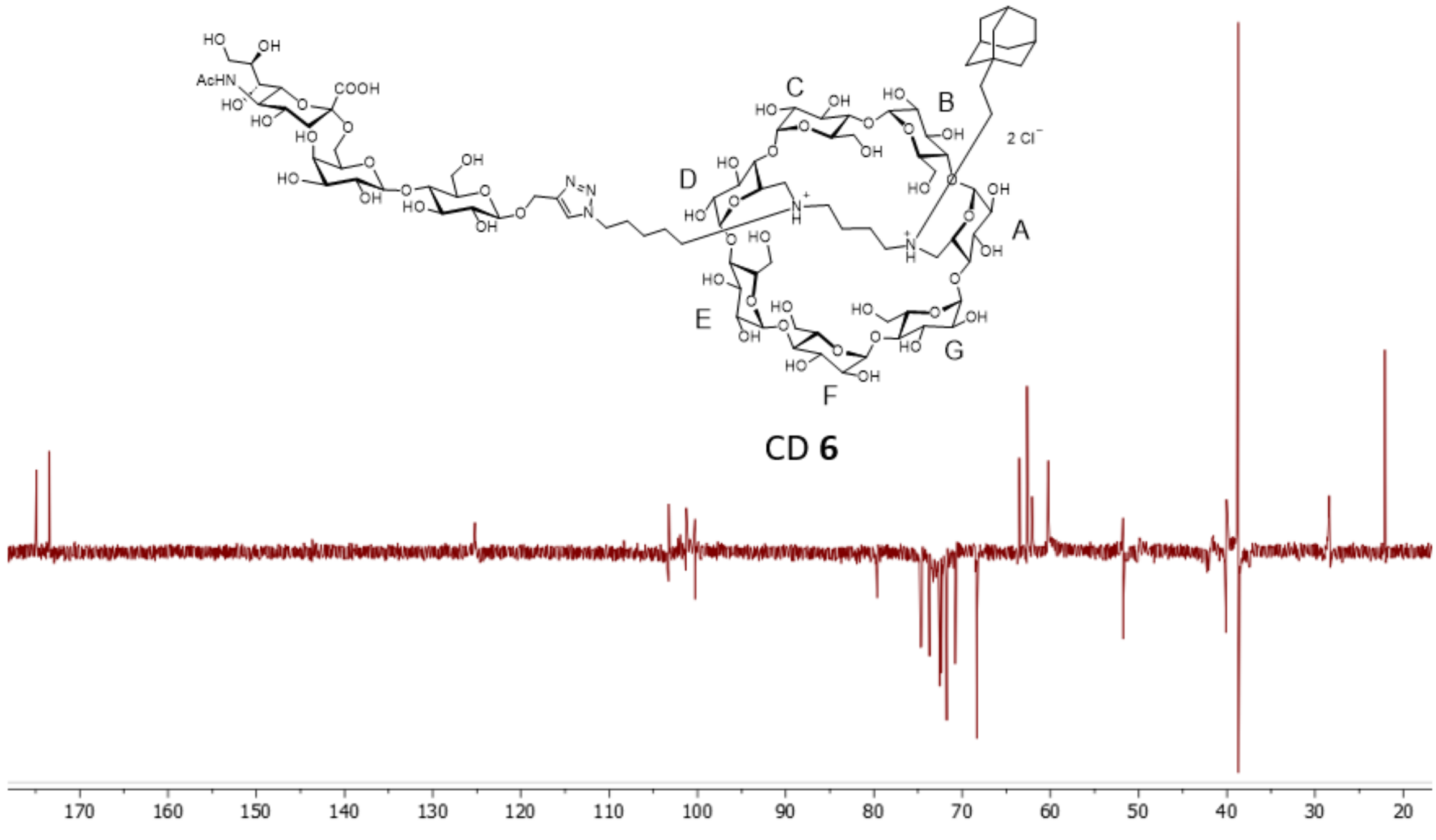

^{13}C-NMR (150 MHz, D_2O, 300K) of **CD6**

6^A, 6^D-Dideoxy-6^A,6^D-diamino-*N,N'*-butyl-*N*-(ethyladamantyl)-*N'*-(5-((4-*O*-β-D-galactopyronaoyl)-β-D-glucopyranoside)-methyl-1H-1,2,3-triazoly-4-yl)-pentyl)-β-cyclodextrin chlorohydrate (**CD7**)

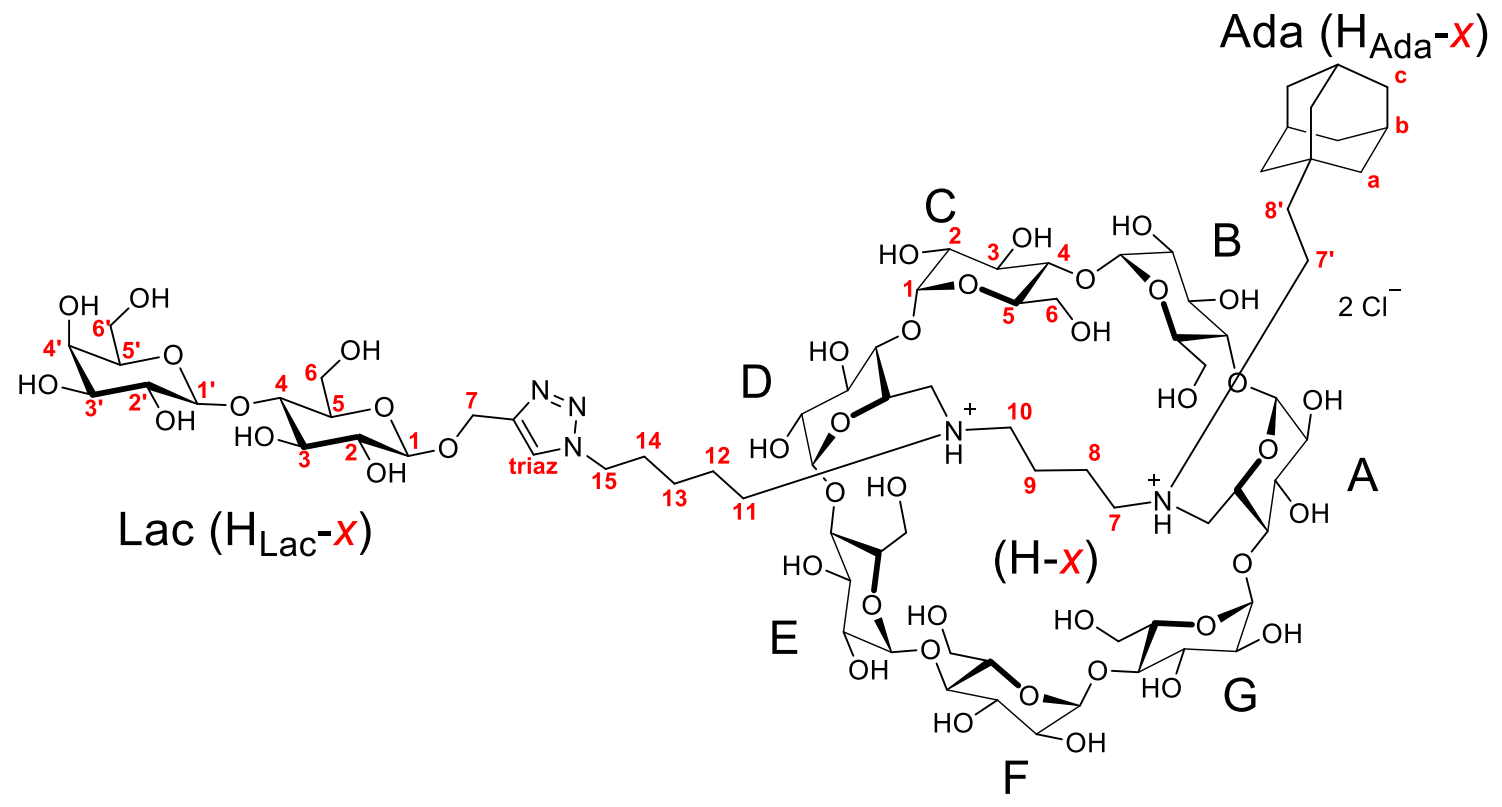

CD7 was obtained by following the *general method A* using CD **8** (22 mg, 0.01 mmol) and compound **12** (14.1 mg, 0.03 mmol), yielding **CD7** as a white fluffy solid (23 mg, 0.01 mmol, 83%).

R_f = 0.5 [^{i}PrOH/NH_3 (aq. 30%)/H_2O, 6:3:1].

^{1}H-NMR (600 MHz, D_2O, 300K): 8.11 (s, 1 H, H-triaz), 5.23-4.93 (m, 8 H, 7 x H-1, H_{Lac}-7a), 4.92 (d, $J_{Lac7a,Lac7b}$ = 12.5 Hz, 1 H, H_{Lac}-7b), 4.62 (d, $J_{1',2'}$ = 7.7 Hz, 1H, H_{Lac}-1'), 4.54-4.48 (br. s, 2 H, H-15), 4.47 (d, $J_{1,2}$ = 7.8 Hz, 1H, H_{Lac}-1), 4.17-3.75 (m, 27 H, 7 x H-3, 14 x H-6, H_{Lac}-4, H_{Lac}-4', H_{Lac}-5, H_{Lac}-5', H_{Lac}-6a, H_{Lac}-6a'), 3.75-3.55 (m, 21 H, 7 x H-2, 7 x H-4, 7 x H-5, H_{Lac}-2, H_{Lac}-6b, H_{Lac}-3', H_{Lac}-6,'), 3.42 (m, 1H, H_{Lac}-2'), 3.00-2.31 (br. m, 8 H, H-7, H-7', H-10, H-11), 2.07 (br. s, 3 H, H_{Ada}-b), 1.98 (br. s, 2 H, H-14), 1.80-1.50 (m, H-8, H-8', H-9, H-12, 6 x H_{Ada}-a, 6 x H_{Ada}-c), 1.50-1.24 (m, 2 H, H-13).

^{13}C-NMR (150 MHz, D_2O, 300K) was assigned by correlation with ^{1}H,^{13}C-HSQC: 125.3 (C-triaz), 103.2 (C_{Lac}-1), 103.0-101.6 (m, 7 x C-1), 101.3 (C_{Lac}-1'), 79.8, (C_{Lac}-2), 75-74.4 (m, 7 x H-4), 73.8 (C_{Lac}-3'), 73.6-70.8 (m, 7 x C-2, 7 x C-3, 7 x C-4, 7 x C-5), 72.7(C_{Lac}-2'), 72.6, 72.4, 71.8, 70.8, 68.5, 67.5, 62.1 (s, C_{Lac}-7), 60.3 (C_{Lac}-6, C_{Lac}-6'), 61.4-59.6 (m, 7 x C-6), 50.0 (C-15), 62.6-62.4 (m, C-7, C-7', C-10, C-11), 42.8-41.5 (m, C-8, C-8', C-9, C-12, C-13), 38.0-37.0 (m, C-15, 3 x C_{Ada}-a, 3 x C_{Ada}-c), 29.0 (C-14), 28.4-28.1 (m, 3 x C_{Ada}-b).

HR-MS (ESI-TOF) m/z: $[M + H]^+$; calcd for $C_{78}H_{130}N_6O_{44}$ 1840.8083; found: 1840.8173 (err: 4.9 ppm).

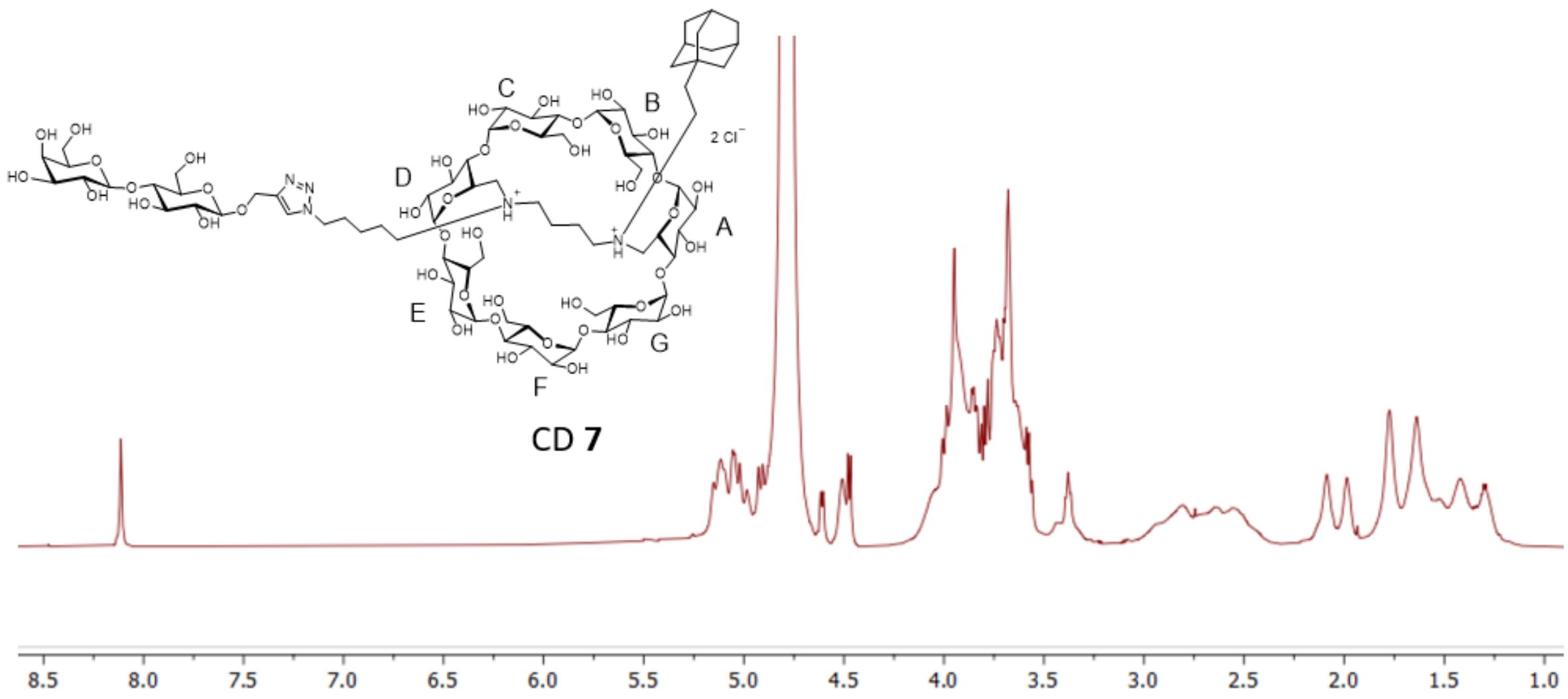

^{1}H-NMR (600 MHz, D_2O, 300K) of **CD7**

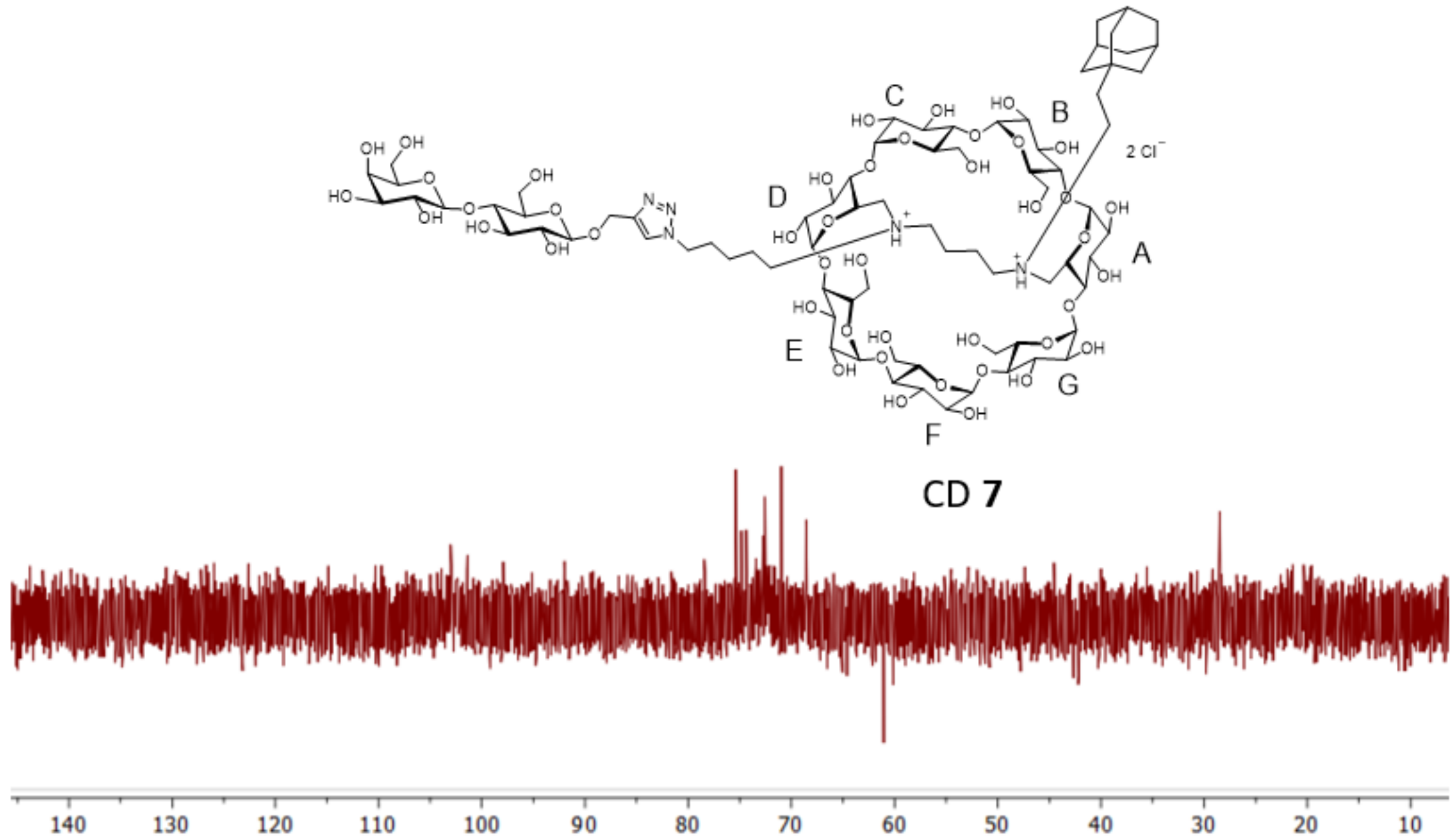

^{13}C-NMR (150 MHz, D_2O, 300K) of **CD7**

6[A], 6[D]-Dideoxy-6[A],6[D]-diamino-*N,N'*-butyl-*N*-(ethyladamantyl)-*N'*-(5-azidopentyl)-β-cyclodextrin chlorohydrate (**CD8**)

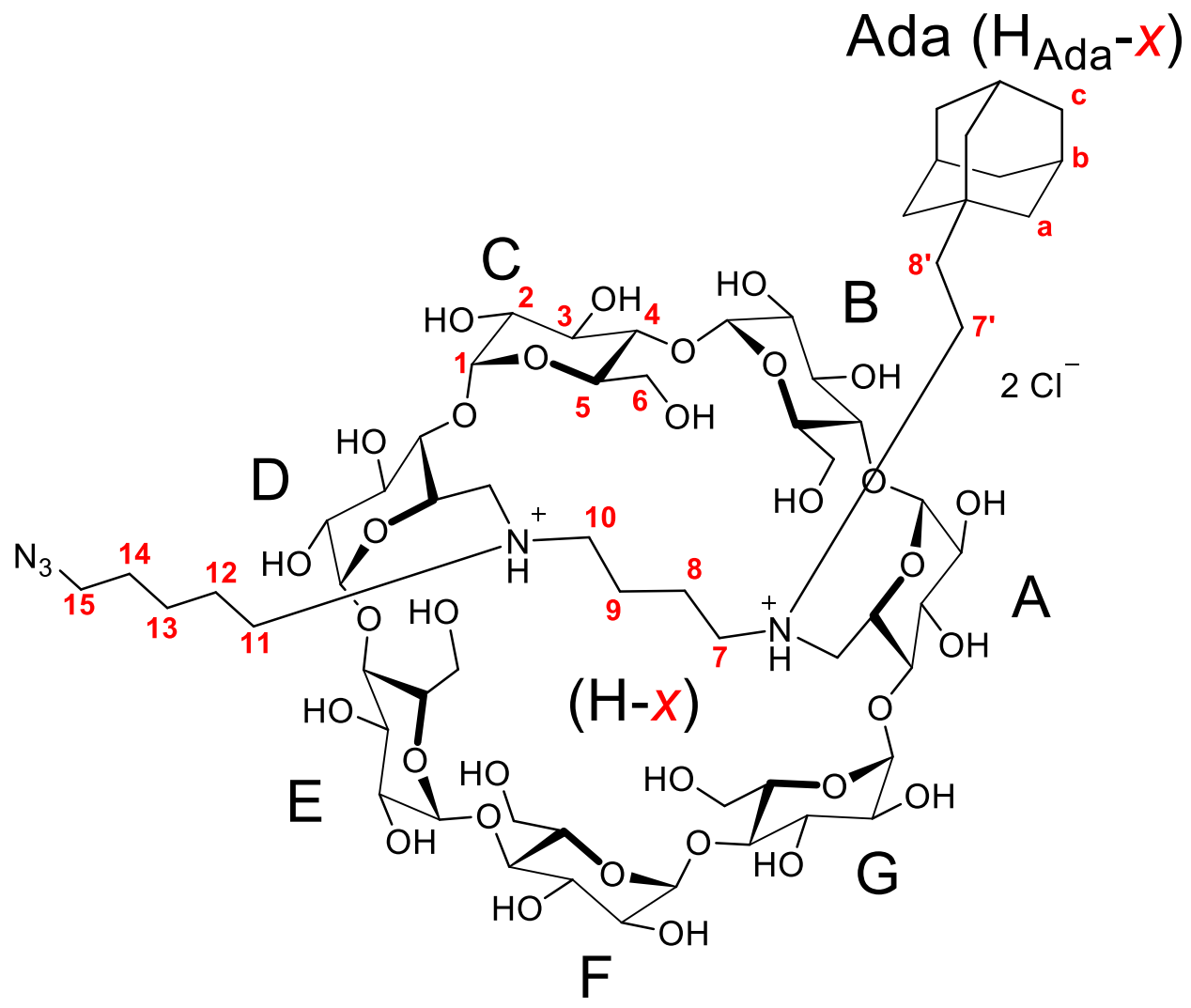

Adapted from Evenou *et al.*[6] Adamantyl-functionalised **CD1** (614 mg, 0.455 mmol, 1 eq.) was suspended in DMF (10 mL) at room temperature and the flask was flushed with Argon. 5-Azidopentanal[7] (145 mg, 1.14 mmol, 2.5 eq.) was added to the mixture, which was stirred for 30 minutes at room temperature. At that point, STAB (265 mg, 1.25 mmol, 2.75 eq.) was added, the flask was flushed with Argon, and the mixture was stirred for 3 hours, until MS confirmed the complete conversion of the starting material. Next, the solvent was evaporated and a dry loading was prepared with the crude residue. After loading on a silica plug (twice as big as the silica loading), which was washed with 10 CV of ^{i}PrOH/NH_3 (aq. 30%), 8:2. The product was eluted with 10 CV of ^{i}PrOH/NH_3 (aq. 30%)/H_2O, 6:3:1, and the solvents were evaporated under reduced pressure. Finally, the purified material was freeze-dried overnight to obtain **CD8** as a white fluffy solid (537 mg, 0.368 mmol, 81%).

R_f = 0.32 [^{i}PrOH/NH_3 (aq. 30%)/H_2O, 6:3:1].

^{1}H-NMR (600 MHz, D_2O, 300K): 5.22-5.15 (2 x br. s, 3 H, H-1), 5.11 (br. s, 3 H, H-1), 5.06 (br. s, 1 H, H-1). 4.02-3.84 (m, 18 H, H-3, H-6), 3.83-3.55 (br. m, 24 H, H-2, H-4, H-5, H-6), 3.42-3.28 (br. m, 8 H, H-7, H-10, H-11, H-15), 2.17 (br. s, 3 H, H_{Ada}-b), 1.92-1.73 (br. m, 16 H, H-7', H-8', H_{Ada}-a, H_{Ada}-c), 1.73-1.44 (br. m, 10 H, H-9, H-10, H-12, H-13, H-14).

^{13}C-NMR (150 MHz, D_2O, 300K) was assigned by correlation with ^{1}H,^{13}C-HSQC: 102.8, 102.2, 101.8, 101.5, 100.9, 99.8 (7 C, 7 x C-1), 75-70 (m, 7 x C-2, 7 x C-3, 7 x C-4, 7 x C-5), 61.5-60 (m, 7 x C-6), 50.8 (app. s, 4 C, C-7, C-10, C-11, C-15), 41.5 (app. s, 11 C, 3 x C_{Ada}-a, 3 x C_{Ada}-b, 3 x C_{Ada}-c, C-7', C-8'), 37.2, 37.3 (2 C, C-8, C-9), 27.6, 23.0 (app. s, 3 C, C-12, C-13, C-14).

HR-MS (ESI-TOF) m/z: [M + H]$^+$; calcd for $C_{63}H_{106}N_5O_{33}$ 1460.6765; found: 1460,6709 (err: 3.8 ppm).

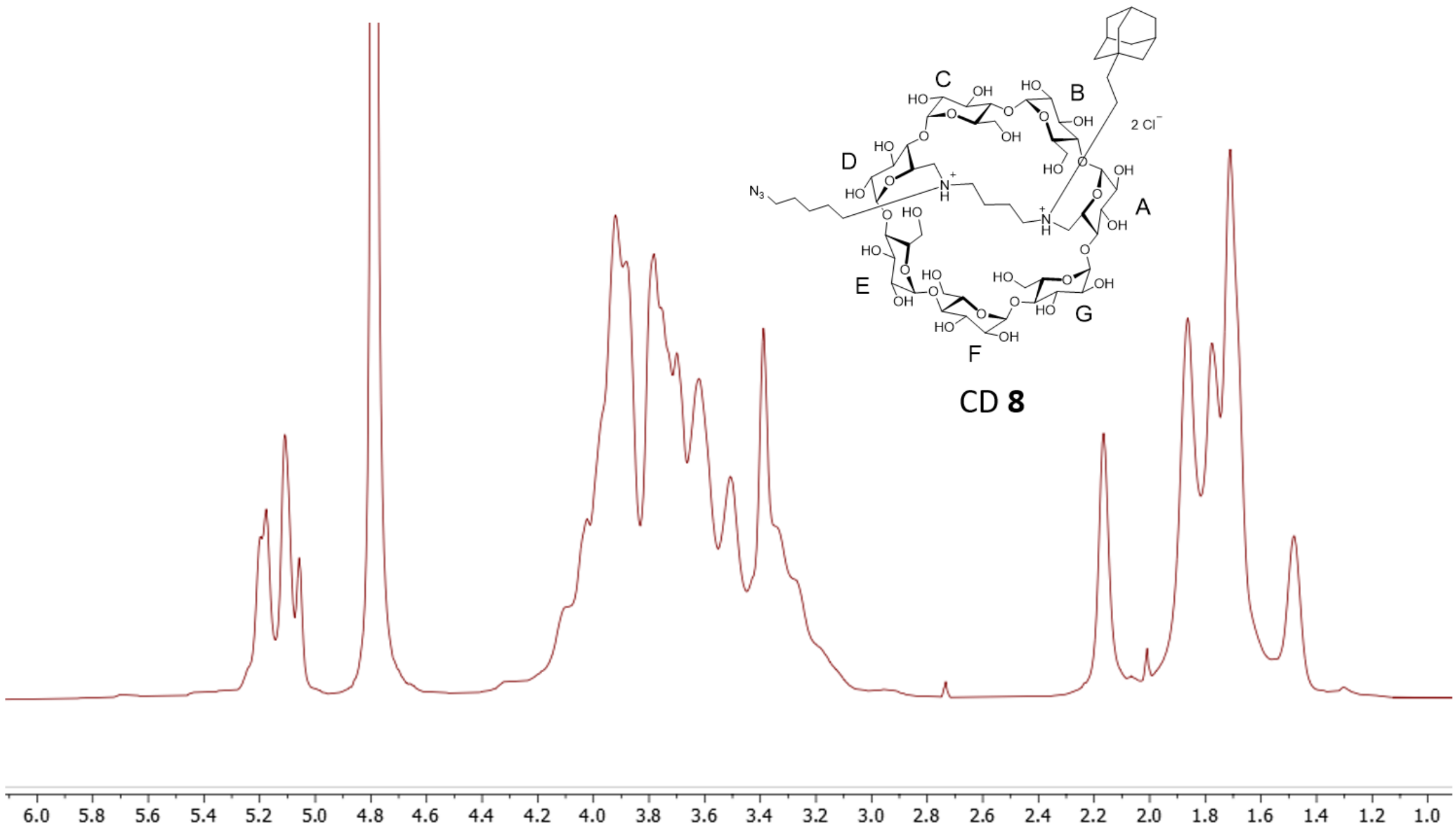

^{1}H-NMR (600 MHz, D_2O, 300K) of **CD8**

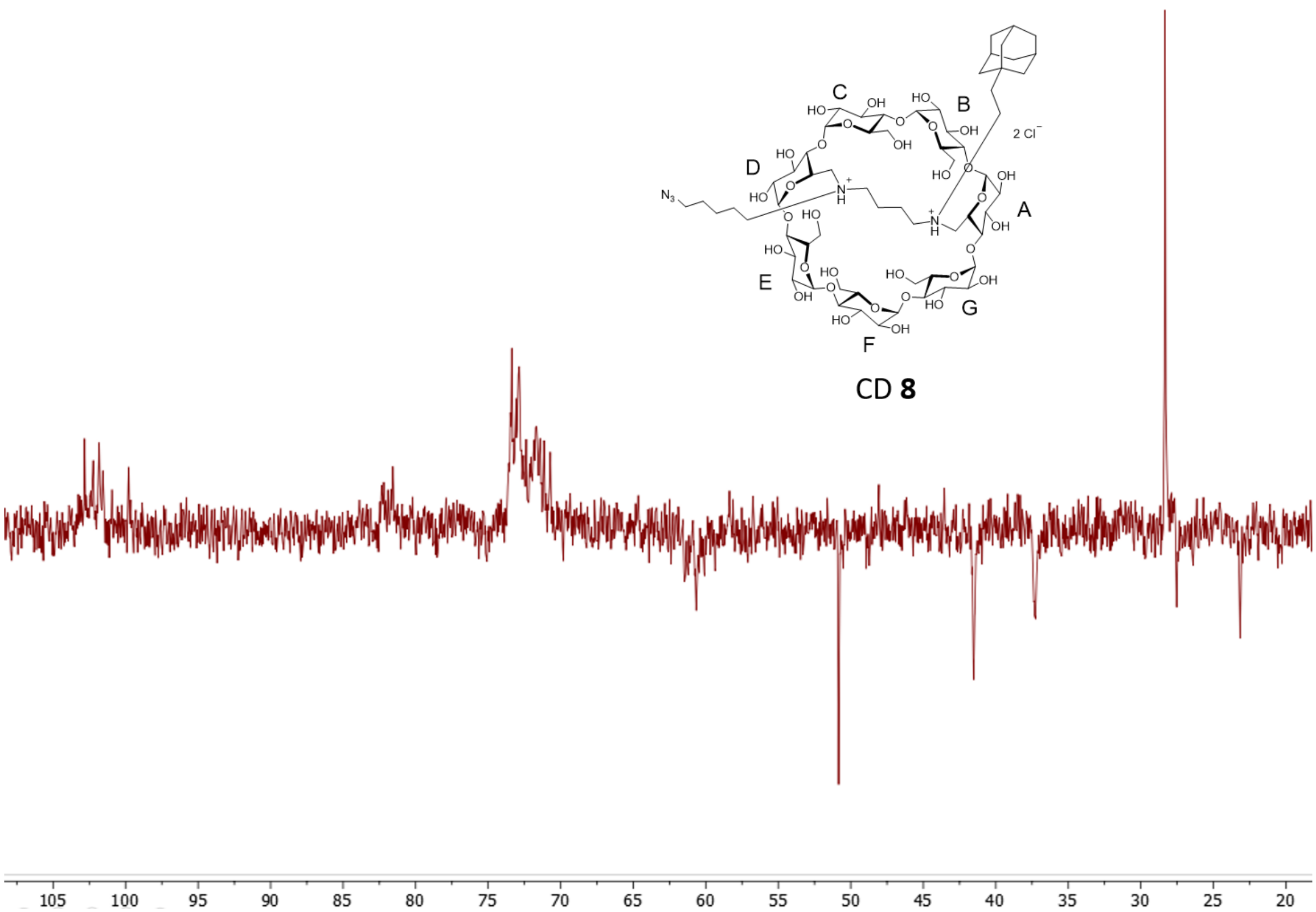

^{13}C-NMR (150 MHz, D_2O, 300K) of **CD8**

6^{A}, 6$^{D/E}$-Dideoxy-6^{A},6$^{D/E}$-diamino-*N,N'*-butyl-*N'*-(5-azidopentyl)-β-cyclodextrin chlorohydrate (**CD8b**)

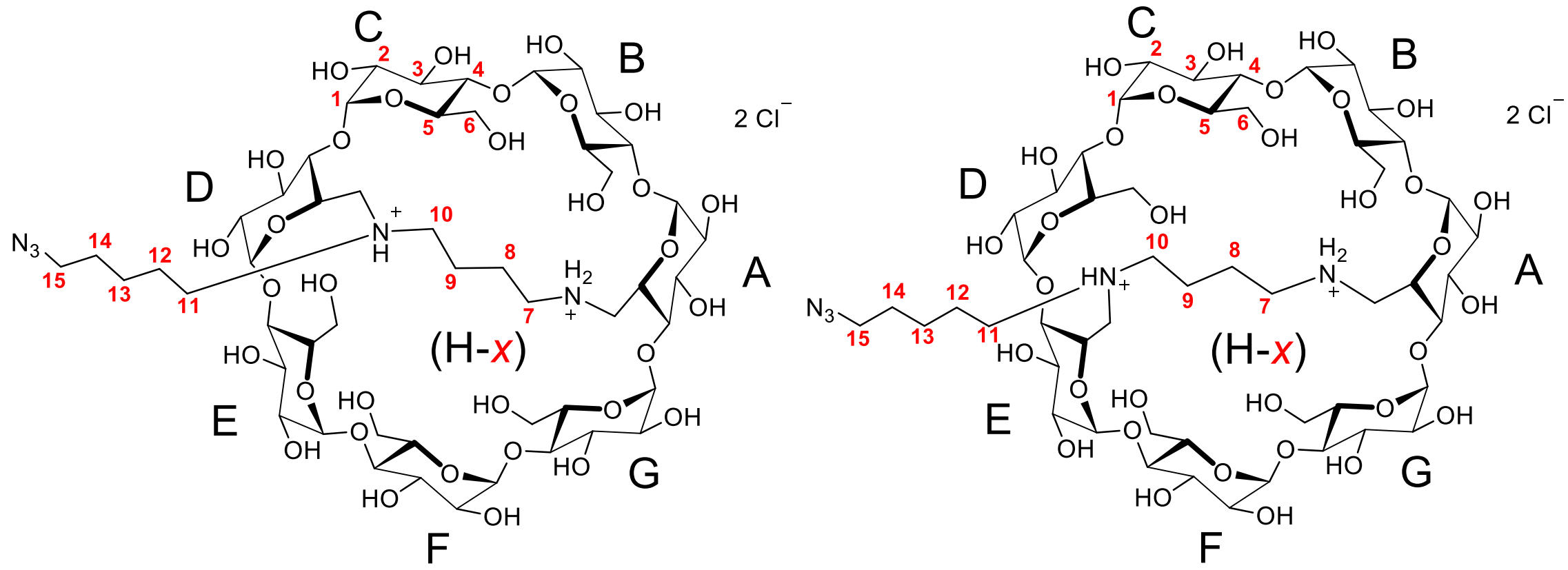

Bridged β-CD[6] (455 mg, 0.38 mmol, 1 eq.) was suspended in DMF (130 mL) at room temperature and the flask was flushed with Argon. 5-Azidopentanal[7] (44 mg, 0.35 mmol, 0.9 eq.) was added to the mixture, which was stirred for 30 minutes at room temperature. At that point, STAB (203 mg, 0.96 mmol, 2.5 eq.) was added, the flask was flushed with Argon, and the mixture was stirred for 3 hours, until TLC (^{i}PrOH/NH_3 (aq. 30%)/H_2O, 6:3:1) started to show the appearance of the disubstituted byproduct (R_f 0.61). Next, the solvent was evaporated and the crude was triturated (3 x 10 mL) with acetone to remove potential linker left. The residue was then redissolved in water and loaded in a C18 column to be purified with a H_2O/ACN gradient 0-30%. Finally, the purified material was freeze-dried overnight to obtain **CD8b** a white fluffy solid mixture of the AD and AE regioisomers (230 mg, 0.18 mmol, 46%).

R_f = 0.45 [^{i}PrOH/NH_3 (aq. 30%)/H_2O, 6:3:1]

^{1}H-NMR (600 MHz, D_2O, 300K): 5.30 (d, $J_{1\text{-}2}$ = 5.6 Hz, 1 H, H-1), 5.21-5.07 (m, 6 H, 6 x H-1), 4.46-4.28 (m, 2 H, H-5$_{A}$, H-5$_{E/D}$), 4.10-3.90 (m, 22 H, 7 x H-3, 7 x H-6a), 3.80-3.60 (m, 12 H, 7 x H-2, 5 x H-5), 3.60-3.45 (m, 7 H, 7 x H-4), 3.40-3.10 (m, 8 H, H-7, H-10, H-11, H-15), 1.95-1.75 (m, 6 H, H-8/9, H-12/13/14), 1.70 (app. p, 2 H, H-8/9, H-12/13/14), 1.55-1.45 (m, 2 H, H-8/9, H-12/13/14).

^{13}C-NMR (150 MHz, D_2O, 300K) was assigned by correlation with ^{1}H,^{13}C-HSQC: 103-99 (7 x C-1), 74-78 (m, 7 x C-4, 5 x C-5), 70-74 (m, 7 x C-2, 7 x C-3), 64.35-64.20 (m, C-5$_{A}$, C-5$_{D/E}$), 62-59 (m, 6 x C-6), 54-50 (m, C-7/8/9/10, C-12/13/14), 28-23 (m, C-7/8/9/10, C-12/13/14).

HR-MS (ESI-TOF) m/z: [M + 2 H]$^{+}$; calcd for $C_{51}H_{89}N_5O_{33}$ 649.7721; found: 649.7714 (err: 1.0 ppm).

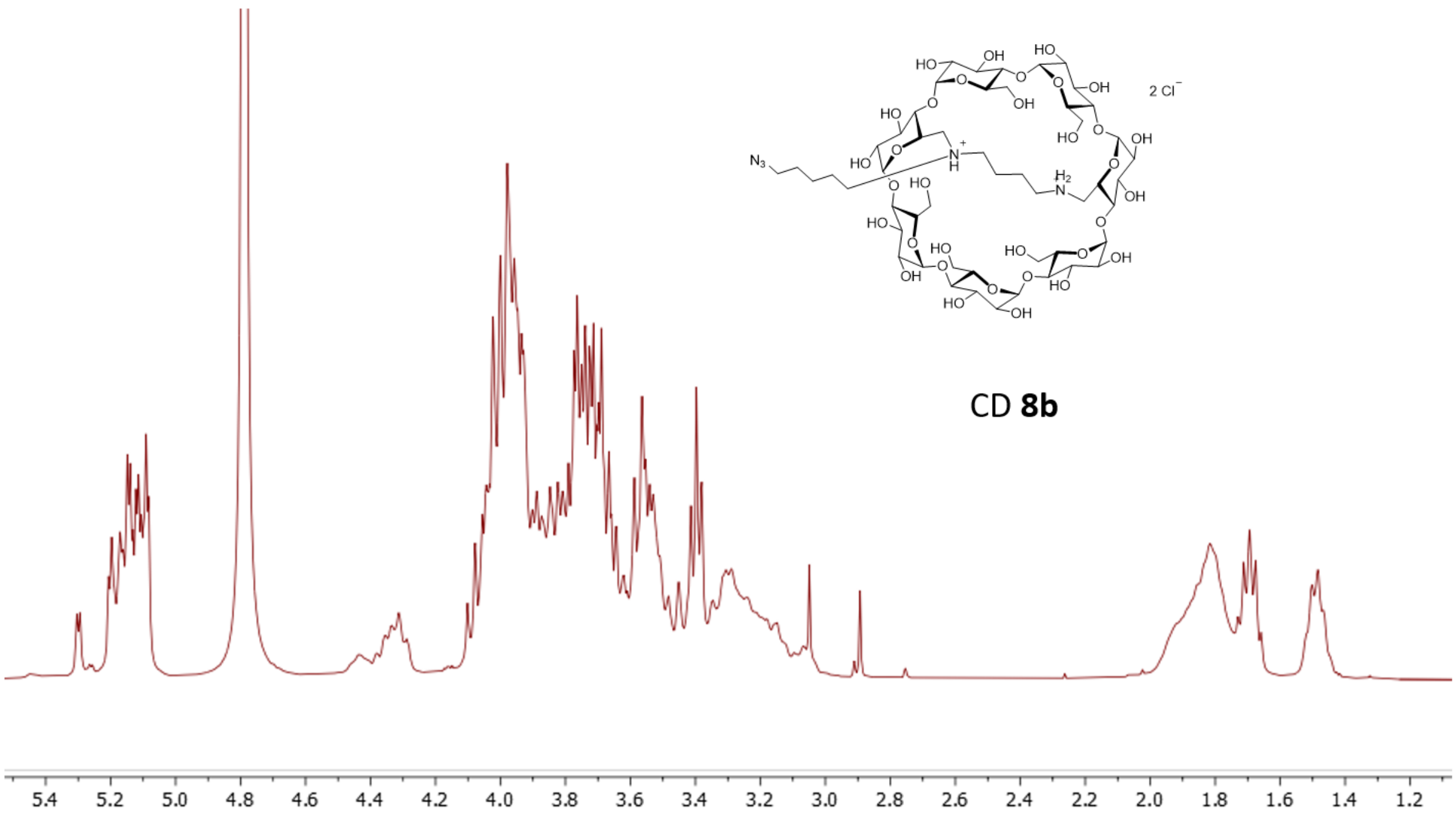

^{1}H-NMR (600 MHz, D_2O, 300K) of **CD8b**

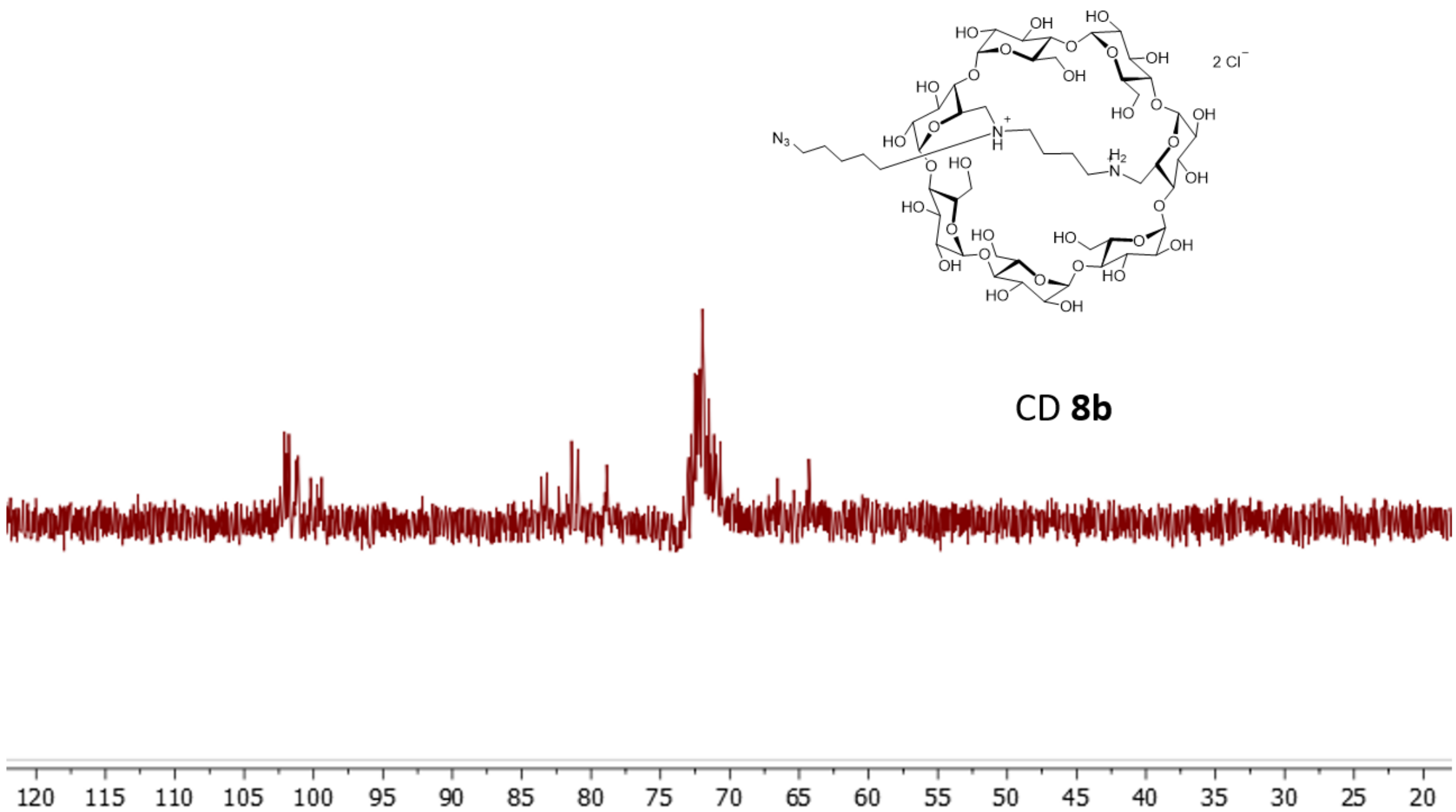

^{13}C-NMR (150 MHz, D_2O, 300K) of **CD8b**

SI.2.3 Carbohydrate synthesis

Propargyl 5-acetamido-9-*O*-acetyl-3,5-dideoxy-D-glycero-α-D-galacto-2-nonulopyranoside (**11**)

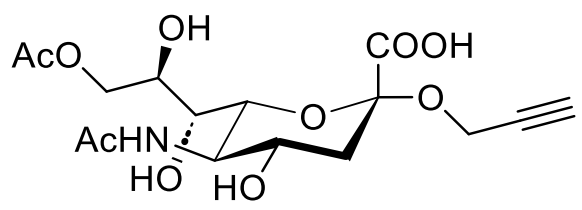

Adapted from Petitjean *et al.*[8] To a solution of **10** (80 mg, 0.23 mmol, 1 eq.) in MeOH (2.5 mL), trimethyl orthoacetate (0.28 mL, 2.2 eq.) and *p*-toluenesulfonic acid (2 mg, 0.01 mmol, 0.05 eq.) were added. The mixture was stirred overnight at room temperature, at which point a dry loading was prepared with reaction crude. The desired product was purified by silica gel flash chromatography (DCM/MeOH, 0-20%) and the collected fractions were dried under reduced pressured and finally freeze-dried to obtain **11** as a white, fluffy solid (74 mg, 0.19 mmol, 88%).

R_f = 0.54 [EtOAc/MeOH/H_2O/HOAc, 4:2:1:0.1].

NMR data was in agreement with literature.[8]

ESI-TOF m/z: $[M - H]^-$; calcd for $C_{16}H_{22}NO_{10}$ 388.1249; found: 388.0110.

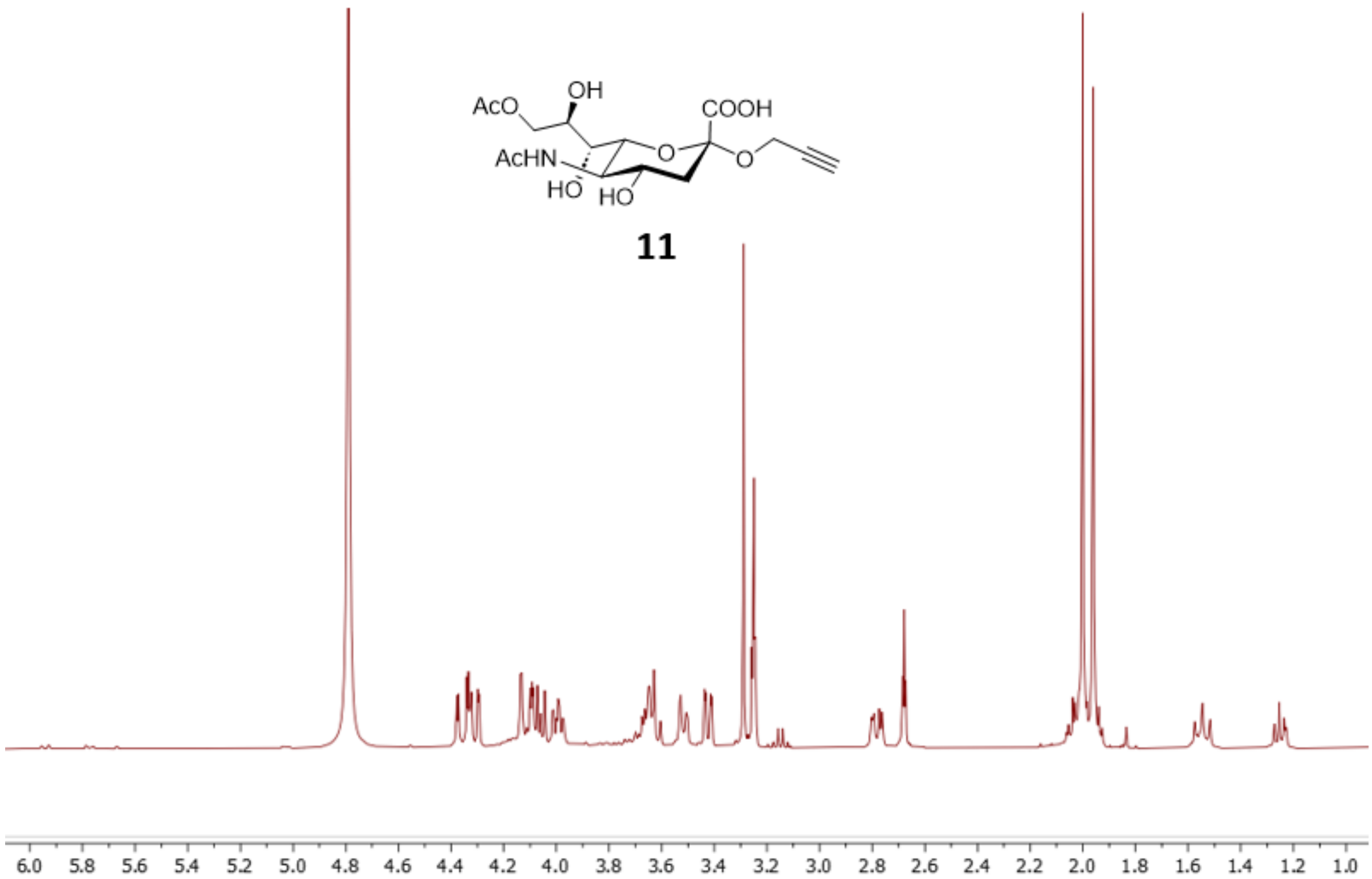

^{1}H-NMR (400 MHz, D_2O, 300K) of compound **11**

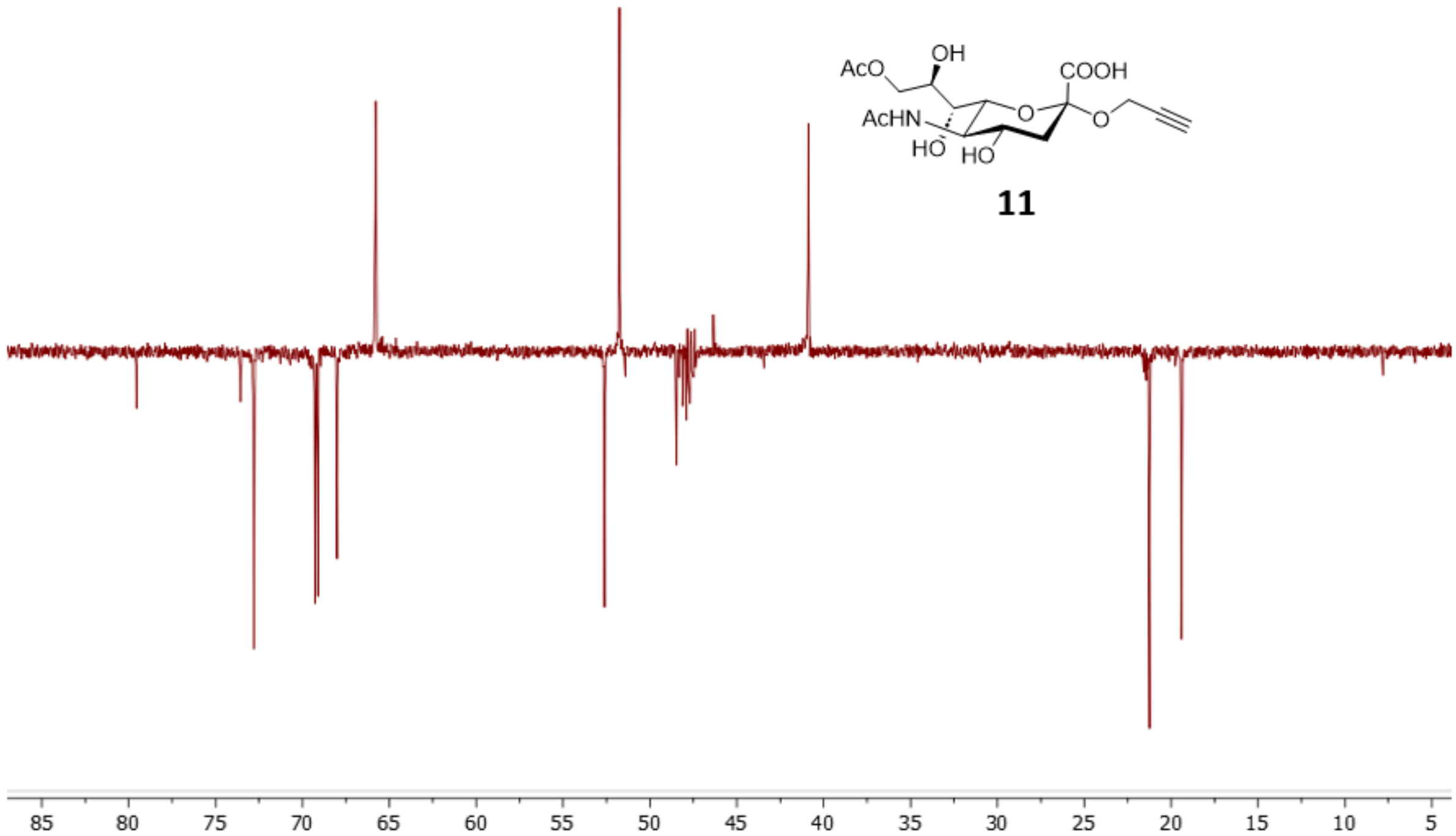

^{13}C-NMR (150 MHz, D_2O, 300K) of compound **11**

Propargyl 4-*O*-(3-*O*-(5-acetamido-3,5-dideoxy-D-glycero-α-D-galacto-2-nonulopyranosyl)-β-D-galactopyronaoyl)-β-D-glucopyranoside (**13**)

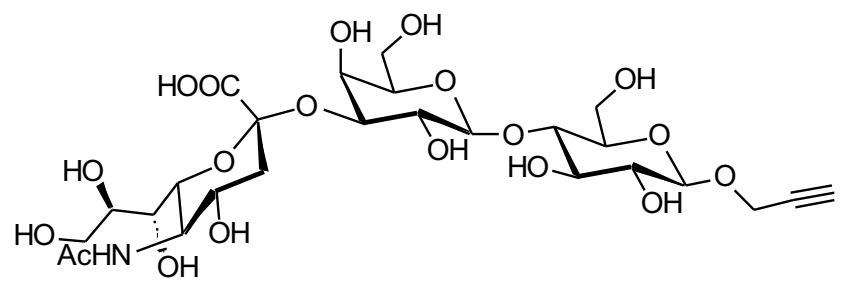

Sialyllactoside **13** was obtained by following the *general method B* using acceptor **12** (31.6 mg, 0.08 mmol) prepared according to Mereyala *et al.*[9], yielding **13** as a white fluffy solid (50 mg, 0.07 mmol, 89%).

$\mathbf{R_f}$ = 0.25 [EtOAc/MeOH/H_2O/HOAc, 4:2:1:0.1].

NMR data was in agreement with literature.[7]

ESI-TOF m/z: $[M - H]^-$; calcd for $C_{26}H_{40}NO_{19}$ 670.2200; found: 670.1390.

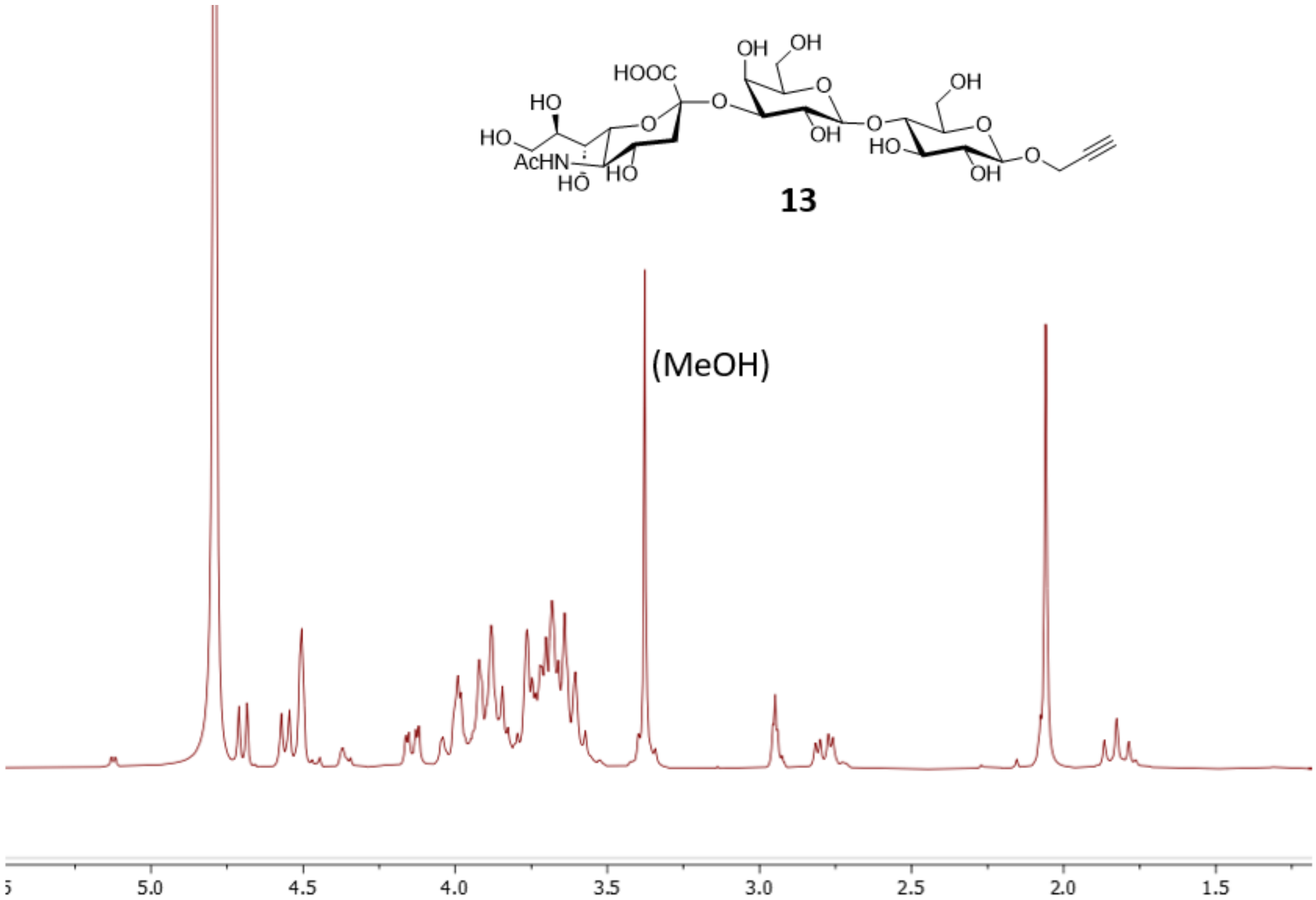

^{1}H-NMR (300 MHz, D_2O, 300K) of compound **13**

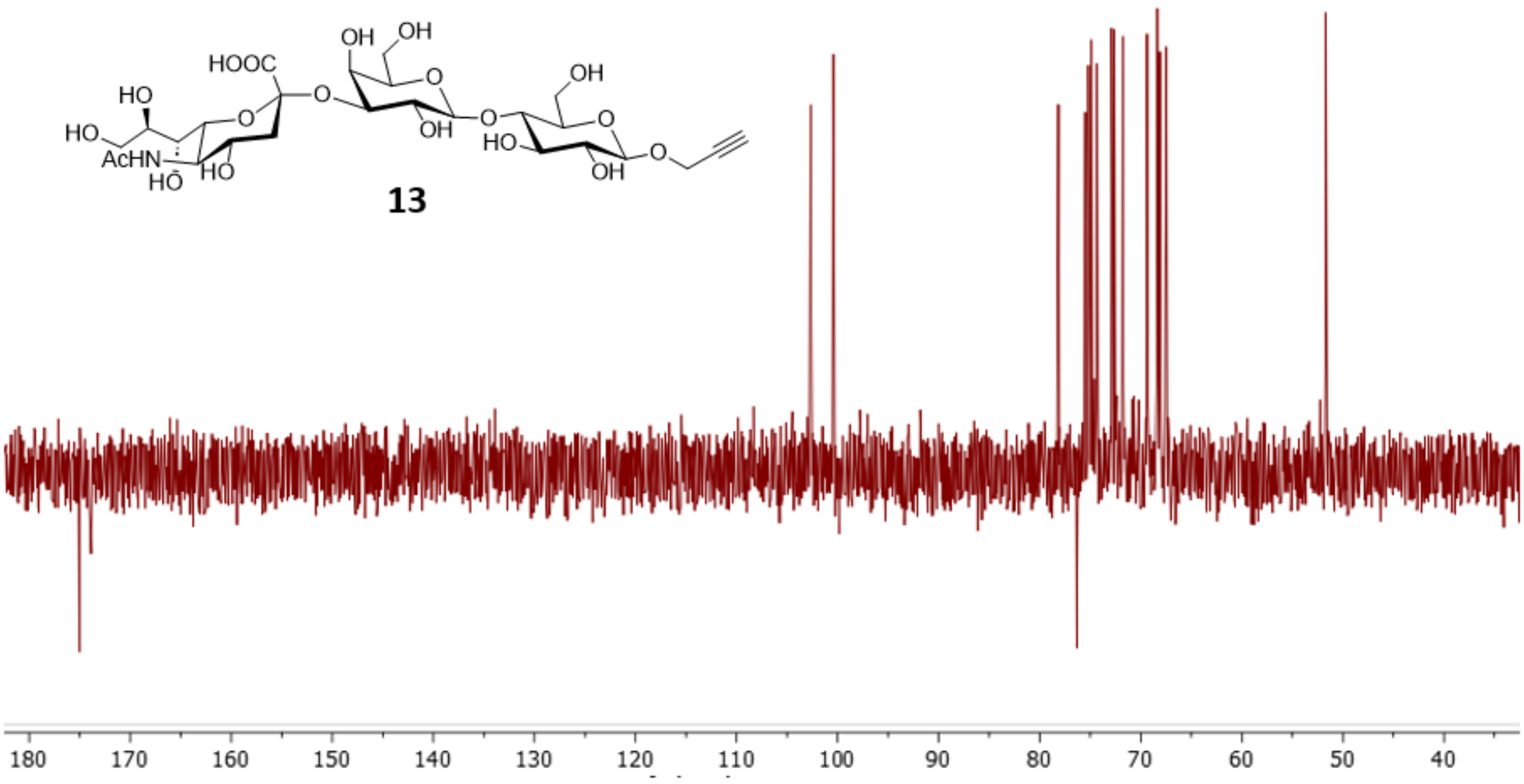

^{13}C-NMR (150 MHz, D_2O, 300K) of compound **13**

Propargyl 4-*O*-(3-*O*-(5-acetamido-3,5-dideoxy-D-glycero-α-D-galacto-2-nonulopyranosyl)-β-D-galactopyronaoyl)-β-D-glucopyranoside (**14**)

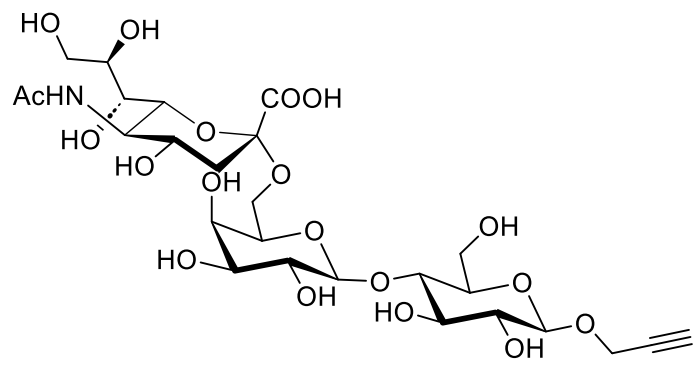

Sialyllactoside **14** was obtained by following the *general method B* using acceptor **12**[9] (23.9 mg, 0.08 mmol), yielding **14** as a white fluffy solid (33 mg, 0.05 mmol, 78%).

R_f = = 0.16 [EtOAc/MeOH/H_2O/HOAc, 4:2:1:0.1].

NMR data was in agreement with literature.[10]

ESI-TOF m/z: $[M - H]^-$; calcd for $C_{26}H_{40}NO_{19}$ 670.2200; found: 670.1765.

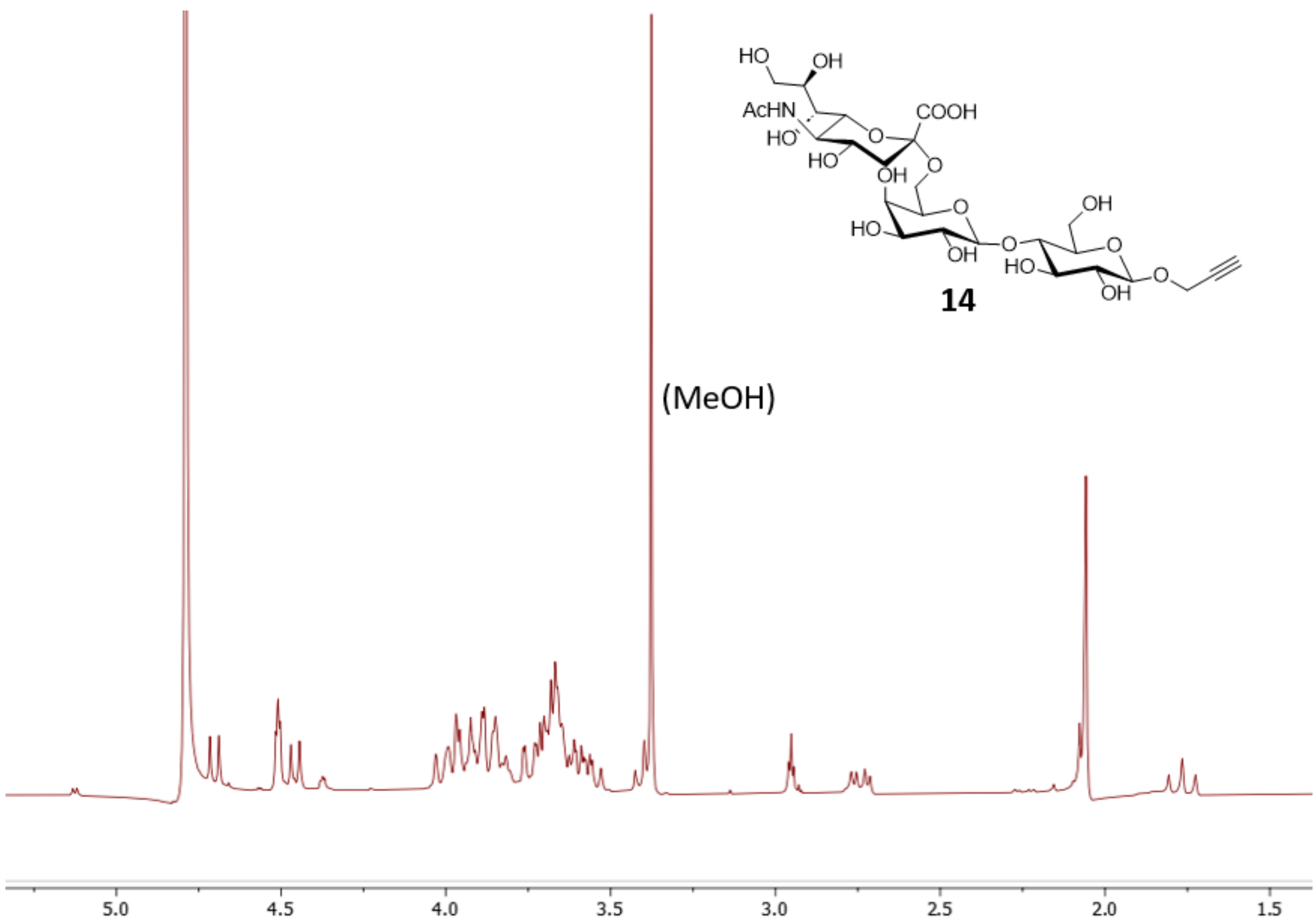

^{1}H-NMR (300 MHz, D_2O, 300K) of compound **14**

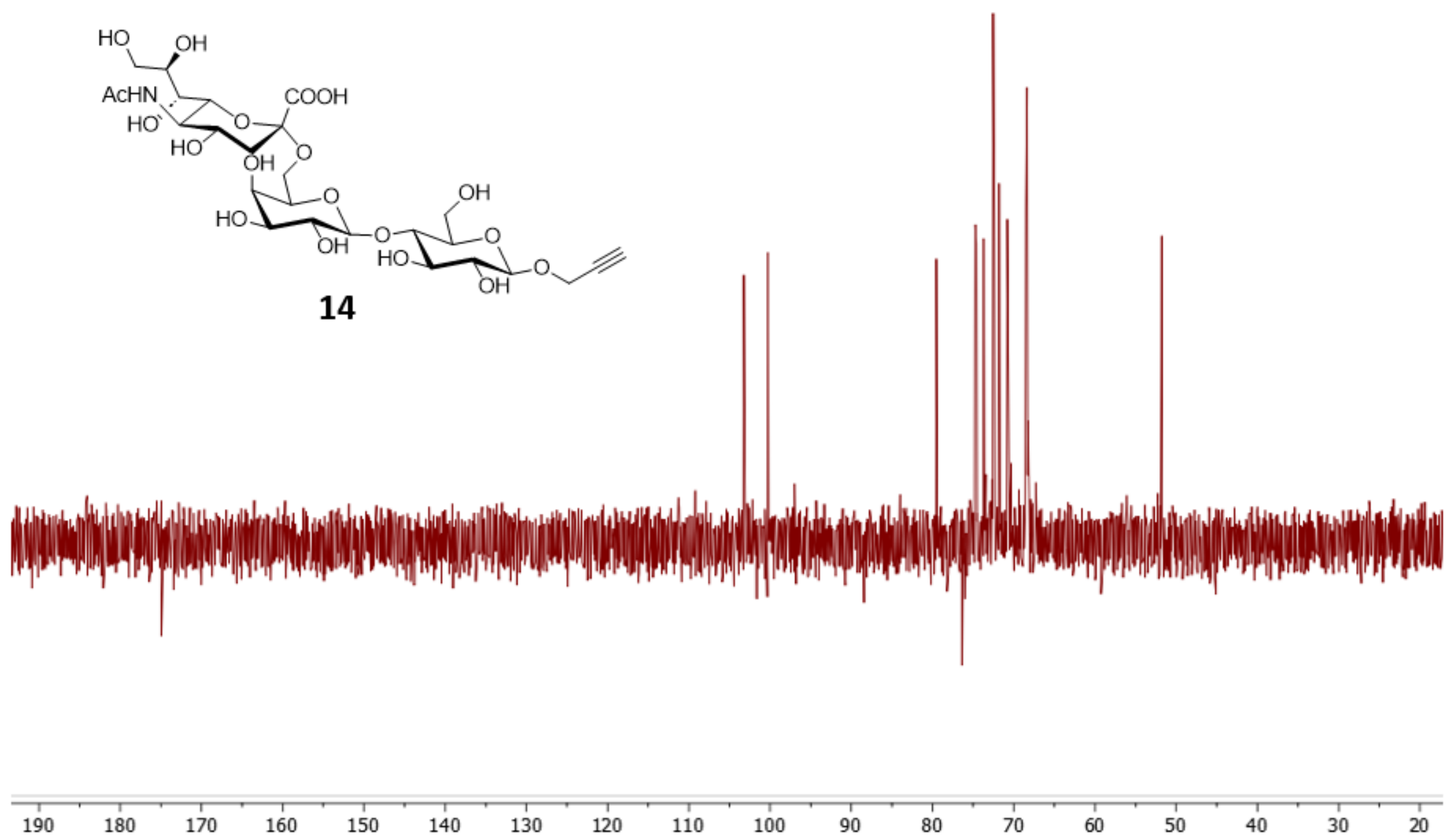

^{13}C-NMR (150 MHz, D_2O, 300K) of compound **14**

SI.3 Copper testing data

The residual amount of copper was quantified following the *general method B* for the different CD-based compounds. The corresponding results are depicted below together with the calibration curves. The residual copper concentration is first quantified at the 96-well plate level, then transposed to the tested sample level, and finally converted to a percentage relative to the CD compound. See tables in figures SI.3.1 and SI.3.2.

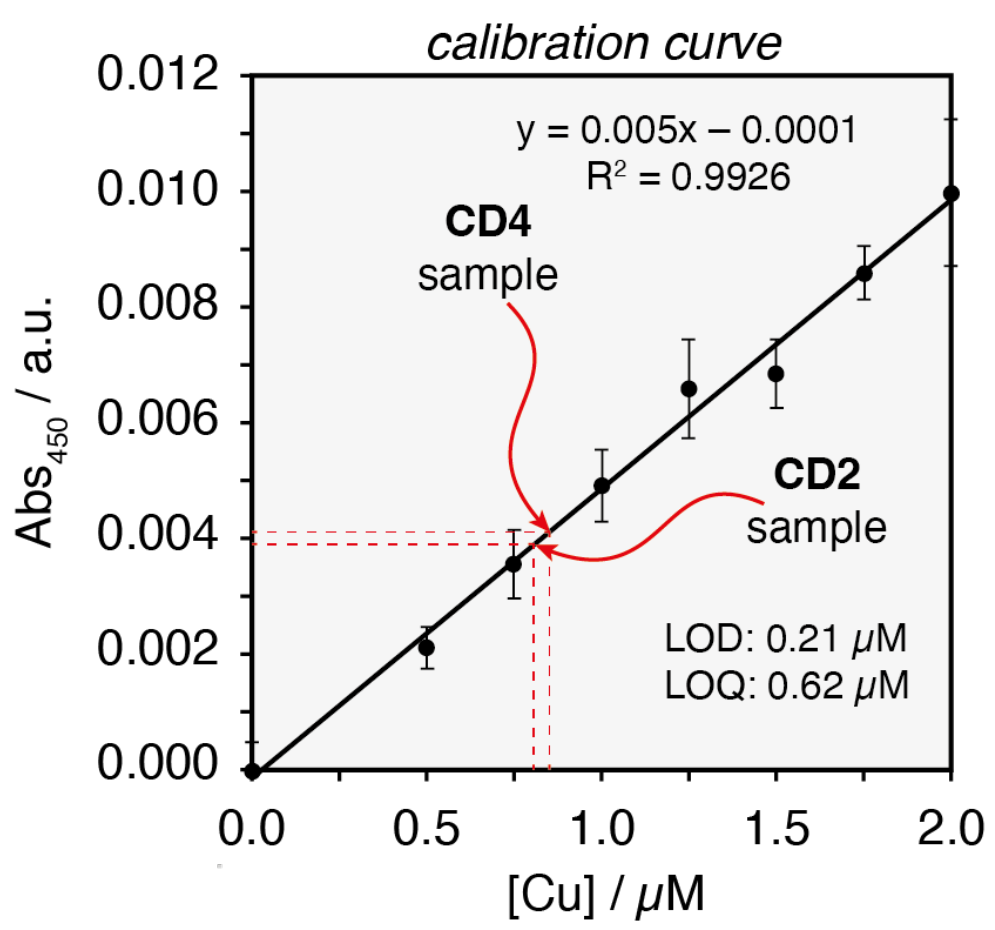

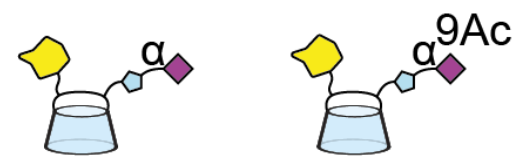

	CD2	CD4
sample Abs	0.0018	0.0019
[Cu] / μM (in the well)	0.39	0.41
[Cu] / μM (in the sample)	58	61
Cu / mol% (in the sample)	0.11	0.56

Regression Statistics	
Multiple R	0.996288
R^2	0.992590
Adjusted R^2	-1.3
Standard Err.	0.00031

Fig. SI.3.1. Regression line and copper quantification in **CD2** and **CD4**

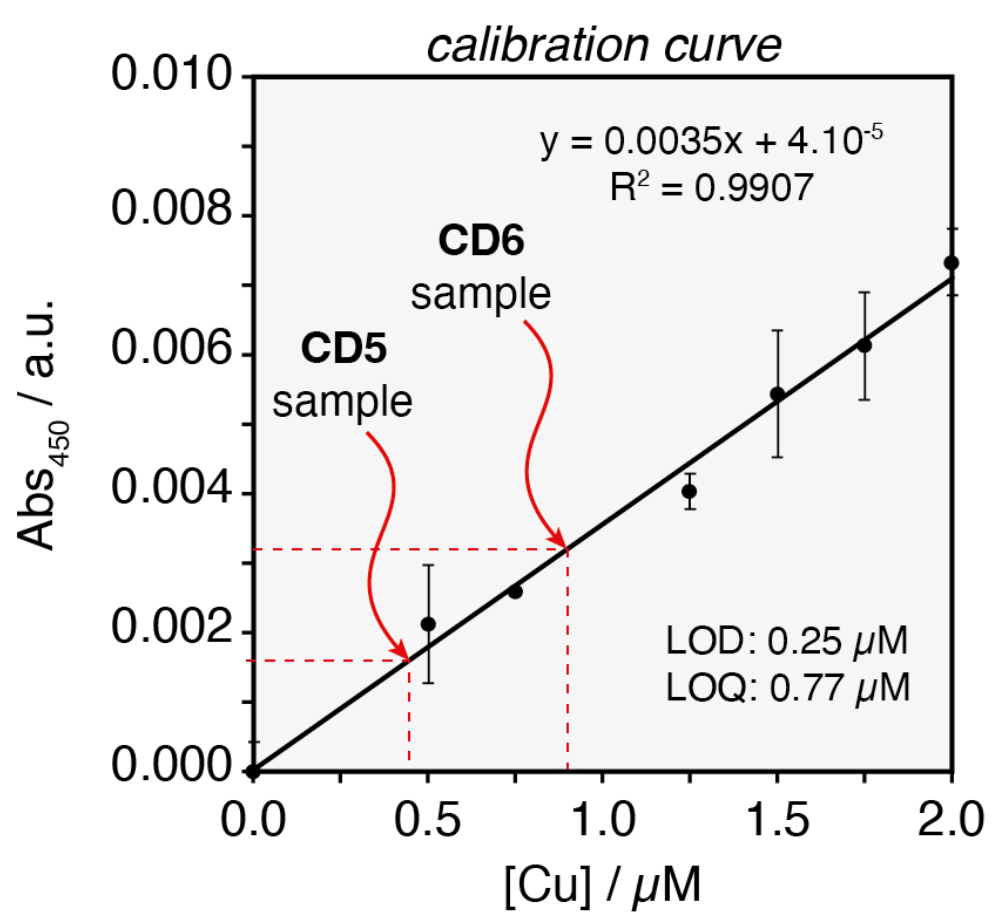

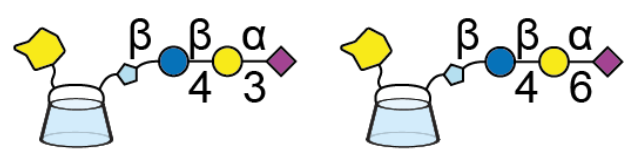

	CD5	CD6
sample Abs	0.0016	0.0032
[Cu] / μM (in the well)	0.45	0.90
[Cu] / μM (in the sample)	68	135
Cu / mol% (in the sample)	0.56	0.78

Regression Statistics	
Multiple R	0.995342
R^2	0.990706
Adjusted R^2	-1.4
Standard Err.	0.00027

Fig. SI.3.2. Regression line and copper quantification in **CD5** and **CD6**

SI.4 DOSY NMR

The diffusion coefficient provides information about the size of a molecule and, in the case of a supramolecular assembly, the degree of polymerization. For the interpretation of the data, it is assumed that the supramolecular polymer is linear and rigid, with a cylinder-like architecture whose dimensions can be estimated using equations (1) reported by Tirado-Garcia de la Torre and collaborators:[11]

$$D = \frac{k_B T}{3\pi\eta L}\left[ln\left(\frac{L}{d}\right) + \nu\right] \quad (1)$$

with

$$\nu = 0.312 + 0.565\frac{d}{L} - 0.100\left(\frac{d}{L}\right)^2 \quad (2)$$

Where:
D: translational diffusion coefficient ($m^2.s^{-1}$)
k_B: Boltzmann constant ($J.\ K^{-1}$)
T: temperature (K)
η: viscosity of the liquid (Pa.s)
L: length of cylinder (m)
d: diameter of cylinder (m)

Garcia de la Torre introduced as well a finite aspect ratio parameter, $p = \frac{L}{d}$ to express the translational diffusion coefficient D as:

$$\frac{3D\pi\eta d}{k_B T} = \frac{1}{p}\left[\ln(p) + 0.312 + \frac{0.565}{p} - \frac{0.100}{p^2}\right] \quad (3)$$

The left-hand side of the equation can be evaluated from the measured diffusion coefficient and following a simulation of the diameter (d) using Avogadro as below:

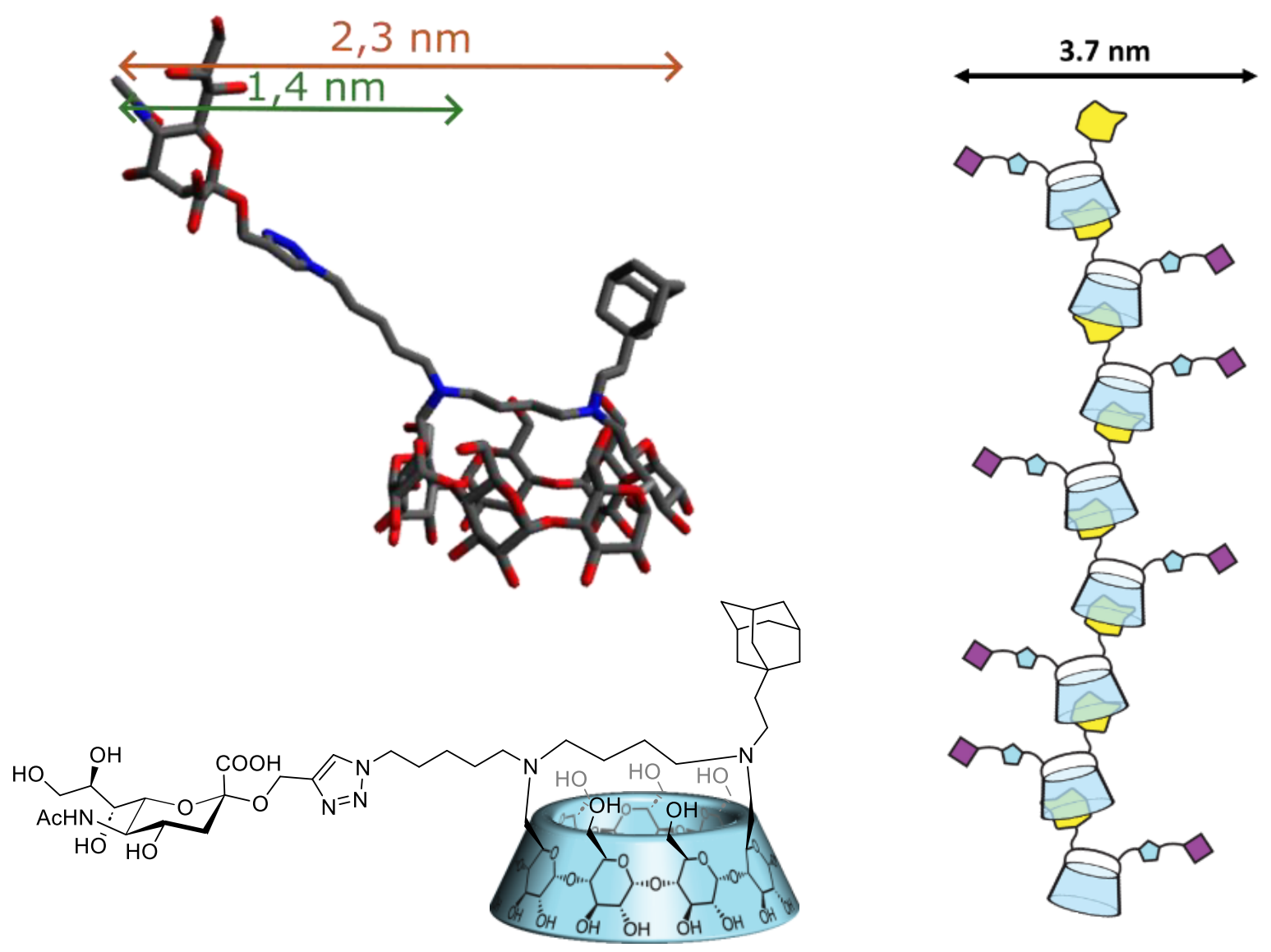

Fig. SI.4.1. Simulation of the diameter of a cylinder-like assembly of **CD2**.

The value of *p* can be determined graphically by plotting equation (3), and looking for the intersection with the *D* value experimentally found in the DOSY experiment, as shown below, for *d* = 3.7 nm:

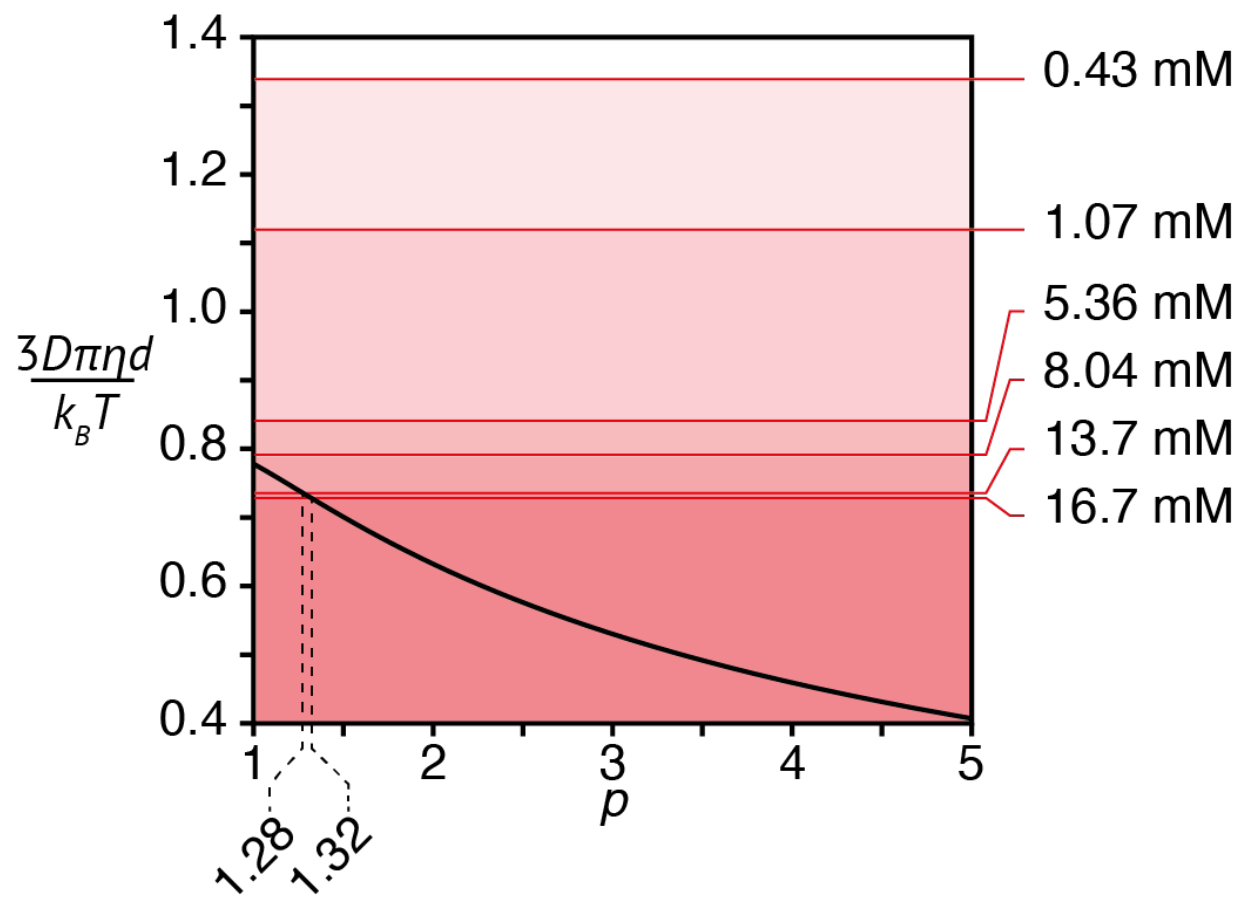

Fig. SI.4.2 Approximation of the p value for the assemblies of a series of concentrations of **CD2** following a DOSY-NMR set of experiments.

The *p* value has not been specified for those cases where it would be below 1, which would mean $d \geq L$, which corresponds to a single (non-assembled) unit of **CD2**. For those *p* values above 1, the degree of polymerization (DP) at a given concentration can be obtained by considering that the dimension of the repeat unit in the self-assembled polymer of **CD2** is 0.9 nm.

Table SI.4.3. Estimation of the DPs of the supramolecular assemblies of **CD2** at different concentrations, for an estimated diameter d = 3.7 nm.

C / mM	$\frac{3D\pi\eta d}{k_B T}$	*p*	*DP*
0.427	1.34	< 1	/
1.07	1.12	< 1	/
5.36	0.841	< 1	/
8.04	0.791	< 1	/
13.7	0.728	1.32	3.85 ± 0.77
16.7	0.735	1.28	3.74 ± 0.75

SI.5 Cytopathic effect and cytotoxicity assay

Compound solutions. A stock solution of the compound to be tested (CD) was prepared in non-supplemented DMEM culture media (10 mM). This was further diluted with non-supplemented DMEM culture media into two subsequent stock solutions (1600 and 800 µM).

Viral suspensions. From a viral stock suspension (471 PFU/µL), two diluted suspensions were prepared in non-supplemented DMEM culture media (2 PFU/µL and 0.2 PFU/µL).

Plate preparation. In a 96-well plate, 50 µL of either sample stock solutions (1600 or 800 µM) were mixed with 50 µL of either viral stock solutions (2 PFU/µL or 0.2 PFU/µL), in 8 replicates, to test high and low CD concentrations (800 µM or 400 µM) with high and low viral loads (1 PFU/µL or 0.1 PFU/µL).

Positive and negative control (0% CPE and 100% CPE, respectively) were assessed using 8 wells loaded with DMEM only (1A-D and 12A-D), and 16 wells loaded with viral stock at an MOI 0.001 (6A-H) or 0.01 (7A-H).

Finally, the potential cytotoxicity of each CD was assessed (4 wells per CD) by loading the well with the CD alone diluted with DMEM instead of being mixed with the viral stock (wells 1E-H and 12E-H of the plate).

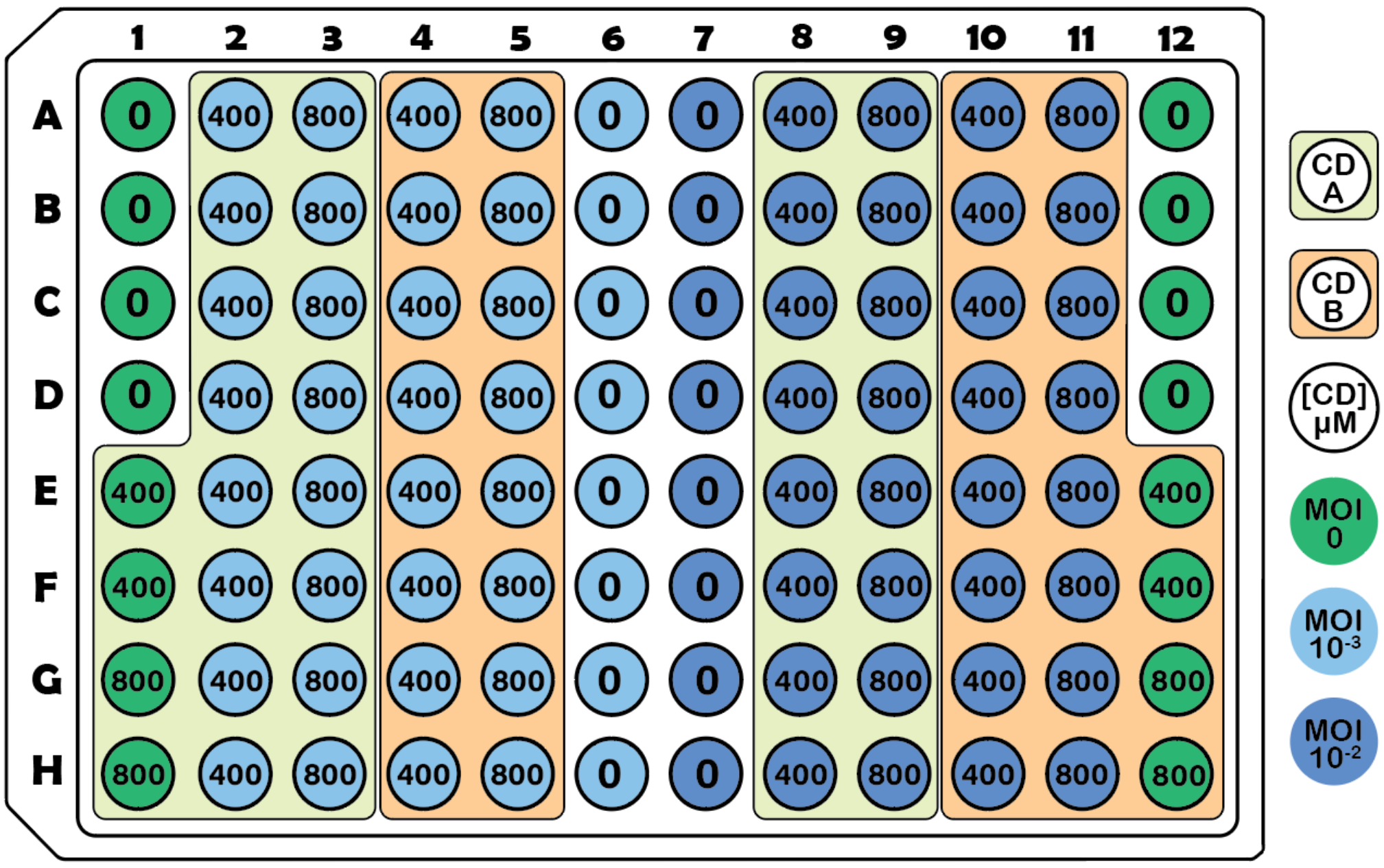

Fig. SI.5.1 Typical layout of a 96-well plate (viral incubation stage) for the study of CPE (SARS-CoV-2 on Vero E6 cells) in the presence of CDs (*i.e.* CD A and B). Wells in green did not contain any viral load, as a positive control of healthy cells ([CD] = 0 µM) to establish the 0% CPE, or to study the potential cytotoxicity of the different CDs ([CD] = 800 or 400 µM). In blue (light or dark) a viral load at MOIs of 0.01 or 0.001 was incubated with different CD concentrations ([CD] = 800 or 400 µM) or with no CD, as a negative control to establish the 100% CPE.

After a 1h incubation (37 °C, 5% CO_2), a Vero E6 cell suspension (100 µL at 10^5 cell/mL) was added to each well to test the efficiency of the assemblies at MOIs of 0.01 (100 PFU for 10^4

cells) or 0.001 (10 PFU for 10^4 cells). After a 4-day incubation (37 °C, 5% CO_2), the plates were visually analyzed using an inverted microscope to assess the level of cell death due to CPE (see manuscript for details).

SI.6 Cryo-EM analysis

Inactivated SARS-CoV-2 stock preparation

A viral stock of SARS-CoV-2 (*ca.* 2000 PFU/ µL) in DMEM (Dulbecco's Modified Eagle Medium, pH 7.4) was inactivated by exposure to UV (254 nm, 30 min.).

CD2 sample preparation

CD2 (*ca.* 0.1 mg) was weighted in an Eppendorf vial and subsequently diluted in the appropriate amount of DMEM (*ca*. 65 µL, final **CD2** concentration 800 µM). The sample was incubated at 37 °C without shaking over a period of 1 h before analysis by cryoEM.

CD2-virus sample preparation

CD2 (*ca.* 0.15 mg) was weighted in an Eppendorf vial and subsequently diluted in the appropriate amount of inactivated viral stock (*ca*. 65 µL, final **CD2** concentration 800 µM). The sample was incubated at 37 °C, 5% CO_2 without shaking over a period of 1 h before analysis by cryoEM.

Cryo-EM analysis

A 3 µL of the **CD2**-virus mixture was deposited on "quantifoil"® carbon membrane grids (Quantifoil Micro Tools GmbH, Germany). The excess of liquid on the grid was absorbed with a filter paper and the grid was flash-frozen in liquid ethane to form a thin vitreous ice film using a homemade mechanical cryo plunger. Once placed in a Gatan 626 cryo-holder cooled with liquid nitrogen, the samples were transferred in the microscope and observed at low temperature (-180 °C). Cryo-EM images were recorded on ultrascan 1000, 2k x 2k pixels CCD camera (Gatan, USA), using a LaB6 JEOL JEM2100 (JEOL, Japan) cryo-microscope operating at 200 kV with a JEOL low dose system (Minimum Dose System, MDS, JEOL, Japan) to protect the thin ice film from any irradiation before imaging and reduce the irradiation during the image capture.

CD2 alone (800 µM) in DMEM cell culture medium (10 min incubation time)

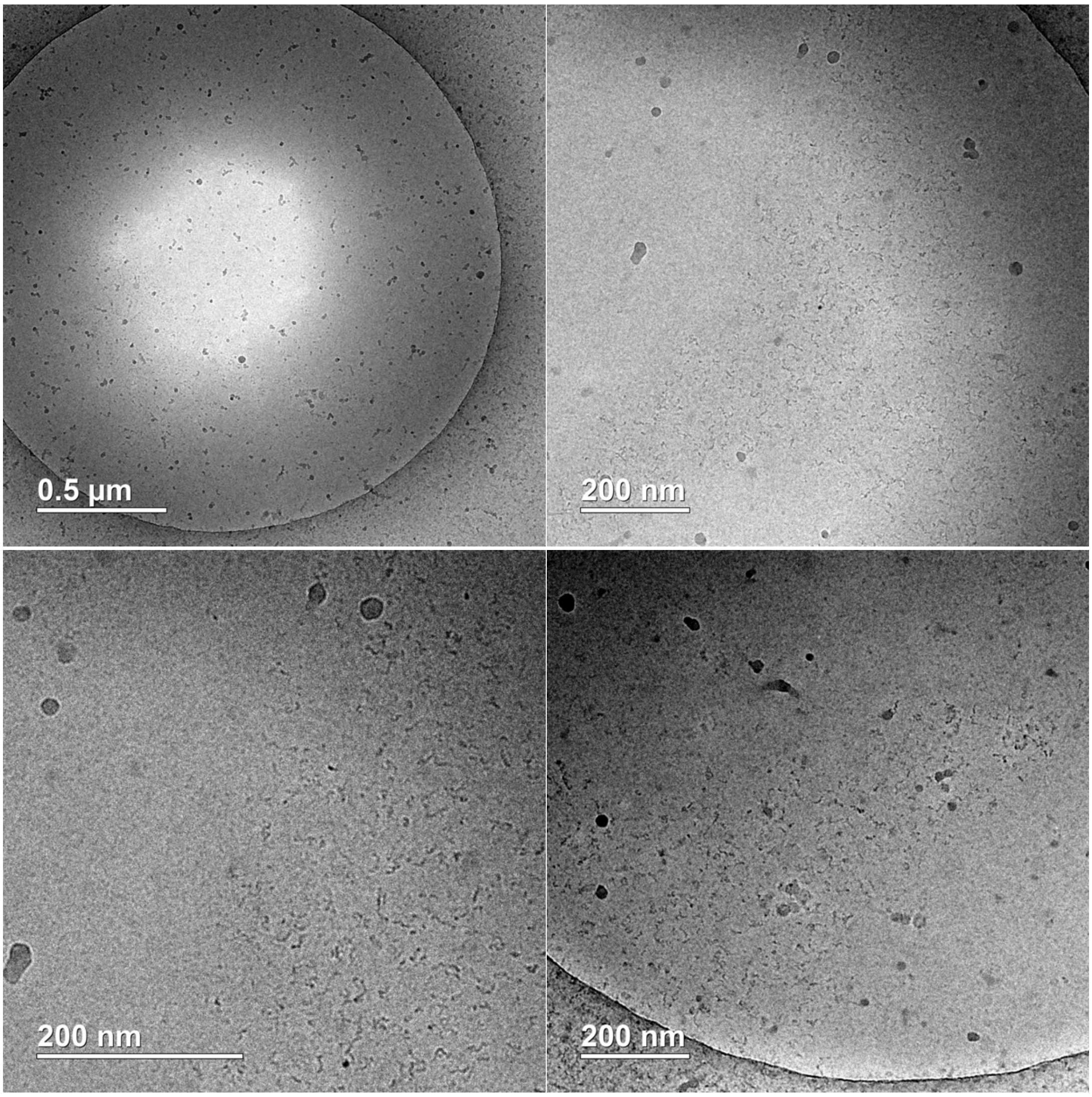

CD2 alone (800 µM) in DMEM cell culture medium (1 h incubation time).

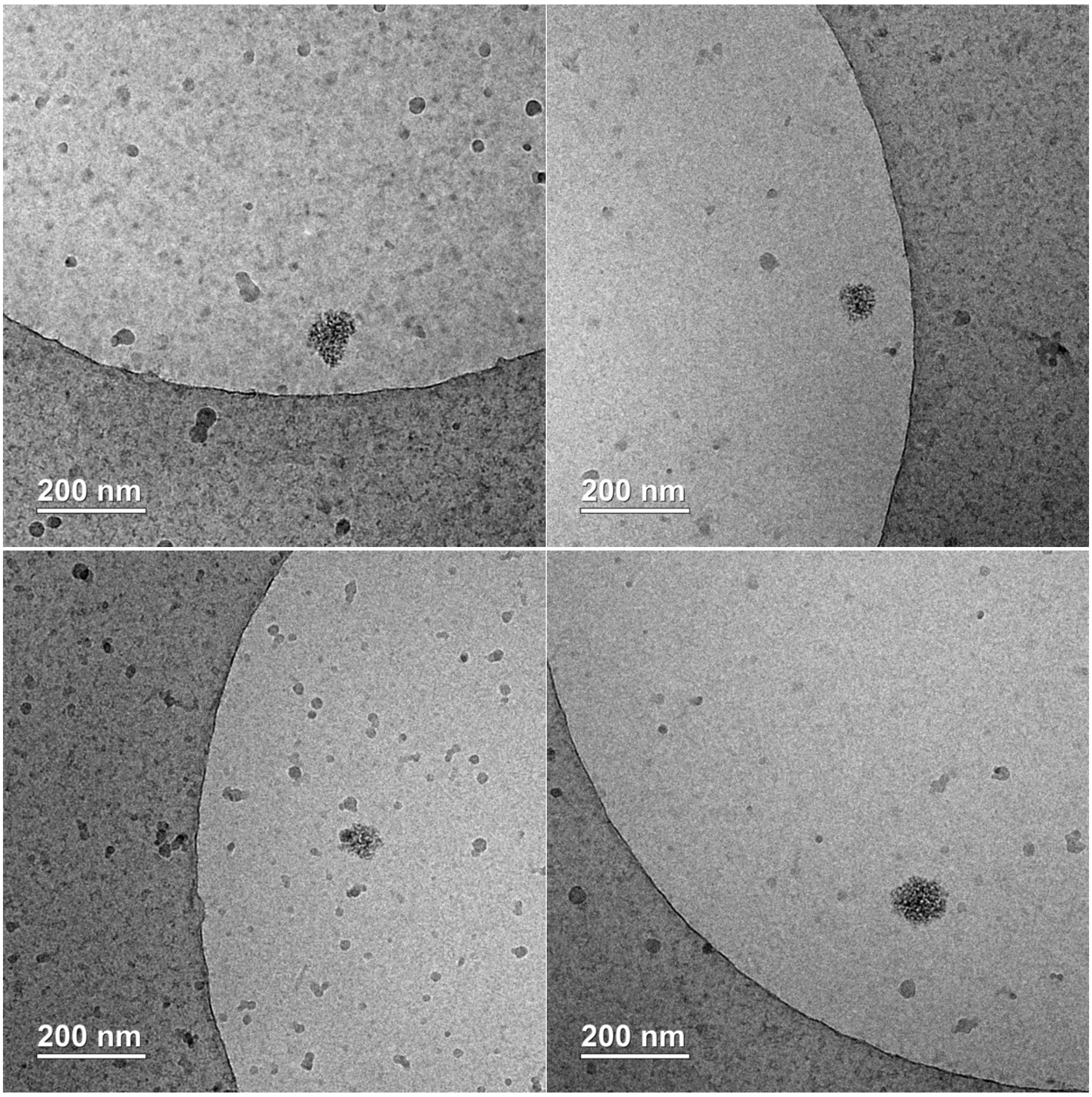

CD2 alone (800 µM) in DMEM cell culture medium (4 days incubation time).

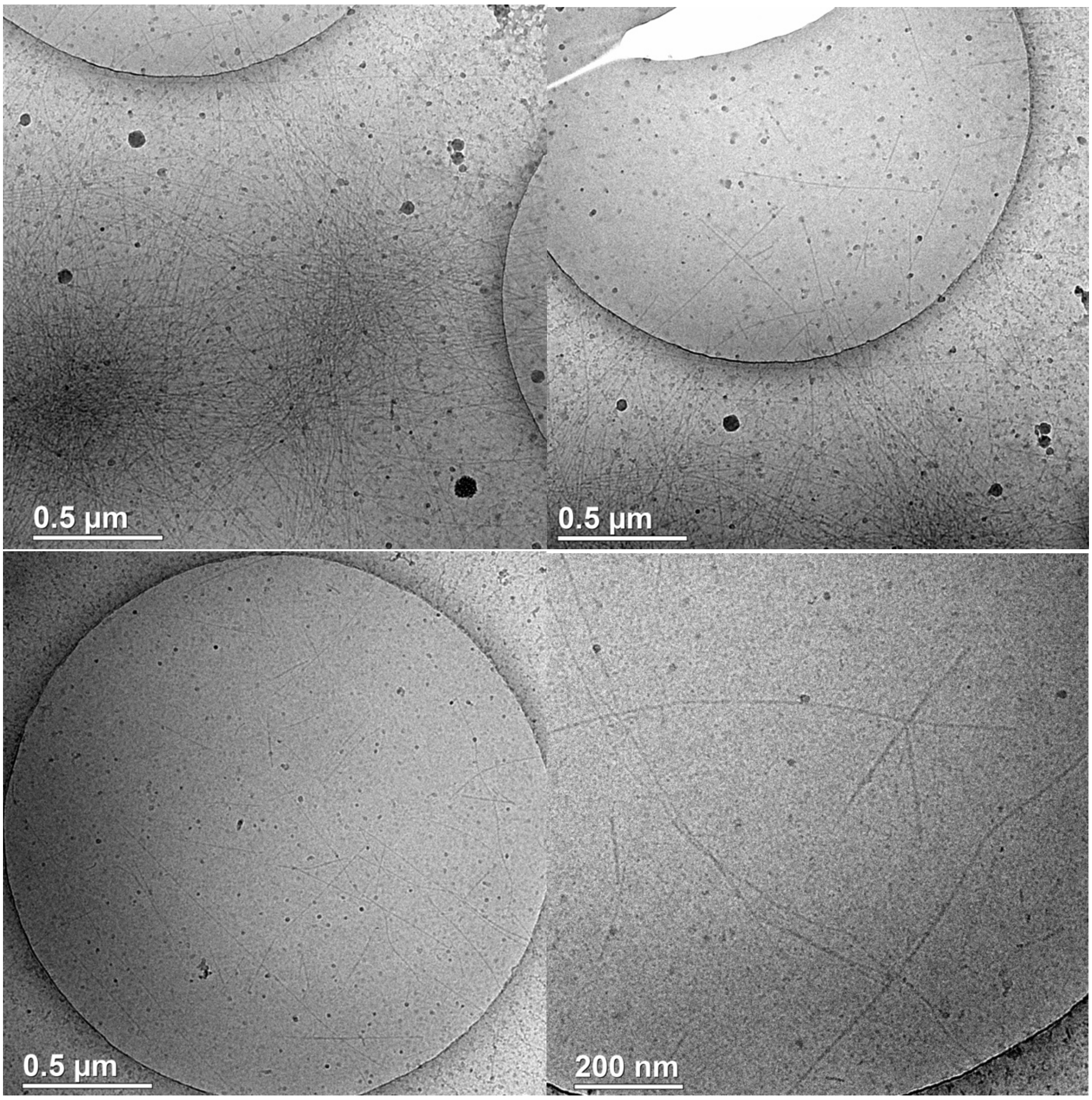

SARS-CoV-2 alone (2 500 PFU.µL^{-1}) in DMEM cell culture medium

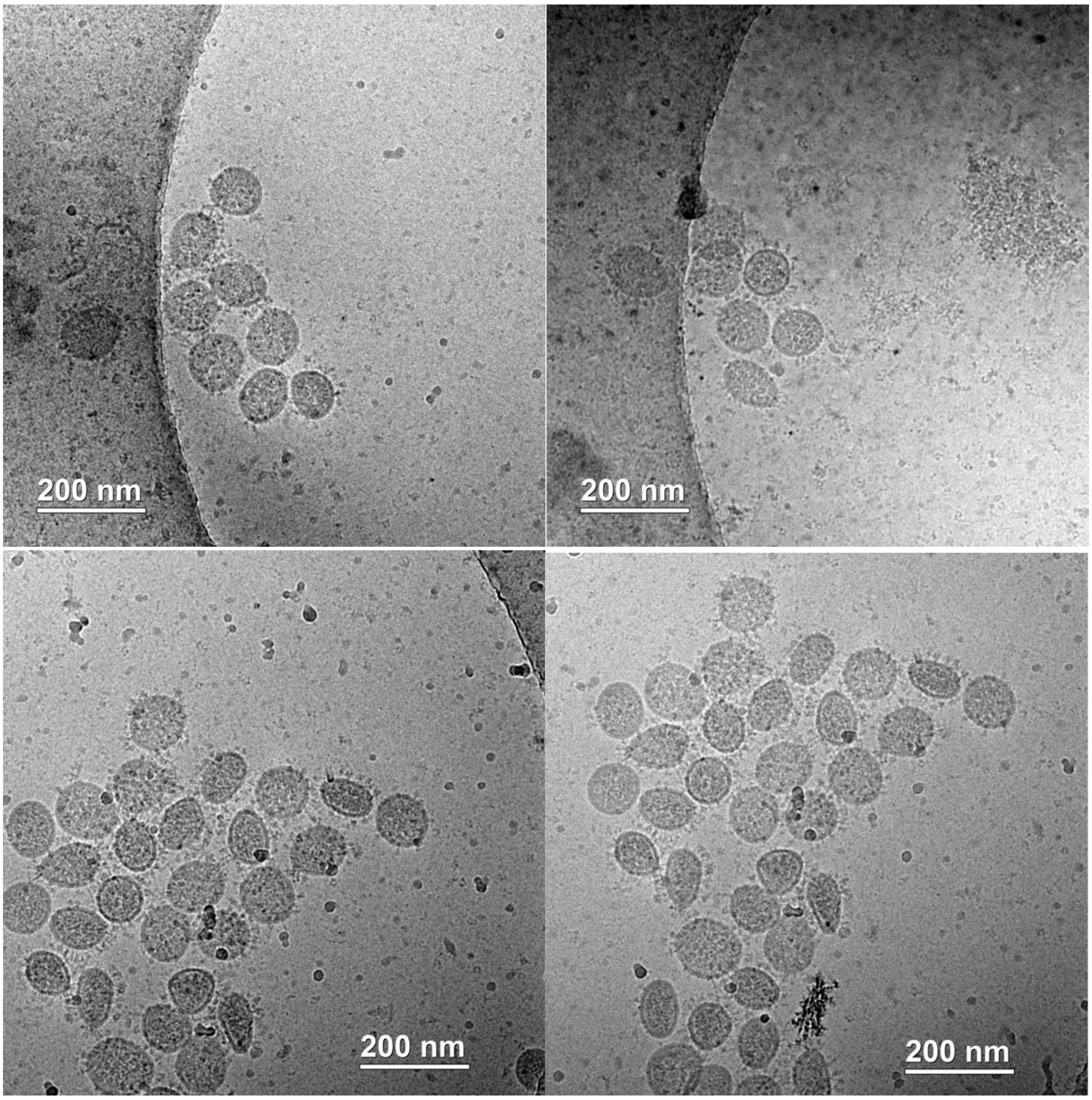

CD2 (800 µM) and SARS-CoV-2 (2 500 PFU.µL^{-1}) mixture in cell culture medium (1 h incubation time)

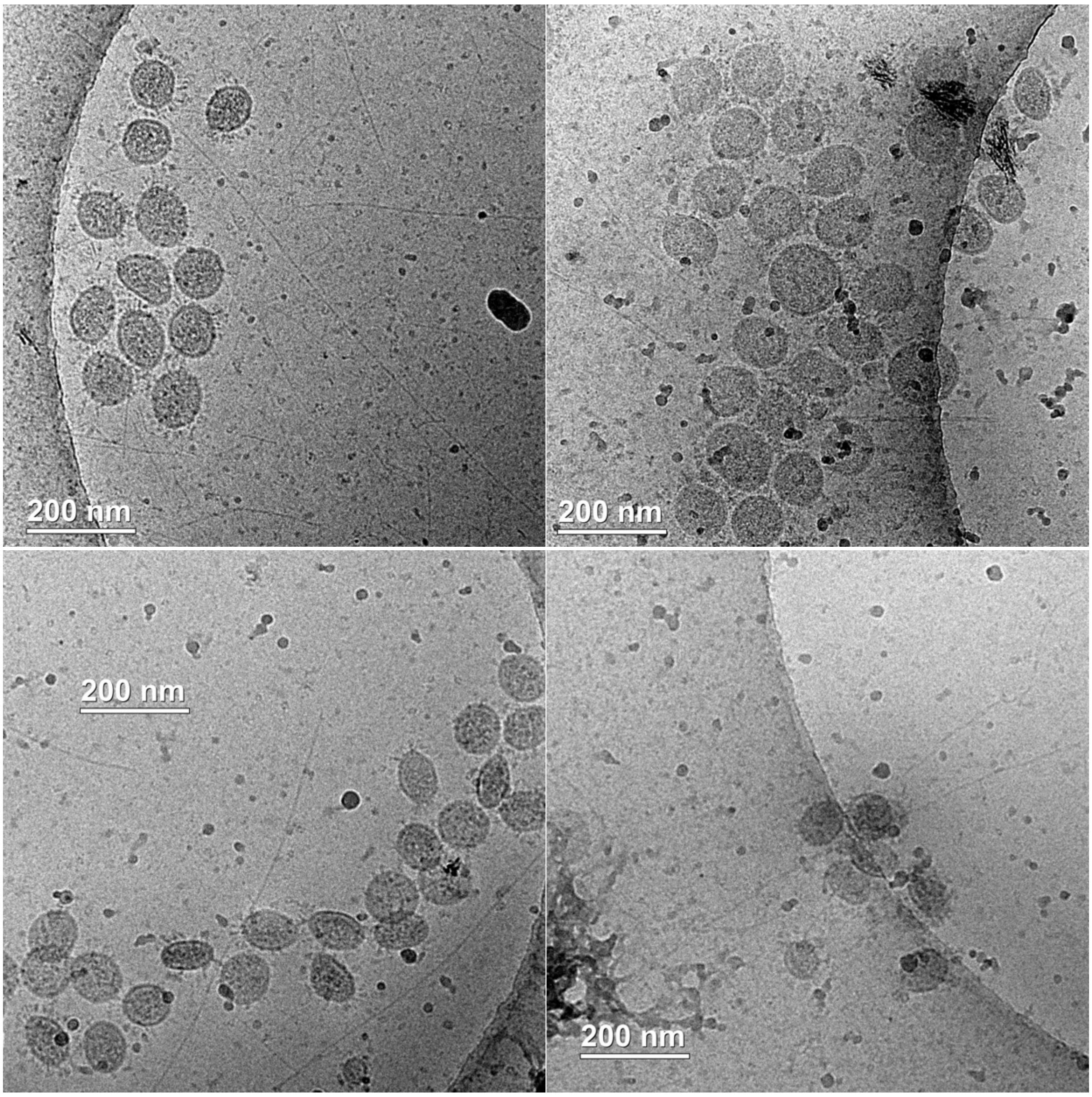

REFERENCES

[1] Field, Sensitive Dipstick Assays for Lectin Detection, Based on Glycan–BSA Conjugate Immobilisation on Gold Nanoparticles. P. J. Hernando, I. M. Ivanova, S. Chessa, M. J. Marín, S. Dedola, R. A. *Org. Chem. Front.* **2023**, *10*, 3819–3829. https://doi.org/10.1039/D3QO00424D.

[2] Cu Elimination from Cu-Coordinating Macromolecules. M. F. Ebbesen, D. Itskalov, M. Baier, L. Hartmann, *ACS Macro Lett.* **2017**, *6*, 399–403. https://doi.org/10.1021/acsmacrolett.7b00124.

[3] One-Pot Three-Enzyme Chemoenzymatic Approach to the Synthesis of Sialosides Containing Natural and Non-Natural Functionalities. H. Yu, H. A. Chokhawala, S. Huang, X. Chen, *Nat. Protoc.* **2006**, *1*, 2485–2492. https://doi.org/10.1038/nprot.2006.401.

[4] Immobilization of calix[4]arene-based glycoclusters on TiO_2 nanoparticlesvia click Cu(i)-catalyzed azide–alkyne coupling, L. Moni, S. Rossetti, M. Scoponi, A. Marra, A. Dondoni, *Chem. Commun.* **2010**, *46*, 475. https://doi.org/10.1039/B919353G

[5] Sialoside clusters as potential ligands for siglecs (sialoadhesins), Z. Gan, R. Roy, *Can. J. Chem*. **2002**, *80*, 908. https://doi.org/10.1139/v02-053

[6] Bridging β-Cyclodextrin Prevents Self-Inclusion, Promotes Supramolecular Polymerization, and Promotes Cooperative Interaction with Nucleic Acids. P. Evenou, J. Rossignol, G. Pembouong, A. Gothland, D. Colesnic, R. Barbeyron, S. Rudiuk, A.-G. Marcelin, M. Ménand, D. Baigl, V. Calvez, L. Bouteiller, M. Sollogoub, *Angew. Chem. Int. Ed.* **2018**, *57*, 7753. https://doi.org/10.1002/anie.201802550.

[7] Bio-inspired Total Synthesis of Twelve Securinega Alkaloids: Structural Reassignments of (+)-Virosine B and (−)-Episecurinol A. K. Antien, A. Lacambra, F. P. Cossío, S. Massip, D. Deffieux, L. Pouységu, P. A. Peixoto, S. Quideau, *Chem. Eur. J*. **2019**, *25*, 11574. https://doi.org/10.1002/chem.201903122

[8] Multivalent 9-O-Acetylated-Sialic Acid Glycoclusters as Potent Inhibitors for SARS-CoV-2 Infection. S. J. L. Petitjean, W. Chen, M. Koehler, R. Jimmidi, J. Yang, D. Mohammed, B. Juniku, M. L. Stanifer, S. Boulant, S. P. Vincent, D. Alsteens, *Nat. Commun*. **2022**, *13* . https://doi.org/10.1038/s41467-022-30313-8.

[9] A highly diastereoselective, practical synthesis of allyl, propargyl 2,3,4,6-tetra-O-acetyl-β-D-gluco, β-D-galactopyranosides and allyl, propargyl heptaacetyl-β-D-lactosides, H. B. Mereyala, S.R. Gurrala, *Carbohydr. Res.* **1998**, *307*, 351. https://doi.org/10.1016/S0008-6215(97)10104-5

[10] Synthesis of Sialic Acids, Their Derivatives, and Analogs by Using a Whole-Cell Catalyst. X. Lv, H. Cao, B. Lin, W. Wang, W. Zhang, Q. Duan, Y. Tao, X. W. Liu, X. Li, *Chem. Eur. J.* **2017**, *23*, 15143–15149. https://doi.org/10.1002/chem.201703083.

[11] Dimensions of Short, Rodlike Macromolecules from Translational and Rotational Diffusion Coefficients. Study of the Gramicidin Dimer. J. G. De La Torre, M. C. L. Martinez, M. M. Tirado, *Biopolymers* **1984**, *23*, 611–615. https://doi.org/10.1002/bip.360230402.
